# Untargeted and Targeted Cerebrospinal Fluid Neurometabolomics via Chromatography–Mass Spectrometry-Based Methods

**DOI:** 10.3390/molecules31111822

**Published:** 2026-05-25

**Authors:** Alisa K. Pautova

**Affiliations:** Federal Research and Clinical Center of Intensive Care Medicine and Rehabilitology, Petrovka Str. 25-2, 107031 Moscow, Russia; apautova@fnkcrr.ru; Tel.: +7-9057738981

**Keywords:** central nervous system, metabolites, gas chromatography–mass spectrometry, liquid chromatography–mass spectrometry, targeted analysis, untargeted analysis, standardization, validation, biomarkers, diagnosis

## Abstract

Neuroscience is a rapidly advancing field; however, a comprehensive understanding of brain function at the molecular, cellular, and systems levels remains incomplete. Neurological and psychiatric disorders represent a major global health burden, highlighting the need for improved diagnostic and therapeutic strategies. Cerebrospinal fluid (CSF) is one of the most informative biofluids for investigating central nervous system (CNS) pathology due to its close biochemical relationship with brain tissue. Recent advances in neurometabolomics, defined as the comprehensive analysis of small-molecule metabolites in CSF, have been driven by the development of highly sensitive and informative mass spectrometry-based techniques. These approaches enable the identification of disease-associated metabolic signatures. This review summarizes current chromatography–mass spectrometry-based methods used in both untargeted and targeted CSF metabolomics, with particular emphasis on their analytical performance, reproducibility, and limitations. Special attention is given to method standardization and validation, as well as to the identification of reliable metabolic biomarkers for the diagnosis and monitoring of neurological disorders, including neurodegenerative, psychiatric, oncological, and neuroinflammatory diseases.

## 1. Introduction

Neuroscience is a rapidly advancing field; however, a comprehensive understanding of brain function at the molecular, cellular, and system levels remains incomplete. Neurological and psychiatric disorders pose a substantial global health burden, with estimated annual costs exceeding €800 billion and affecting hundreds of millions of people worldwide [1]. Major contributors to neurological burden include strokes, neonatal encephalopathy, migraine, Alzheimer’s disease and other dementias, diabetic neuropathy, meningitis, epilepsy, complications of preterm birth, autism spectrum disorder, and cancers of the nervous system. In addition, post-COVID-19 cognitive impairment has emerged as an area of growing concern. Despite significant progress, current therapeutic strategies are largely symptomatic, and the underlying mechanisms of many disorders, including multiple sclerosis, Parkinson’s disease, Alzheimer’s disease, and amyotrophic lateral sclerosis, remain incompletely understood [2]. Therefore, identifying reliable biomarkers for disease diagnosis, disease progression monitoring, and therapeutic response remains a major challenge.

The clinical diagnosis of neurological disorders relies on a combination of clinical assessment, medical history, neuroimaging, and laboratory testing. Analysis of biological fluids plays a central role in this process. Although blood-based biomarkers for central nervous system (CNS) diseases are increasingly being developed, for example, for early diagnosis of Alzheimer’s disease [3], their diagnostic applicability remains limited. In contrast, cerebrospinal fluid (CSF) is one of the most informative biofluids for studying CNS pathology due to its direct contact with the brain and spinal cord. CSF enables the detection of proteins, metabolites, and inflammatory markers, including those produced within the CNS. In adults, CSF is produced at a rate of approximately 0.4–0.5 mL/min, with a total volume of about 140 mL and a slightly higher production rate during the nighttime. As such, CSF reflects the biochemical milieu of the brain and spinal cord [4,5].

Routine CSF diagnostics are primarily focused heavily on life-threatening conditions such as bacterial and viral meningitis and encephalitis, where rapid pathogen identification using culture, polymerase chain reaction (PCR), or antigen-based methods is essential. Clinical guidelines often recommend CSF sampling upon hospital admission, before antibiotic administration, with specific sample volumes allocated for cytological, microbiological, molecular, and biochemical analyses [6]. In research settings, CSF samples are sometimes obtained from patients with suspected neurological conditions, such as meningitis, that are subsequently not confirmed; these samples are then used as control groups [7,8,9]. This approach reflects the ethical and practical challenges of obtaining CSF from healthy individuals, as lumbar puncture is an invasive procedure with potential complications, including post-lumbar puncture headache, bleeding, and infection [10]. Furthermore, the use of residual CSF samples after routine diagnostics often results in limited sample volumes, which imposes constraints on analytical strategies and experimental design.

In recent years, increasing attention has been directed toward the analysis of the CSF neurometabolome, defined as the comprehensive profile of small-molecule metabolites present in CSF. Metabolomics provides a snapshot of a biological system’s biochemical state and is sensitive to genetic, environmental, and pathological influences. In the context of neurological diseases, metabolomic studies have revealed alterations in energy metabolism, neurotransmitter pathways, and lipid homeostasis [11]. Advanced analytical techniques, particularly mass spectrometry (MS) and nuclear magnetic resonance (NMR) spectroscopy, enable high-throughput metabolomic profiling of CSF. While NMR-based approaches have been applied in several studies [12,13], this review focuses primarily on MS-based methods, although combined NMR–MS strategies are also considered where relevant.

Direct-infusion high-resolution MS has been successfully applied in metabolomic studies of plasma [14], dried blood spots [15], and CSF [16], including investigations of pre-analytical stability [17]. This approach offers advantages such as rapid analysis, simplified sample preparation, and relatively low operational costs. However, the lack of chromatographic separation results in limited peak resolution and increased susceptibility to ion suppression and matrix effects, significantly limiting its applicability for comprehensive untargeted metabolomic profiling [18].

MS coupled with chromatographic separation provides the sensitivity, selectivity, and coverage required for the analysis of small-volume CSF samples. Chromatographic techniques combined with high-resolution MS detectors are widely used for untargeted metabolomic profiling. In contrast, tandem MS systems are typically employed in targeted quantitative analyses of selected metabolites or metabolite classes. Gas chromatography–mass spectrometry (GC–MS) and high-performance/ultra-high-performance liquid chromatography–mass spectrometry (HPLC/UPLC–MS) together enable the analysis of a broad range of metabolites with diverse physicochemical properties [19].

GC–MS offers several advantages, including efficient separation of complex mixtures, reduced matrix effects, and highly reproducible electron ionization spectra supported by extensive spectral libraries. However, its application is limited to volatile or derivatizable compounds, and it requires consistent and well-controlled derivatization procedures [20]. Comprehensive untargeted analysis of CSF metabolites by GC–MS typically involves protein precipitation combined with liquid–liquid extraction (LLE), followed by a two-step derivatization process (oximation and silylation), advanced separation techniques such as comprehensive two-dimensional GC (GC × GC), and high-resolution mass analyzers, including time-of-flight (TOF) and Orbitrap systems [21]. In targeted analysis, more selective sample preparation and derivatization strategies are often employed in combination with single- (Q) or triple-quadrupole (QQQ) mass spectrometers [20]. Some advances in untargeted and targeted GC–MS metabolic profiling of CSF have been reviewed previously [22].

UPLC coupled with various MS detectors has significantly expanded the range of detectable metabolites, enabling the analysis of polar, thermally labile, and higher-molecular-weight compounds. In untargeted LC–MS workflows, protein precipitation with organic solvents, such as methanol, is commonly used. However, additional steps, such as drying and reconstitution, are often employed to improve metabolite coverage. In some cases, sample concentration and derivatization may be required to enhance sensitivity, particularly in targeted analysis. High-resolution MS/MS analyzers, such as Q–TOF and Q–Orbitrap instruments, provide mass-to-charge (*m*/*z*) measurements with high precision; however, high mass accuracy alone does not always ensure unambiguous metabolite identification, and chromatographic separation remains essential for resolving structural or optical isomers [19]. The most comprehensive metabolite coverage is typically achieved by integrating complementary analytical approaches, including NMR, GC–MS, and LC–MS, with reversed-phase (RP) chromatography for hydrophobic compounds and hydrophilic interaction liquid chromatography (HILIC) for polar metabolites.

**Figure 1:**
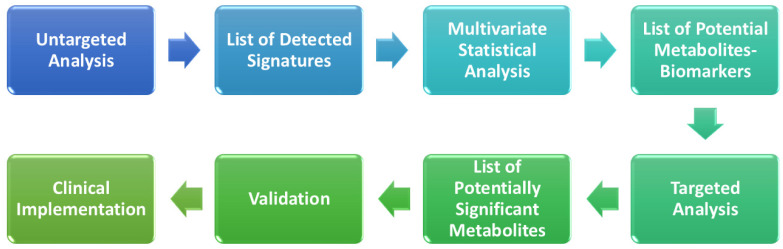
Untargeted and targeted approaches for new metabolite-biomarker investigation.

Untargeted metabolomics represents an initial step in identifying metabolic features that distinguish between physiological and pathological states without prior selection of specific analytes (Figure 1). This approach relies on broad extraction protocols and high-resolution analytical platforms, followed by multivariate statistical analysis to identify discriminative features for further investigation. Standardization in untargeted metabolomics can be guided by initiatives such as the Metabolomics Standards Initiative [23,24] or the requirements described in a metabolomics workflow [25].

Targeted metabolomics focuses on the quantitative analysis of predefined metabolites of interest, often selected based on prior biological or analytical evidence. This approach requires optimized sample preparation, selective detection methods, and robust calibration strategies. Quantification in CSF can be challenging due to the endogenous nature of many metabolites and the absence of suitable blank matrices [26]. Therefore, careful validation of analytical methods is essential. Regulatory guidelines, including those issued by the U.S. Food and Drug Administration (FDA) in 2018 [27], the European Medicines Agency (EMA) in 2012 [28] and the International Council for Harmonization (ICH M10) in 2023 [29], define key validation parameters such as accuracy, precision, selectivity, matrix effects, and stability. Only rigorously validated methods are suitable for clinical applications. Despite substantial progress, many proposed metabolite biomarkers remain in the validation stage due to factors such as biological variability, analytical complexity, and regulatory requirements, highlighting the gap between biomarker discovery and clinical implementation [30].

This review aims to summarize current challenges and emerging solutions in chromatography–MS-based CSF metabolomics, with a particular focus on improving analytical reproducibility. In addition, the review discusses metabolic alterations and potential biomarkers associated with various neurological disorders. Most of the studies included in this review have been published since 2010 and reflect recent advances in the field. Literature searches were conducted using PubMed, Scopus, and Google Scholar databases with combinations of keywords including “CSF”, “mass spectrometry”, “metabolomics”, and “metabolites”.

## 2. Untargeted Metabolomics

Untargeted global metabolic profiling aims to detect and annotate as many metabolites as possible. According to the Human Metabolome Database, of the 248,138 metabolites currently cataloged, 452 have been identified in CSF to date [31]. However, ongoing studies continue to report additional CSF metabolites, indicating that the CSF metabolome remains incompletely characterized.

**Figure 2:**
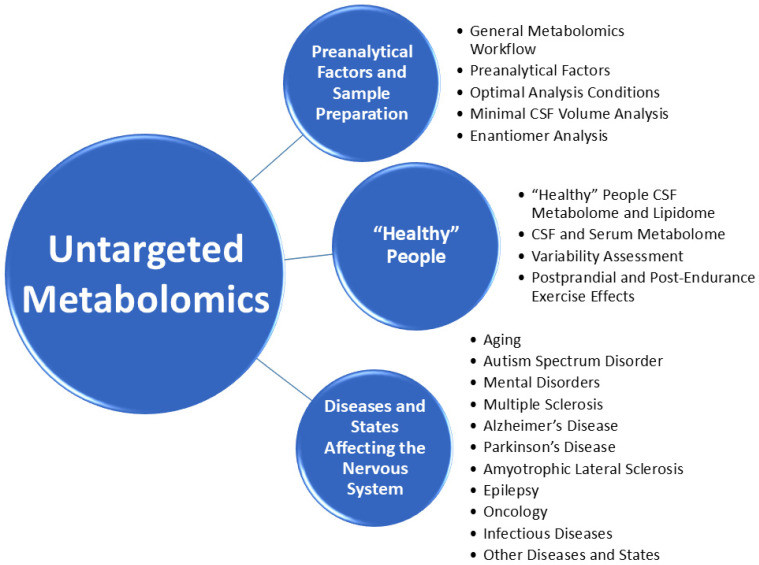
The main aspects covered in Section 2, Untargeted Metabolomics.

During sample collection, storage, preparation, and analysis, metabolites may undergo structural changes or differ in polarity and chemical reactivity, contributing to variability in MS-based results. This section outlines key aspects of the metabolomics workflow, highlights critical preanalytical factors, and summarizes the most effective sample preparation strategies, including those requiring minimal CSF volumes. In addition, findings from clinical studies involving both “healthy” individuals and patients with neurological disorders are discussed (Figure 2).

### 2.1. General Analytical Approaches for Metabolomics Studies

Although the Metabolomics Standards Initiative (MSI) was proposed in 2007 [23,24], analyses of studies published over the past decade indicate that its recommendations have not been consistently followed [32]. Nevertheless, standardization of untargeted metabolomics workflows remains essential. In 2021, Alseekh et al. proposed comprehensive guidelines for MS-based metabolomics, emphasizing the importance of method reproducibility, the use of quality control (QC), and the assessment of ion suppression [25]. Given the breadth of these guidelines, only key aspects relevant to CSF analysis are summarized below:

- Quenching: Rapid termination of metabolic activity immediately after sampling and during storage is critical to prevent artifacts. Quenching requires strict control of timing and temperature. Efficiency may be evaluated using isotopically labeled standards.
- Replicates and QC samples: Using biological replicates (at least 4 sources) helps assess variability, while analytical and technical replicates evaluate equipment performance and procedural consistency. QC samples enable batch correction and monitor stability throughout analysis.
- Randomization: Random sampling minimizes bias due to instrument drift or batch effects. Stratified or blocking randomization strategies should be employed, especially in large or multi-group studies, often in conjunction with QC injections to correct for drift.
- Quantification: Relative intensity measures in chromatography-MS reflect metabolite abundance but may not directly correspond to concentrations, especially without calibration curves and pure standards. Dilution experiments can test detection linearity.
- Ion suppression: Matrix effects significantly influence ionization efficiency, especially in LC–MS, potentially leading to quantification errors, and improved sample preparation and chromatographic separation are required. Electrospray ionization (ESI) in the negative mode may result in less ion suppression.
- Peak identification: Challenges include separating isomers (see below for more detailed information on optical isomer analysis), resolving overlapping peaks, and avoiding artifacts such as degradation or in-source formation. Proper chromatographic techniques are essential for accurate identification.
- Data reporting: Clear presentation of metabolite information (name, *m*/*z*, retention time, chemical formula, and fragmentation pattern) is critical for interpretability and reproducibility.

An approach reducing the ion suppression in untargeted analysis was proposed in 2012 [33]. Initially developed for cell culture analysis, it was later extended to tissue and biofluid analysis. For cell cultures, it included isotopic ratio outlier analysis using 13C-labeled standards to grow the exact cell culture analog, followed by the comparison of experimental and control samples. For biological fluid analysis, a commercially available, isotopically labeled “artificial metabolome” from *Saccharomyces cerevisiae* S288C containing hundreds of 13C-enriched metabolites is added to each biological sample. This mixture serves as an internal standard, a reference library for metabolite identification, and a calibration system for quantitative MS measurements, thereby enabling better untargeted analysis. In a recent study, the potential of this approach for ion chromatography, RP LC–MS, and HILIC LC–MS was described [34]. While this approach improves data quality, its widespread use is limited by cost and storage requirements.

Strict adherence to standardized workflows, such as [25], improves reproducibility and comparability across studies. However, many CSF metabolomics studies still lack sufficient methodological detail, raising concerns about data reliability. Although these recommendations are general, specific studies have investigated optimal conditions for sample storage and preparation in CSF analysis.

#### 2.1.1. Preanalytical Factors

Preanalytical factors significantly affect the stability of metabolites and amino acids in CSF. Delays in storage (at room temperature for 30 or 120 min), absence of centrifugation, storage at 4 °C, and multiple freeze–thaw cycles can alter metabolite concentrations (changes in the concentrations of 49 metabolites and the increase in 10 amino acids were observed), mainly due to leukocyte activity. Immediate centrifugation, snap-freezing in liquid nitrogen, and storage at −80 °C are recommended to preserve sample integrity [35]. More general recommendations for CSF collection and analysis state that the time and conditions of collection should be recorded; CSF sampling should be performed only in polypropylene tubes; and the use of additives during collection is not recommended unless specifically intended. Centrifugation should be performed at 2000× *g* for 10 min at 4 °C. Furthermore, delays in transportation and subsequent analysis should be minimized [36].

#### 2.1.2. Optimal and Minimal CSF Volume Conditions

Optimal conditions for protein precipitation prior to CSF analysis using pure organic solvents or mixtures, followed by reconstitution with water or mixed solvents, depend on the LC method (RP or HILIC). Song et al. studied various combinations of solvents for protein precipitation and reconstitution suitable for RP or HILIC UPLC–Q–TOF, and found that MeOH–EtOH–ACN (1:1:1, *v*/*v*/*v*) for the precipitation, together with H_2_O–MeOH–ACN (2:1:1, *v*/*v*/*v*) for the reconstitution, resulted in 722 detected metabolites using RP analysis. In contrast, EtOH for protein precipitation and H_2_O–MeOH–ACN (2:1:1, *v*/*v*/*v*) for reconstitution yielded 566 detected metabolites by HILIC analysis [37]. Two-phase LLE methods, such as Folch, Bligh and Dyer (B&D), and Matyash, were tested for simultaneous lipid and metabolite analysis in CSF. Acidified B&D (aB&D), i.e., a CHCl_3_/MeOH (1:2, *v*/*v*) mixture acidified with 1% acetic acid, was found to improve lipid recovery. The following analysis of the organic phase by RP and aqueous phase by LC–Q–Orbitrap, together with the GC–Q–TOF analysis of the dried and derivatized organic and aqueous portions, demonstrated the highest reproducibility and broad detection of up to 700 metabolites across different classes [38]. For hydrophilic metabolite profiling, nanoLC coupled to QQQ or Q–TOF was evaluated using various columns, stationary phases, and other chromatographic parameters. A method employing Primesep A column with a polyethylene glycol-coated capillary and a two-step trifluoroacetic acid gradient in MeOH as the mobile phase successfully separated 33 metabolites from 10 µL of CSF. Analysis of samples from Alzheimer’s patients and controls identified hundreds of peaks and potential biomarkers, with results bolstered by isotopically labeled internal standards and peak normalization [39].

Methods requiring minimal CSF volume are highly relevant given limited CSF sample availability, particularly in animal models or pediatric studies. Further, this review will present various clinical studies that used approximately 10 µL of CSF for UPLC–MS-based analysis, but they did not focus on developing or optimizing the analytical method; thus, they are not discussed here. Regarding volatile metabolites detected by GC–MS, the most common CSF volume for analysis is about 100–200 µL, and approaches utilizing smaller volumes are attractive. A notable untargeted GC–TOF metabolomics study analyzed only 5 µL of CSF from patients with aneurysmal subarachnoid hemorrhage. Using ACN:isopropanol:H_2_O (3:3:2, *v*/*v*/*v*) extraction, 97 metabolites were identified, mainly amino acids, of which 16 were significantly altered during vasospasm. Derivatization details were not specified; however, the recommendations of the Metabolomics Standards Initiative were followed [40]. Another GC–MS method employed a modified vial design that allowed analysis of only 10 µL of CSF. Using minimal MeOH for protein precipitation and standard two-step derivatization (oximation and silylation) yielded 73 metabolites, comparable to the coverage in larger CSF samples (≥100 µL). It maintained coefficients of variation (CVs) below 20% for most metabolites [41]. Further development applied in-liner silylation with GC–TOF, using as little as 0.01–2 µL of CSF samples. It detected over 340 signatures in human CSF, with high reproducibility (CV < 15% for most). However, silylation without prior oximation led to isomer formation for monosaccharides and similar compounds with multiple reactive groups, and the approach with in-liner derivatization, while sensitive, demands rather expensive equipment [42]. Thus, the GC–MS conditions described in the previous study [41] appear to be more optimal. Also, a GC–MS study on a naturally occurring depressive model in macaques used 15 µL of CSF [43]; however, no specific attention was paid to the analysis, and this study will be discussed in Section 2.4.

#### 2.1.3. Enantiomer Analysis

Metabolite enantiomers, such as d- or l-amino acids or carboxylic acids, or their chiral metabolites, both separately and in their ratio, may provide additional important information during metabolic profiling. While l-isomers are widespread in eukaryotic cells, d-isomers, previously thought to be restricted to bacteria, are rare, and their abundance and functions are poorly understood. For example, d-serine modulates neurotransmission in the brain, and d-aspartate is present in neuroendocrine tissues, while the enzymes involved in their synthesis and degradation are implicated in some neural diseases [44]. Thus, the sensitive chiral and achiral analysis in untargeted metabolomics, especially its application to CSF analysis, is of particular interest [45]. There are two common approaches for chiral analysis: chiral derivatization during sample preparation or chiral stationary phases in HPLC columns [46]. The study [45] describes the use of derivatization with two specially synthesized native and 13C-labeled reagents for the amino acids and carboxylic acids. The samples are subsequently combined and analyzed by UPLC–Q–TOF. Then specialized software detects the resulting isotope pairs to improve metabolite identification, exclude artifacts, enable relative quantification, and distinguish D- and L-enantiomers. The method was applied to CSF samples from patients with Alzheimer’s disease and controls, yielding the detection of more than 400 carboxylic acid metabolites and more than 600 amine-containing metabolites. From 24 potential biomarkers, eight were chiral metabolites, such as d-kynurenine and l-tryptophan, demonstrating that metabolic changes in the disease also affect the enantiomeric composition of molecules. A limitation of the developed approach for widespread use is the current lack of commercially available isotopically labeled chiral derivatization reagents that enable the simultaneous, artifact-free detection of both enantiomer families across hundreds of metabolites. While various chiral derivatization agents exist, particularly, tested for CSF targeted analyses (e.g., Marfey’s reagent [47], (+)-1-(9-fluorenyl) ethyl chloroformate [48]), they generally do not provide the isotopically labeled binding required for robust untargeted screening, making the broader application of untargeted chiral metabolomics currently dependent on custom-synthesized reagents, highlighting a gap in the commercial market.

#### 2.1.4. Chemometrics for Untargeted Metabolomics

Chemometric approaches play a vital role in untargeted metabolomics due to the high dimensionality and complexity of chromatography–MS datasets. Raw metabolomics data typically contain thousands of features with substantial noise, batch effects, missing values, and nonlinear relationships between metabolites, making advanced statistical and machine learning methods essential for reliable data interpretation. Chemometric workflows generally include baseline correction, noise filtering, peak detection, deconvolution, alignment, normalization, dimensionality reduction, variable selection, and multivariate modeling [49,50]. Commonly used supervised and unsupervised approaches include principal component analysis (PCA), partial least squares-discriminant analysis (PLS–DA), orthogonal PLS (OPLS), random forest (RF), support vector machines (SVM), and some other [49]. In metabolomics studies, these methods are applied not only for classification and biomarker discovery, but also for peak annotation, retention time prediction, missing value imputation, and integration of multi-omics datasets [49]. Several open-source platforms, including XCMS, MZmine, OpenMS, and MS–DIAL, incorporate chemometric algorithms for preprocessing and feature extraction [50]. However, despite the broad availability of computational tools, overfitting, insufficient validation, and poor interpretability of models remain major concerns, particularly in studies with limited sample sizes, which are common in CSF metabolomics. Therefore, rigorous cross-validation, appropriate normalization strategies, inclusion of QC samples, and transparent reporting of statistical workflows are essential for improving reproducibility and biological interpretability of metabolomics data [49,50].

#### 2.1.5. Practical Considerations for Standardization in CSF Untargeted Metabolomics

Although general guidelines for metabolomics studies have been published, their direct application to CSF is hindered by several matrix-specific limitations. These include the typically small sample volume (often 50–200 µL or less), the invasive nature of lumbar puncture (which limits access to large healthy control cohorts), and the absence of a true analyte-free matrix due to the endogenous nature of most CSF metabolites.

For pilot studies or investigations with very limited CSF volumes (≤100 µL per sample), a basic set of requirements can be considered. These include documentation of preanalytical conditions: time to freezing, tube type, presence or absence of red blood cells, and centrifugation regime), fast quenching and freezing; sample preparation conditions: clear reporting of extraction and/or protein precipitation solvent composition, and additional steps; chromatographic conditions: chromatographic column and composition of mobile phases/flow rate. During the analysis: regular injection of pooled CSF QC samples (every 6–10 injections) to monitor instrument drift, randomization of samples across analytical batches. Where possible, deposition of raw data in public repositories is encouraged even at this basic stage.

For discovery-oriented studies with larger CSF volumes (≥100 µL per sample), an extended set of requirements is advisable. In addition to the basic elements listed above, such studies should include at least four biological replicates per group, assess analytical variability using technical replicates (minimum of three measurements per QC sample), and employ a panel of stable isotope-labelled internal standards covering major compound classes (e.g., amino acids, organic acids, lipids). Conducting a power analysis that justifies the sample size will enhance the significance of the results obtained across groups.

Regardless of the chosen level of standardization, the rationale for sample volume and preparation method should always be provided. The proposed gradation does not replace existing guidelines but rather adapts them to the practical realities of CSF analysis, thereby improving transparency and cross-study comparability without imposing unrealistic requirements on studies with severe volume limitations.

### 2.2. “Healthy” People CSF Metabolome

CSF metabolomics of “healthy” individuals screened for meningitis (with subsequent negative results) was performed by the creators of the Human Metabolome Database and highlighted the use of diverse techniques, including GC–MS, NMR, and LC–MS. A pioneering 2008 study identified over 70 metabolites [7]. Further studies expanded the database to over 450 metabolites, revealing significant variability (up to ±100%) among individuals, particularly for acetone, glutamic acid, and acetoacetic acid [51]. In a metabolomics study on healthy people, plasma and CSF LC–TOF analyses revealed similar analytical (CV around 25%) and biological (CV between 35 and 46%) variability [52]. However, in an untargeted and targeted (quantified but not fully validated) metabolomics study on donors’ serum and CSF, GC × GC–TOF analysis resulted in higher serum biological variability (CV 26–180%) than CSF (CV 17–130%), with predominantly higher metabolite concentrations in serum than in CSF. It should be noted that the use of such a comprehensive analysis as GC × GC–TOF with cryogenic modulation resulted in better separation and detection of substances previously undescribed for CSF, like 4-hydroxypentanoic, 2-ethyl-3-hydroxypropionic, and 4-methyl-2-oxovaleric acids [53]. Another donors’ CSF metabolomics study using GC–MS, NMR, and LC–MS/MS described the biological variability of almost 130 metabolites, ranging from 15 to 85%, with analytical variability < 20% [54]. In a broadly polar, untargeted HILIC–Q–TOF CSF study in positive and negative ESI modes, gender-associated metabolic changes were observed in cognitively healthy individuals, mostly over 60 years old, yielding around 150 detected metabolites, with acylcarnitines, uric acid, and hydroxyvaleric acid higher in men, and taurine higher in women. Analytical variability was higher in negative ESI mode (CV up to 35%) than in positive mode (CV < 8%), while biological variability was <50% for most metabolites [55].

Light-dark phase CSF metabolic and transcriptomic changes were evaluated in rats, which showed changes similar to those in humans in day-night CSF production. Of the 126 metabolites detected by UPLC–Q–Orbitrap, 19 showed changes between the light and dark phases: 5 metabolites were higher at night, while 14 were higher in the light phase [56]. Additionally, healthy volunteers’ CSF analysis using GC–MS/MS, HILIC–UPLC–MS/MS, and LC–Orbitrap indicated minimal postprandial effects on CSF metabolomics (unlike plasma) consisted of 150 hydrophilic and more than 260 hydrophobic metabolites with some metabolites and lipids being statistically different among various time points [57]; while intense endurance exercise induced notable metabolic shifts detected by LC-MS/MS—particularly in purines, pyrimidines, amino and fatty acids and their derivatives, and Krebs cycle intermediates, a total of 30 CSF metabolites from more than 300—affecting both CSF and plasma [58].

Although CSF lipidomics is not the main focus of this review, Section 3, will include a wide range of protocols for lipid-derived metabolites, and one relevant study provides useful insights into the CSF lipidome. Analyzing samples from patients with neurological symptoms and controls via a validated aB&D method and RP–UPLC–Orbitrap, researchers identified about 550 lipids across five classes: free fatty acids, glycerophospholipids, sphingolipids, glycerolipids, and sterol lipids. MS/MS analysis improved lipid annotation, revealing that roughly 50% of CSF lipids are isobaric ions [59].

### 2.3. Aging

Aging is not classified as a disease; nevertheless, it triggers irreversible changes throughout the body that are associated with the development of various health conditions. MS-based CSF metabolomics has been employed in three studies to identify potential biomarkers of aging. The first study analyzed CSF from 23 healthy people aged 30 to 74 using HPLC–Q–Orbitrap, uncovering that eight metabolites from 70, namely isoleucine, acetylcarnitine, pipecolate, methionine, glutarylcarnitine, 5-hydroxytryptophan (also higher in females), ketoleucine, and hippurate, increased with age. At the same time, methylthioadenosine and 3-methyladenine decreased [60]. The second study included 85 healthy individuals aged 20–86, 57 patients with Alzheimer’s disease, and 56 patients with Parkinson’s disease, employing targeted but not quantitative (HILIC–UPLC–QQQ) and untargeted (HPLC–Q–TOF) metabolomics, as well as lipidomics. It identified elevated levels of kynurenine, xanthine, 5-hydroxyindole-3-acetic acid (5-HIAA), carnitine, and cystine as markers of advanced age, while higher concentrations of serine, 4-aminobutyric acid, and uridine were associated with younger individuals [61]. The third study analyzed CSF from 92 healthy adults grouped by age and identified approximately 40 metabolites among more than 160 using UPLC–Q–TOF that were positively correlated with age, including pantothenic acid, aspartic acid, cysteine, 5-HIAA, and glutamate. At the same time, asparagine and glycerophosphocholine showed negative associations. Elevated levels of taurine and 5-HIAA were observed in females [62]. Collectively, these highly valuable (since studying healthy individuals) findings suggest that aging is associated with increased levels of metabolites such as 5-HIAA, cysteine, and aspartic acid, reflecting processes including blood–brain barrier disruption, neuroinflammation, and mitochondrial decline.

Since the kynurenine pathway metabolites were mentioned above in studies on aging as possible markers, a systematic review of their relationship with aging may be of interest. The authors analyzed studies on CSF and serum concentrations of these metabolites. They summarized that CSF and serum tryptophan and kynurenine are higher in older people, with elevated levels of kynurenic and quinolinic acids and other kynurenine pathway metabolites, demonstrating mixed results [63].

Special attention should be paid to a metabolome-wide association study (MWAS) [64] that included CSF samples from almost 700 cognitively healthy participants, mostly over 60 years old, from the Wisconsin Alzheimer’s Disease Research Center [65] and the Wisconsin Registry for Alzheimer’s Prevention [66] studies. A UPLC–MS/MS untargeted CSF analysis resulted in 338 metabolites and 19 metabolite–phenotype associations, such as *N*^6^-methyllysine, *N*-delta-acetylornithine, alphatocopherol, guanosine, malate, ethylmalonate, and 2-hydroxy-3-methylvalerate associated with schizophrenia; *N*-delta-acetylornithine, benzoate, and glutaroylcarnitine [C5] associated with cognitive performance; *N*-delta-acetylornithine, cysteinylglycine, and glycerol associated with alcoholic drinks per week; ethylmalonate associated with smoking behavior; cysteinylglycine disulfide associated with sleep duration; orotate and malate associated with attention deficit hyperactivity disorder. After adjusting for multiple testing, four associations remained: *N*-delta-acetylornithine, *N*^6^-methyllysine, and ethylmalonate, all associated with schizophrenia; and *N*-delta-acetylornithine, associated with cognitive performance [64].

### 2.4. Autism Spectrum Disorders and Depression Disorders

The MWAS [64] used metabolomics data from a publicly available genome-wide association studies (GWAS) [67], similar to another study, in which CSF metabolomics data were used to build metabolite predictive models for anxiety disorder based on dopamine 3-*O*-sulfate, major depression disorder based on 1-linoleoyl-gpc, *S*-methylcysteine, and *N*^1^-methylinosine, and autism spectrum disorder based on *p*-cresol sulfate and myo-inositol [68].

Although autism spectrum disorder includes challenges in behavior, learning, speaking, and other communicative skills, affecting a large number of children, its mechanisms of progression are not fully understood, and only one metabolomics study was found. In a study comparing autism regression with other non-autistic disorders, an untargeted and targeted UPLC–Q–Orbitrap CSF analysis revealed decreased β-hydroxybutyrate (subsequently confirmed by targeted analysis) and altered sphingolipids in patients with autism regression [69]. Although this review did not focus on any specific neurological disorders, the lack of other studies on autism raised questions. One recently published review on CSF composition in this disorder was of interest [70]. An analysis of the studies within it, which focused on metabolic composition, revealed that study [69] was the only one to use untargeted CSF metabolic profiling. However, several studies using targeted CSF analysis in autism focused on concentrations of 5-methyltetrahydrofolate and monoamine neurotransmitters, which will be discussed in Section 3.

Depression is a mental disorder affecting hundreds of millions of people worldwide, and animal models are used to discover its pathogenesis. In a CSF metabolomics study on a naturally occurring depressive model in macaques using GC–MS, 37 metabolites, mostly organic acids, alcohols, and carbohydrates, distinguished the target group from healthy controls [43]. CSF metabolomics study using GC–MS on postpartum depression revealed that from 51 metabolites, four organic acids (capric, dodecanoic, arachidic, and behenic acids) were negatively correlated, and l-tryptophan positively correlated with postpartum depression symptoms [71]. In cases of treatment-resistant depression, repetitive transcranial stimulation is used as a minimally invasive, potentially effective treatment, and CSF metabolomics, based on levels of 72 metabolites detected via LC–MS before and after 6-week treatment, demonstrated increased niacinamide, kynurenine, and creatinine, with decreased cysteine [72]. Since the studies described have different models and causes for the development of depression, generalizing the results is inappropriate.

### 2.5. Neurodegenerative Diseases

#### 2.5.1. Multiple Sclerosis

Multiple sclerosis is an incurable and difficult-to-diagnose disease of unknown cause in which the immune system mistakenly attacks myelin, leading to disruption of nerve signal transmission, plaque formation, and sensory and motor impairment. Multiple sclerosis has different subtypes: the initial clinical isolated syndrome, primary progressive multiple sclerosis, which worsens continuously from the start, relapsing-remitting multiple sclerosis, characterized by relapses and remissions, and secondary progressive multiple sclerosis with gradual worsening. Currently, routine CSF analysis in multiple sclerosis focuses on the detection of oligoclonal bands, elevated immunoglobulin G index, and, in some cases, neurofilament light chain as a marker of neuroaxonal damage [73]. However, these markers lack specificity for disease subtype and do not fully reflect metabolic changes underlying demyelination and neuroinflammation. Recent research has increasingly emphasized metabolomics for better understanding the mechanisms and differences among multiple sclerosis subtypes, leading to numerous CSF metabolomics studies [74]. In an animal model of multiple sclerosis study [75], described also in a previous study [41], researchers analyzed CSF from rats at disease onset (day 10) and peak (day 14) using untargeted GC–MS and targeted but not quantitative UPLC–QQQ to identify metabolic changes, finding decreased levels of certain amino acids at onset and increased levels of others, like glutamine and putrescine, at peak, reflecting shifts in energy, nitric oxide, and antioxidant pathways [75]. In human studies, combined targeted, but not quantitative, and untargeted UPLC–Q–TOF and GC–MS approaches were used to examine CSF metabolomics and lipidomics in patients with various subtypes of multiple sclerosis and controls with non-inflammatory conditions. They identified metabolites and lipids that differentiated these groups; for example, increased levels of some amino acids and metabolites associated with oxidative stress and energy metabolism in patients with multiple sclerosis, alongside reduced levels of others, such as tyrosine and hippuric acid [76]. Another targeted, but not quantitative, investigation focused on lipid, acylcarnitine, and amino acid CSF profiles using MALDI–TOF and HPLC–QQQ and found elevated glutamate levels in patients with relapsing-remitting multiple sclerosis compared with controls [77]. A study comparing CSF from patients with different multiple sclerosis subtypes and controls using HPLC–Q–Orbitrap identified 21 tryptophan and pyrimidine metabolites among 117 metabolites, which could help in disease subtype classification [78]. The same authors subsequently conducted a targeted CSF metabolomics study in secondary progressive multiple sclerosis using HPLC–QQQ and commercial kits that measure over 400 metabolites, including glycerophospholipids, acylcarnitines, glycerides, sphingolipids, biogenic amines, amino acids, cholesteryl esters, and total hexoses. Of the 200 detected metabolites, 35 were quantified, including glycine, asymmetric dimethylarginine, and glycerophospholipid PC–O (34:0), which were increased in the secondary progressive multiple sclerosis group compared with controls [79]. Nontargeted NMR and GC–MS CSF analyses of patients with relapsing-remitting and primary progressive multiple sclerosis did not distinguish disease stages, whereas targeted, quantitative flow injection and LC–QQQ analyses using commercial kits yielded a panel of lipid and amino acid metabolites that successfully separated groups [80]. A study on untargeted HPLC–Q–TOF metabolite detection identified significant differences in 12 amino acids and fatty acids among 60 metabolites in CSF from patients with early multiple sclerosis compared with controls [81]. Differentiating multiple sclerosis from similar disorders such as neuromyelitis optica and transverse myelitis, a metabolomics study analyzed CSF from patients with these three diseases and controls using GC–TOF; it identified seven key metabolites (elevated 1-monopalmitin, 1-monostearin, and glycolic acid with decreased glycine, inosine, threose, and butane-2,3-diol) from a total of 85 that varied significantly, and, together with clinical parameters, enabled disease distinction with high accuracy [9].

Since multiple sclerosis is a complex disease with various stages and manifestations, metabolic profiling of blood, CSF, and even urine is actively used for diagnosis, prognosis, and treatment. In particular, several review articles describe advances in metabolomics for multiple sclerosis [74,82,83]. In a 2021 review [74], which included some of the studies described [76,77,78,80], a complex analysis of metabolic pathways in blood, CSF, and urine was proposed. In particular, in CSF, 86 metabolites were identified, reflecting key biological processes that align with the metabolic changes associated with neuronal degeneration commonly seen in multiple sclerosis. Pathways related to small-molecule biochemistry are activated, characterized by increased uptake of glutamine and other amino acids, elevated levels of neurotoxic glutamate and triacylglycerols, and the release of eicosanoids and reactive oxygen species. There is a suppression of nucleotide synthesis, neurotransmitter release, and lipid peroxidation. Additionally, mechanisms involved in molecular transport, lipid metabolism, and amino acid metabolism show heightened activity.

#### 2.5.2. Alzheimer’s Disease

Alzheimer’s disease is a neurodegenerative disorder and a common form of dementia, similar to multiple sclerosis in being currently incurable, with challenging early diagnosis, unknown cause, and complex pathology. Established CSF biomarkers for Alzheimer’s disease include amyloid-β (Aβ42), total tau (t-tau), and phosphorylated tau (*p*-tau), which reflect amyloid plaque deposition and neurofibrillary tangle pathology [82]. Still, their levels in CSF are limited for early detection, diagnosing mild cognitive impairment, or assessing treatment efficacy, explaining the interest in CSF metabolomics studies. A CSF metabolomics analysis on Alzheimer’s patients with various severity forms and controls using GC–MS and untargeted and targeted, but not quantitative, LC–MS/MS resulted in over 340 metabolites and revealed elevated cortisol associated with more severe disease progression. A combination of elevated cysteine and decreased uridine could distinguish light Alzheimer’s with over 75% accuracy [84]. Metabolic profiling of CSF from individuals with mild cognitive impairment and cognitively normal controls using targeted, quantitative, but not validated, HILIC–HPLC–QQQ analysis identified 99 metabolites, including increased 3-hydroxybutyrate following triglyceride infusion, reflecting brain hypometabolism and the potential of ketogenic diets [85].

β-Amyloid derives from the transmembrane amyloid precursor protein, and CSF lipidomics is a promising field for potential biomarker search; thus, some studies will be mentioned. A GC–MS/MS study was performed on patients with Alzheimer’s disease and patients with mild cognitive impairment compared to age-matched healthy controls according to the aB&D method with pentafluorobenzyl bromide and *NN*-diisopropylethylamine derivatization. Among 20 metabolites, including polyunsaturated, monounsaturated, and saturated fatty acids, docosahexaenoic acid was lower in the Alzheimer’s group than in healthy controls. In contrast, alpha-linolenic acid was lower in the mild cognitive impairment group than in healthy controls [86,87]. An untargeted UPLC–Q–Orbitrap lipidomics study with a similar design compared patients with Alzheimer’s disease, patients with mild cognitive impairment, and age-matched controls, and applied aB&D lipid extraction. Almost 259 lipids were measured, including triacylglycerols, phosphatidylcholines, phosphatidylethanolamines, sphingomyelins, phosphatidylinositols, monohexosylceramides, cholesteryl esters, lysophosphatidylcholines, lysophosphatidylethanolamines, diacylglycerols, and ceramides. The content of specific lipids differed in the Alzheimer’s disease group. Combinations of 16:0/18:3-phosphatidylcholine and 18:0/18:1-phosphatidylinositol, as well as 16:0/18:3-phosphatidylcholine and d18:2/24:1-monohexosylceramide, were effective at distinguishing patients with Alzheimer’s disease from controls [88]. A UPLC–Q–TOF lipidomics study on Alzheimer’s disease patients with obstructive sleep apnea compared to patients without such a complication was conducted on CSF samples obtained the following morning using extraction with methyl-*tert*-butyl ether. Over 200 lipids were detected, and from 11 dysregulated lipids, an oxidized ceramide (OxCer(40:6)) and an oxidized triglyceride (OxTG(57:2)) distinguished the observed groups [89].

#### 2.5.3. Parkinson’s Disease

Parkinson’s disease is the second most common neurodegenerative condition, involving progressive loss of dopamine-producing neurons in the midbrain that control voluntary movements, with unclear causes. Diagnosis relies mainly on clinical evaluation of motor signs, and current treatments such as levodopa and dopamine agonists aim to mitigate symptoms but provide limited relief since the disease is incurable. Candidate markers under investigation include α-synuclein, DJ-1, and lysosomal enzymes, but their diagnostic accuracy remains limited [90]. Two metabolomics studies on untargeted CSF GC–MS and UPLC–MS/MS analyses have explored biochemical changes associated with Parkinson’s disease. The first study investigated postmortem CSF from affected individuals and controls and identified 19 metabolites from a total of 192 that differed significantly, including those from the kynurenine and glutathione metabolic pathways, reflecting mitochondrial dysfunction and oxidative stress [91]. The second study tracked early-stage, untreated patients over two years, identifying almost 600 specific plasma and almost 400 CSF metabolites, with CSF homovanillic acid (HVA) showing little change, while 15 plasma metabolites—particularly fatty acids and compounds like xanthine—correlated with disease progression [92]. Another CSF and serum metabolomics study using targeted HPLC–QQQ and commercial kits that measure almost 200 metabolites, including glycerophospholipids, acylcarnitines, sphingolipids, biogenic amines, amino acids, and total hexoses, yielded 50 metabolites in CSF and almost 150 in serum. Subsequent comparison of patients with Parkinson’s disease and atypical Parkinsonian disorders revealed altered tyrosine, *trans*-4-hydroxyproline, putrescine, and total dimethylarginine in both CSF and serum [93]. A CSF metabolomics study [94], based on MWAS data [64], identified nine metabolites associated with Parkinson’s disease, including ribitol, lysine, and *O*-sulfo-l-tyrosine, which increased significantly in Parkinson’s disease risk.

A useful review article on metabolic profiling for distinguishing between Parkinson’s disease and multiple system atrophy, which share similar symptoms in the early stages, includes several studies that used not only MS-based methods but also targeted and untargeted metabolomics. The paper presents metabolic pathways characteristic of each pathology compared to controls; however, due to the small number of metabolites, no significant differences between the two pathologies were identified, indicating the need for further research in this area [95].

#### 2.5.4. Amyotrophic Lateral Sclerosis

Amyotrophic lateral sclerosis is an uncommon, incurable, occasionally genetic, but inevitably fatal neurodegenerative disease with primary lateral sclerosis, affecting only upper motor neurons, and progressive muscular atrophy, involving only lower motor neurons. Most cases are sporadic, but approximately 10% are familial, with mutations in the superoxide dismutase-1 gene being among the most studied. Currently, no established CSF biomarkers are routinely used for diagnosis or progression monitoring; candidate markers such as neurofilament light chain and phosphorylated neurofilament heavy chain have shown promise but are not yet part of standard clinical practice [96]. One GC–TOF metabolomic study followed the recommendations of the Metabolomics Standards Initiative. It analyzed CSF samples from sporadic and familial cases of amyotrophic lateral sclerosis, including those with superoxide dismutase-1 gene mutations. The study identified 120 peaks corresponding to various acids; 40 were identified, revealing variability in CSF metabolites among subtypes, with glutamate and glutamine lower in the familial amyotrophic lateral sclerosis group. Notably, those without gene mutations showed less heterogeneity than the sporadic group, with the gene mutation group forming a separate, homogeneous cohort [97]. Another metabolomics study used UPLC–Q–TOF to analyze CSF samples from patients with amyotrophic lateral sclerosis and from controls with other neurological disorders. Although specific compounds were not listed, four unknown metabolite signatures predicted amyotrophic lateral sclerosis with over 80% accuracy [98].

### 2.6. Epilepsy

Epilepsy is a chronic neurological disorder characterized by the body’s predisposition to sudden seizures caused by abnormal, excessive, or insufficient inhibition in the nervous system. Despite often effective treatment, leading to complete seizure remission, no CSF biomarkers are currently used in routine clinical practice for epilepsy diagnosis or treatment monitoring and metabolomics research is essential for identifying new therapeutic targets and monitoring treatment responses. Tandem GC–MS/MS CSF analysis in children with and without epilepsy revealed several metabolite differences across age groups. Children younger than 5 years showed reduced 2-ketoglutaric acid levels and increased pyridoxamine and tyrosine levels. Valproic acid treatment was associated with elevations in amino acids and related compounds, suggesting that disruptions in vitamin B6 metabolism and energy production may contribute to the onset of epilepsy. In older children (6–17 years), decreased 1,5-anhydroglucitol was observed in epileptic patients, with certain antiepileptic drugs influencing this metabolite level [99]. GC–MS analysis of CSF metabolomics in epileptic and non-epileptic patients identified metabolic differences in 56 metabolites, including decreased α-ketoisocaproic acid and xylose and increased glycine in epileptic individuals [100]. Metabolomics study using UPLC–Q–TOF identified potential biomarkers in CSF that differentiate outcomes in pediatric patients with status epilepticus (a prolonged and severe seizure form potentially leading to mortality). Alterations in the levels of 16 metabolites were noted in patients with a good outcome compared to controls. In contrast, 23 metabolites were altered in patients with a poor outcome compared with controls. A multivariate model combining glutamyl–glutamine and 3-iodothyronamine accurately predicted adverse outcomes, suggesting involvement of the tricarboxylic acid cycle and arginine biosynthesis pathways in these effects [101]. Collectively, the analyzed studies indicate that CSF metabolic alterations in epilepsy comprise a disruption of neurotransmitter balance and related amino acids, reflecting a shift toward neuronal hyperexcitability; impairments in energy metabolism, including mitochondrial dysfunction and alterations in tricarboxylic acid cycle intermediates; neuroinflammatory, involving tryptophan–kynurenine metabolism; and oxidative stress, including alterations in glutathione metabolism.

### 2.7. Oncological Diseases

Oncological diseases of interest from a CSF metabolomics perspective include brain, hematopoietic, and lymphoid tissue tumors. Brain tumors arise from the abnormal transformation of healthy neural (medulloblastomas in children) or glial cells (gliomas), leading to uncontrolled growth. Accurate early diagnosis is vital, yet challenging, since magnetic resonance imaging often cannot detect early tissue changes, and biopsies are difficult for deep-seated tumors. An untargeted and targeted (quantified but not validated) GC–MS metabolomics study examined CSF from glioma patients across four different grades, revealing 60 metabolites (amino acid, tricarboxylic acid cycle, and glycolysis metabolites), including citric and isocitric acids, elevated in glioblastoma, with higher lactic acid linked to worse outcomes [102]. A targeted, but not quantitative, HILIC–UPLC–Q–TOF metabolomics approach measured over 124 metabolites in glioma patients, finding alterations in 38 metabolites with increased amino acids and nucleotides in malignant cases, while certain metabolites decreased. Notably, newly diagnosed patients demonstrated an isolated cohort with lower tryptophan metabolite levels [103]. In pediatric medulloblastoma, combined transcriptomic, metabolomic, and lipidomic UPLC–Q–Orbitrap analyses identified more than 1000 metabolites and lipids, with altered tricarboxylic acid cycle intermediates and triglycerides in patients of the target group compared with controls, suggesting tumor hypoxia [104].

Tumors of the hematopoietic and lymphoid tissues, which can affect the blood, lymphatic system, and bone marrow, include leukemias, lymphomas, and myelomas. In two studies on pediatric lymphoblastic leukemia, CSF metabolomics using GC–MS and RP and HILIC–HPLC–Q–TOF identified 313 metabolites with eight metabolites associated with fatigue in the discovery cohort and 409 metabolites with three metabolites (gamma-glutamylglutamine, dimethylglycine, and asparagine) associated with fatigue in the replication cohort, reflecting neurotransmitter and glutathione recycling alterations [105]. Subsequently, a study on B-cell acute lymphoblastic leukemia patients indicated that plasma (816 metabolites) samples could reliably mirror bone marrow (774 metabolites) and CSF (366 metabolites) metabolite profiles with pyruvate and asparagine positively correlated between plasma and bone marrow; dimethylglycine positively and gamma-glutamylglutamine negatively correlated between plasma and CSF, offering a less invasive plasma analysis to assess treatment response [106]. A CSF metabolomics study using LC–Q–TOF analysis aimed to differentiate patients with various tumor types: patients with primary and secondary CNS lymphomas, as well as lung cancer with or without brain metastases, identifying 27 of 508 metabolites that distinguished groups based on amino acid and citrate metabolism [107]. In a subsequent study on primary CNS lymphoma compared to other tumors and controls of the same authors, the exact CSF metabolome results using flow injection and LC–MS/MS analysis were not provided; only metabolic pathways were mentioned to distinguish patient groups, including porphyrin, glutathione, amino, and fatty acid metabolisms [108].

### 2.8. Neuroinflammatory Diseases

Neuroinflammatory diseases without timely and correct diagnosis and treatment may lead to irreversible neurological changes, disability, and death. However, no specific blood biomarkers have been identified to date, and nonspecific clinical symptoms, as in many non-CNS diseases, complicate diagnosis. CSF culture, cell count, protein and glucose levels are widely used to diagnose neuroinflammatory diseases; however, their specificity is moderate. Nevertheless, CSF collection for neuroinflammatory disease diagnosis is a routine practice, and the use of CSF residues for scientific purposes accounts for a large number of metabolomics studies. Neuroinflammatory diseases can be mainly divided into viral and bacterial, as well as primary and secondary. In a CSF metabolomics study comparing neurosyphilis with non-neurosyphilis and syphilis-free patients, a UPLC–Q–TOF analysis detected 1800 metabolites, with l-gulono-gamma-lactone, d-mannose, *N*-acetyl-l-tyrosine, and hypoxanthine separating all groups [109]. In another CSF metabolomics study on neurosyphilis patients compared to non-neurosyphilis ones using LC–Orbitrap, six metabolites (alpha-kamlolenic acid, l-histidine, bilirubin, prostaglandin E2, palmitoyl-l-carnitine, and butyryl-l-carnitine) were proposed as biomarkers of neurosyphilis [110]. Three studies within the same scientific group on various viral CNS infections used commercial kits for CSF–targeted, quantitative, but unvalidated, HPLC–QQQ analysis, yielding 188 metabolites: 21 amino acids, 21 biogenic amines, 91 glycerophospholipids, 40 acylcarnitines, 15 sphingolipids, and the sum of hexoses. The first study focused on three manifestations of varicella zoster virus reactivation (segmental zoster, facial nerve zoster, and zoster meningitis and/or encephalitis), compared to enteroviral meningitis, idiopathic Bell’s palsy, and normal pressure hydrocephalus. Around 90 metabolites distinguished all three forms of varicella zoster virus reactivation from other samples, with four metabolites, including glycine, associated with meningoencephalitis [111]. CSF samples from patients with enteroviral meningitis were subsequently studied, including samples with elevated and normal CSF leukocyte counts compared to non-inflamed patients with idiopathic Bell’s palsy and normal pressure hydrocephalus. Four metabolites, including asparagine, glycine, and especially kynurenine, were the best biomarkers for enteroviral meningitis [112]. A further targeted (quantified but not fully validated) study (for kynurenine and tryptophan) on various CNS diseases, including bacterial and viral meningitis/encephalitis, neuroborreliosis, autoimmune neuroinflammation (including multiple sclerosis), and noninflamed controls, identified kynurenine as a biomarker of bacterial and viral CNS infections. Moreover, tryptophan was lower in viral infections and neuroborreliosis [113]. Another scientific group, focusing on CSF neuroinflammation, firstly conducted an untargeted metabolomics study using UPLC–Q–Orbitrap on patients with acute encephalitis compared to non-inflammatory neurological disease controls and found 35 metabolites from tryptophan–kynurenine pathway (higher kynurenine/tryptophan ratio, anthranilic acid/3-hydroxyanthranilic acid ratio, kynurenine, quinolinic, and anthranilic acids with lower tryptophan, 3-hydroxyanthranilic, and kynurenic acids in encephalitis group) and nitric oxide pathway (higher asymmetric dimethylarginine and argininosuccinic acid and asymmetric dimethylarginine/arginine ratio with lower arginine and citrulline in encephalitis group), and neopterin separating two groups [114]. Subsequently, the authors summarized studies on neuroinflammation in a review that will be discussed at the end of the current section [83], validated an UPLC–QQQ protocol for the quantification of 13 main metabolites from tryptophan–kynurenine and nitric oxide pathways in CSF [115], and conducted clinical studies on the diagnostic assessment of these metabolites in epilepsy and neuroinflammatory diseases [116,117], which will be discussed in Section 3.2 in more detail. These two groups of sequential studies demonstrate how global metabolomics can lead to the discovery of very promising, clinically significant biomarkers.

Meningitis is an inflammation of the meninges that protect the brain and spinal cord; its primary form, caused by *Neisseria meningitides* (i.e., meningococcal meningitis), is usually diagnosed successfully by microbiological or PCR analysis, whereas the secondary form develops as a complication of the main disease [6,118]. CSF metabolomics of the tuberculous meningitis, caused by *Mycobacterium tuberculosis,* compared to viral, bacterial, and cryptococcal meningitides, was conducted via UPLC–Q–TOF in positive and negative ESI modes. It resulted in almost 1700 metabolic features: 13 metabolites were separated between tuberculous and viral meningitis, 16 between tuberculous and bacterial meningitis, and 9 between tuberculous and cryptococcal meningitis, with most metabolites increased in tuberculous meningitis [119]. A group of scientists conducted two studies on CSF samples from a large group of patients with tuberculous meningitis (more than 1000 participants). The first study, which was targeted, but not validated, for the tryptophan metabolite UPLC–QQQ analysis, compared patients with tuberculous meningitis with patients with bacterial and cryptococcal meningitis, and non-infectious controls, and revealed a moderate association of tryptophan with 60-day mortality [120]; however, in the second global untargeted study on tuberculous meningitis mortality using HPLC–Q–Orbitrap, over 600 metabolites were detected, with 10 of them associated with 60-day mortality: 4-hydroxyphenylacetic acid, phenyllactic acid, and some hydroxylated fatty acids in addition to the previously discovered tryptophan [121]. An amino acid-targeted, non-validated GC–MS analysis of CSF samples from children with tuberculous meningitis compared with meningitis-negative controls revealed elevated levels of asparagine, alanine, lysine, glycine, and proline among the 22 amino acids [122]. A CSF metabolomics study using LC–MS/MS and GC–MS on bacterial meningitis in infants caused by *Staphylococcus aureus* or *Streptococcus agalactiae* compared to those without bacterial meningitis resulted in more than 400 metabolites, with six metabolites separating the two groups [123]. In a study on neonatal sepsis with meningoencephalitis, CSF and serum metabolomics studies were conducted via HPLC–Q–Orbitrap and found 13 from 91 metabolites to have significant differences in the CSF between the two groups (increased pyrrolidine, pyridoxal, l-proline, dopamine, HVA, kynurenic, pyruvic, phenolglyoxylic, and glycocholic acids with decreased homo-l-arginine, creatinine, urea, and phosphoric acid in the meningoencephalitis group) [124].

Initially considered a respiratory tract infection, COVID-19 has demonstrated its ability to penetrate the CNS. This explains the interest in analyzing CSF from patients with signs of CNS disorders caused by COVID-19 (neuro-COVID), as well as comparisons with other CNS infections, such as various neurotropic viruses. Widely targeted study using commercial kit for FIA-MS/MS of more than 600 CSF metabolites in patients with neuro-COVID in acute and post-COVID-19 stage, viral meningitis, encephalitis, or myelitis caused by herpes simplex virus, varicella zoster virus, enterovirus and tick-borne encephalitis virus, and aseptic neuroinflammation of unknown etiology revealed that citrulline, ceramide (d18:1/18:0), and methionine were elevated with lower triglyceride TG(20:1_32:3) in neuro-COVID compared to other groups [125]. Neurologic post-acute sequelae of COVID-19 infection, which include headache, ageusia, anosmia, and stroke, can persist for up to 3 months after onset. A CSF metabolomics study was conducted in patients with such symptoms, comparing them with the neuroinflammatory group and controls, using UPLC-Orbitrap. Among 93 metabolites identified, almost 60 were higher and 10 lower in the target group than in controls [126].

There are also some review articles summarizing the application of CSF metabolomics and lipidomics for the detection of CNS neuroinflammation in general [83], for viral and bacterial CNS infections [127], or for meningitis in particular [128]. The first review, which also analyzed various studies from the current review, pointed out that CNS neuroinflammation occurs not only during meningitis, encephalitis, and other CNS infections, but also in multiple sclerosis, Alzheimer’s disease, and depression. The main pathways listed in neuroinflammation included the tryptophan–kynurenine pathway, with elevated kynurenine or kynurenic acid, decreased tryptophan, and an elevated kynurenine/tryptophan ratio; and lipids, resulting in alterations in ceramide, sphingolipids, phospholipids, and oxylipins [83]. The second review focused on LC–MS-based metabolomics and lipidomics studies of various CNS infections, including most of the studies from the current section. In summary, phospholipids, carnitine, and tryptophan are the most promising biomarkers for bacterial and viral infections [127]. The third review, aimed at differential diagnosis of meningitis, noted that the shift towards anaerobic glycolysis during bacterial meningitis involves alterations in energy and tricarboxylic acid cycle metabolism [128].

### 2.9. “Secondary” and Undiagnosed Conditions Affecting the Nervous System

Some other CNS conditions, which are secondary to a primary diagnosis, used CSF metabolomics approaches. Such a study used LC–MS/MS to assess hepatic encephalopathy, a neurological complication of liver disease and/or a portosystemic shunt, and identified altered levels of 73 metabolites (amino acids, bile acids, acylcarnitines, and nucleosides) compared with controls [129]. A longitudinal CSF and metabolomics study via UPLC–Q–TOF on diabetic kidney disease, which is an end-stage renal disease in type 2 diabetes mellitus, started in 2017 (current presence of the disease and controls) and in 2021 (new-onset disease), proposed increased CSF tryptophan in new-onset disease compared to controls with altered uric acid and paraxanthine in the current disease group [130]. LC–MS/MS and GC–MS were applied in a CSF untargeted metabolomics study on human immunodeficiency virus (HIV)-associated neurocognitive disorders, HIV-positive patients, and HIV-negative controls, both including an almost equal amount of young and older participants. Of the 200 metabolites, more than 100 were identified, including amino acids, carbohydrates, lipids, and nucleotides. Alterations in glutamate, N-acetylaspartate, myo-inositol, beta-hydroxybutyric acid, and 1,2-propanediol, which correlated with worse neurocognitive test scores and plasma inflammatory biomarkers, reflect changes in neurotransmitter production, oxidative stress, mitochondrial function, and metabolic waste. During age-related analysis, metabolites, which were altered in HIV-positive patients, overlapped with those altered in older HIV-negative controls, indicating a pattern of accelerated aging [131].

Patients with undiagnosed diseases represent a special group for whom metabolomic analysis can provide additional information. One lipidomics and metabolomics study of blood, urine, and CSF using GC–MS and UPLC–Q–Orbitrap was conducted in the United States with this patient group, and its results are publicly available. This analysis resulted in 82 CSF metabolites, 81 polar plasma metabolites, and 116 urine metabolites [132].

### 2.10. Key Metabolic Pathways Altered in CSF Across Neurological Disorders

Analysis of the untargeted CSF metabolomics studies reviewed in Section 2 reveals several metabolic pathways that are repeatedly dysregulated across different neurological conditions, including neuroinflammation, neurodegeneration, epilepsy, and CNS infections. Below, the most consistently altered pathways are briefly described, with an emphasis on the direction of metabolite changes (increased or decreased).

#### 2.10.1. Tryptophan–Kynurenine Pathway

The tryptophan–kynurenine pathway is a major route of tryptophan metabolism that is activated by pro-inflammatory cytokines (interferon-γ, tumor necrosis factor-α) via the enzyme indoleamine-2,3-dioxygenase or tryptophan-2,3-dioxygenase [83]. Across multiple CSF studies on neuroinflammatory conditions (encephalitis, meningitis, multiple sclerosis), neurodegenerative diseases (Alzheimer’s disease, Parkinson’s disease), and even aging, several consistent alterations have been observed.

Consistently increased metabolites: kynurenine, kynurenine/tryptophan ratio, quinolinic acid, anthranilic acid, and neopterin are elevated in patients with acute neuroinflammation, bacterial and viral CNS infections, and multiple sclerosis compared to controls [78,112,114,115,117]. Elevated quinolinic acid is particularly associated with neurotoxicity and disease severity in infectious and inflammatory conditions.

Consistently decreased metabolites: tryptophan itself is often decreased in inflammatory states due to indoleamine-2,3-dioxygenase activation [113,114,121]. Kynurenic acid, which has neuroprotective properties, is decreased in epileptic spasms and some neuroinflammatory conditions [116,117]. 3-Hydroxyanthranilic acid levels may also be reduced in acute encephalitis [114].

Notably, metabolite ratios are often more informative than absolute concentrations. The kynurenine/tryptophan ratio and the quinolinic acid/kynurenic acid ratio are consistently elevated in neuroinflammation across multiple studies [83,114,115].

#### 2.10.2. Lipid Metabolism

Lipid metabolism disturbances are consistently reported in CSF metabolomics studies of multiple sclerosis, Alzheimer’s disease, and other neurodegenerative conditions. These alterations reflect demyelination, membrane turnover, oxidative stress, and neuroinflammation.

Consistently increased lipids: in multiple sclerosis, elevated levels of ceramides, sphingomyelins, glycerophospholipids (e.g., PC–O (34:0)), and triacylglycerols have been reported [61,76,79,80], indicating active myelin breakdown and membrane remodeling. In Alzheimer’s disease, increases in specific phosphatidylcholines (e.g., 16:0/18:3-PC) and monohexosylceramides have been observed [88]. Oxidized ceramides and triglycerides are elevated in Alzheimer’s patients with obstructive sleep apnea [89].

Consistently decreased lipids: decreases in docosahexaenoic acid have been reported in Alzheimer’s disease [87], and reductions in alpha-linolenic acid are observed in mild cognitive impairment [86]. In multiple sclerosis, decreases in certain phosphatidylethanolamines and hexosylceramides have also been noted [61].

#### 2.10.3. Energy Metabolism

Alterations in energy metabolism are commonly observed across neurological disorders, reflecting mitochondrial dysfunction and shifts in brain energy substrate utilization.

Consistently increased metabolites: citric and isocitric acids are elevated in glioblastoma [102] and medulloblastoma [104]. β-Hydroxybutyrate is decreased in autism regression [69] but may increase following ketogenic dietary interventions in mild cognitive impairment [85].

Consistently decreased metabolites: tricarboxylic acid cycle intermediates (e.g., 2-ketoglutaric acid, succinate, fumarate) are decreased in pediatric epilepsy [99] and in some neurodegenerative conditions [91,104], suggesting impaired mitochondrial function. 1,5-Anhydroglucitol, a marker of glucose metabolism, is decreased in epilepsy patients on certain antiepileptic medications [99].

### 2.11. Conclusions to Untargeted Metabolomics

The section on untargeted CSF metabolomics included more than 60 studies, a brief description of which is provided in Table 1. Analysis of these studies demonstrates that the level of detail in reporting analytical methodologies and metabolomic results varies substantially. This variability is likely due to the predominantly clinical rather than analytical focus of most studies. Typically, the most comprehensively described sections are those related to patient cohort characteristics and statistical processing of multivariate data. In many studies, complete lists of identified metabolites are not provided, and only compounds that differ significantly between the compared groups are reported.

The vast majority of studies used LC–MS, whereas GC–MS was used less frequently, and combined analytical approaches were used only occasionally. In most studies reporting sample preparation conditions, the CSF volume did not exceed 200 µL. Sample preparation for LC–MS generally includes protein precipitation, followed by methanol extraction, solvent evaporation, and reconstitution in an appropriate organic solvent; however, the optimal conditions described in [37] are not commonly used. In GC–MS, the final step involved classical two-step derivatization (oximation and silylation). Thus, despite differences in analytical workflows, the overall complexity of sample preparation is comparable between the two methods. At the same time, although approaches to standardizing untargeted analysis have been proposed in the literature, most CSF metabolomics studies do not adhere to standardized protocols, which complicates cross-study comparisons and is a key factor limiting reproducibility.

**Figure 3:**
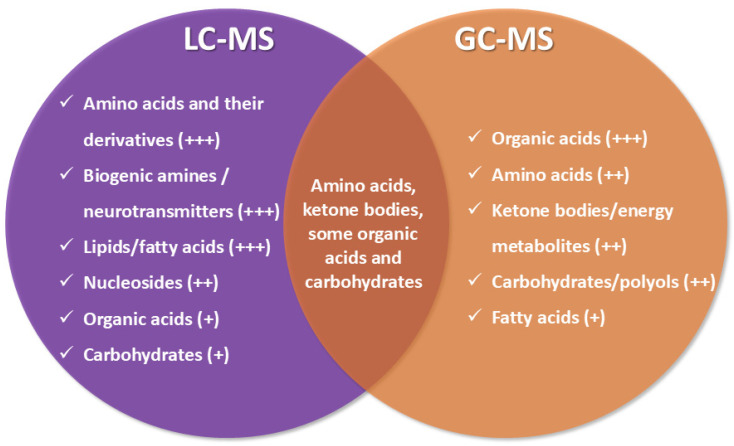
Relative coverage of major metabolite classes in untargeted CSF metabolomics studies using LC and GC–MS-based methods. +++—frequent: >50% of studies; ++—moderate: 20–50%; +—limited: 5–20%.

The number of detected compounds ranges from tens to more than a thousand. Relative coverage of major metabolite classes in untargeted CSF metabolomics studies is demonstrated in Figure 3. LC–MS demonstrates superior coverage for polar nitrogen-containing compounds (amines, amino acids) and lipids, while GC–MS provides complementary advantages for organic acids, short-chain energy metabolites, and carbohydrates. The overlapping region represents metabolite classes routinely detected by both platforms.

In all studies involving group comparisons, statistically significant differences were observed for at least some metabolites, as expected given the high dimensionality of the data and the associated multiple-comparison issue. Therefore, statistically significant differences do not necessarily imply biological relevance and require cautious interpretation; however, when appropriate statistical approaches are applied, they may reflect genuine metabolic alterations. Additionally, some studies apply filtering based on metabolite occurrence across samples to reduce the impact of artefactual peaks. However, this approach carries the risk of excluding potentially important biomarkers that are present predominantly or exclusively in one of the studied groups.

It should also be noted that untargeted metabolomics is, in most cases, semi-quantitative, which limits the direct comparability of metabolite concentrations across studies.

Despite the use of different analytical platforms, the identified metabolites predominantly belong to a limited number of metabolic pathways, including energy metabolism, oxidative stress, amino acid metabolism (particularly tryptophan metabolism), and lipid metabolism. These pathways correspond to fundamental metabolic processes characteristic of CSF. Accordingly, alterations at the pathway level are generally nonspecific and may reflect a common CNS response to pathological processes. At the same time, individual metabolites may exhibit higher diagnostic specificity; however, such candidates often fail to be validated in independent cohorts, showing high sensitivity but limited specificity. It is worth noting several studies that compared a large number of sample groups and identified specific metabolites. However, the sample size for each specific group was very small (less than 20 patients) [108,111]. This highlights the need to shift from identifying single biomarkers to analyzing multidimensional metabolic patterns.

A combination of CSF biological characteristics and analytical constraints of MS-based methods likely drives the recurrence of specific metabolic pathways across studies. Under untargeted conditions, compounds with relatively high concentrations and favorable ionization properties (particularly in LC–MS analysis) are preferentially detected, resulting in a systematic bias toward analytically accessible metabolites. Thus, in practice, not the entire metabolome is assessed, but rather its measurable fraction, which should be taken into account when interpreting results.

A logical question arises: does the frequent detection of disturbances in metabolic pathways such as the tryptophan–kynurenine pathway and lipid metabolism during metabolic profiling of CSF (using GC– and HPLC–MS-based methods) reflect a true biological convergence of pathological mechanisms in the CNS, or is it a consequence of the analytical accessibility of these metabolites for these analytical methods? Although the influence of an analytical bias toward readily ionizable and extractable compounds cannot be completely ruled out, the totality of the data convincingly supports their biological significance. First, activation of the tryptophan–kynurenine pathway is directly induced by proinflammatory cytokines (interferon-γ, tumor necrosis factor-α) via the indoleamine-2,3-dioxygenase/tryptophan-2,3-dioxygenase enzymes, representing a universal response to neuroinflammation—a common link in the pathogenesis of virtually all CNS diseases [83]. Secondly, changes in the lipid profile objectively reflect fundamental processes of demyelination, membrane breakdown, and oxidative stress, which are inevitable, in particular, during neurodegeneration or infection [127]. Thus, although analytical methods are indeed targeted at this class of compounds, the identified convergence reflects not an artifact, but rather fundamental pathophysiological mechanisms—neuroinflammation and lipid-mediated degeneration—making these metabolic pathways valuable diagnostic targets, rather than simply highly detectable markers.

In all reviewed studies, pathway analysis was performed to interpret changes in metabolite levels. However, not all metabolites were functionally uninterpreted: compounds with limited or inconsistent representation in the literature often remain without biochemical explanation, reflecting current limitations in metabolite annotation and the incompleteness of existing biochemical databases.

The clinical design of the studies varies from comparisons between patients and healthy controls to comparisons among patients with different pathological conditions. The former approach is the most informative for identifying disease-associated metabolic patterns; however, its implementation is limited by ethical and methodological challenges in obtaining CSF from healthy individuals, making it the rarest and most valuable source of data on CSF biological variability. Therefore, control groups more often consist of patients undergoing lumbar puncture for clinical reasons. Such designs may involve comparisons with groups of different etiology (e.g., patients without CNS disorders) or with related conditions (e.g., different neurodegenerative or neuroinflammatory diseases). In the latter case, detecting differences is considerably more challenging; however, such comparative designs are more clinically relevant, as they allow assessment of the specificity of observed alterations in the context of differential diagnosis rather than merely their sensitivity. In clinical practice, CSF analysis is not always required for diagnosis; however, in certain cases it remains a critical tool for differential diagnosis, for example, in distinguishing Alzheimer’s disease from other forms of dementia, Parkinson’s disease from other movement disorders, and multiple sclerosis from other movement disorders, and multiple sclerosis from other demyelinating conditions.

A major limitation of most studies is the small sample size, largely due to the limited availability of CSF. In this context, large-scale studies with substantial numbers of patients within a single group [64,121] are particularly valuable.

In the future, improving the reproducibility and clinical relevance of untargeted CSF metabolomics may depend on both the standardization of analytical workflows and the integration of metabolomic data with other -omics data (e.g., proteomics and transcriptomics), thereby enabling a more comprehensive characterization of CNS pathophysiological processes. Some other challenges and emerging directions of untargeted metabolomics are described in a review [133], where the main limitation of untargeted metabolomics today was formulated not as data acquisition, but as its correct interpretation and identification of metabolites.

## 3. Targeted Metabolomics

**Figure 4:**
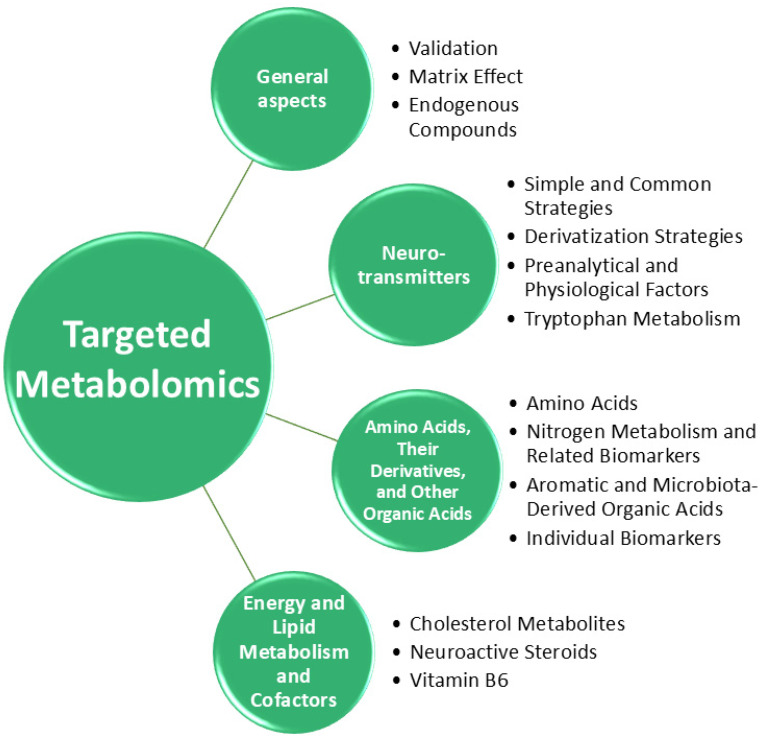
The main aspects covered in Section 3.

As mentioned in the Introduction, after metabolic profiling and the application of various statistical and bioinformatics approaches that yield a list of potential biomarkers, it is necessary to develop a selective and sensitive method for detecting a specific compound or a group of related compounds. This section will present methods for the determination of various classes of compounds using GC– and LC–MS-based approaches (Figure 4). For UPLC–MS/MS, both simple methods and more labor-intensive approaches with various extraction and derivatization methods will be presented. Methods for the quantitative determination of metabolites in this section are presented as unvalidated, partially validated, or fully validated. Since the most reliable results were obtained using partially or fully validated methods, they will be described in more detail, while unvalidated methods will be mentioned briefly.

### 3.1. General Aspects

#### 3.1.1. Validation

Validation of analytical methods for the quantitative assessment of potential biomarkers in biological matrices is critical to the success of clinical trials, as it ensures the reliability of the obtained data. Validation involves evaluating the following parameters: linearity, lower and upper limits of quantification, selectivity, specificity, sensitivity, matrix effect, accuracy, precision, recovery, and the various stabilities of the analyte and working solutions. Determining these parameters for full validation requires conducting a large number of experiments with a defined number of replicates and strict reproducibility to ensure the acceptability of the results, which results in a relatively small number of fully validated methods. Furthermore, full validation requires a large amount of biological matrix, and certain stages require an analyte-free matrix.

In the past, the FDA and EMA had their own regulatory expectations for bioanalytical method validation [27,28], but after 2023 the ICH M10 guideline [29] was issued which largely harmonized earlier recommendations and provided clearer definitions, thereby facilitating global standardization of bioanalytical method validation. Current documents define common validation principles and acceptance criteria, while having minor differences in experimental design and data interpretation compared to previous guidelines. A comparative summary of key validation parameters is presented in Table 2.

Overall, the requirements across the three guidelines are highly consistent, particularly regarding acceptance criteria for accuracy, precision, and calibration performance. The most notable differences relate to the evaluation of matrix effects, the use of signal-to-noise ratios for sensitivity assessment, and the level of detail provided for experimental procedures.

For CSF, additional constraints apply due to limited sample availability and its unique composition. Validation is often performed using pooled CSF or surrogate matrices, which must be carefully justified. In such cases, particular attention should be paid to matrix effects, selectivity, and sensitivity, as these parameters may differ significantly from those observed in plasma or serum.

#### 3.1.2. Matrix Effect

Matrix effects are alterations in the analytical signal caused by co-eluting endogenous or exogenous components of the biological matrix, leading to ion suppression or enhancement and thereby affecting the accuracy and precision of quantitative analysis [134]. Guidelines issued by FDA [27], EMA [28], and ICH M10 [29] recognize matrix effect as a critical parameter in bioanalytical method validation, especially for MS-based methods. Current requirements: matrix effects must be evaluated using at least 6 independent sources of matrix; assessment should be performed at low and high QC levels; variability of matrix effect (CV) should not exceed 15%; use of stable isotope-labeled internal standards is strongly recommended to compensate for matrix effects. ICH M10 harmonizes FDA and EMA approaches and explicitly requires the quantitative evaluation of the matrix factor.

The primary and most widely accepted approach is the matrix-free method. Two sets of samples are prepared: neat standard solution (matrix-free), and blank matrix extract spiked post-extraction. The necessary calculations are the following:

*Matrix factor* = *Response*(*post* − *extraction*)/*Response*(*neat*)

*Internal standard-normalized matrix factor* = *Matrix factor*(*analyte*)/*Matrix factor*(*internal standard*)

If the matrix factor is less than 1, there is an ion suppression; if the matrix factor is more than 1, there is an ion enhancement. Acceptance criteria are a CV of matrix factor (or internal standard-normalized matrix factor) less than 15% and consistent results across at least 6 different matrix sources.

Alternative approaches include post-column infusion, which involves continuous infusion of the analyte during injection of the matrix extract; standard addition, in which calibration is performed directly in each sample; matrix-matched calibration, in which calibration standards are prepared in the same matrix; and slope comparison. In the last approach, matrix effect is evaluated by comparing the slopes of calibration curves:

*Matrix effect* = (*Slope*(*matrix*)/*Slope*(*solvent*) − 1) × 100%

Generally, LC–MS-based methods are more frequently validated; however, the described approaches can be used to assess matrix effects in GC–MS-based methods. Unlike LC–MS-based methods, where ion suppression dominates, in GC–MS-based methods, matrix effects are often associated with signal enhancement, known as the matrix-induced response enhancement effect [135,136].

#### 3.1.3. Endogenous Compounds

Validation of bioanalytical methods for endogenous compounds poses specific challenges compared to exogenous analytes, primarily because of the absence of analyte-free biological matrices. Since endogenous compounds are inherently present in all study samples, preparing calibration standards and QC samples in a true blank matrix is not feasible.

According to ICH M10 [29], and also previous FDA [27], the general validation principles (accuracy, precision, selectivity, etc.) remain applicable to endogenous analytes. However, additional considerations are needed to address the lack of a blank matrix and ensure the method remains scientifically valid.

All guidelines emphasize that calibration standards should ideally be prepared in the same matrix as study samples. The absence of endogenous interference must be demonstrated, or its contribution must be appropriately taken into account. QC samples must reflect endogenous background levels when relevant. Any alternative approach (e.g., surrogate matrix) must be justified and validated. The FDA explicitly states that analyte-free matrices should be used whenever possible and must be demonstrated to be free of interference; however, when this is not feasible, alternative strategies are acceptable if properly justified. ICH M10 further harmonizes this concept by formally recognizing surrogate approaches and requiring demonstration of equivalence between surrogate and authentic matrices.

To overcome the absence of blank matrices, four main strategies are recognized (and implicitly accepted across guidelines) [26]:

1. Background subtraction: In this approach, endogenous concentrations in the matrix are measured and subtracted from spiked samples to construct calibration curves. However, LLOQ is limited by endogenous baseline levels rather than instrument sensitivity, and high variability in endogenous levels may reduce reproducibility.
2. Standard addition method: each study sample is spiked with increasing concentrations of analyte, and calibration is performed individually per sample. Eliminates matrix effect differences between samples. However, it is labor-intensive and sample-consuming.
3. Surrogate matrix approach: calibration standards are prepared in an artificial or modified matrix that does not contain the analyte. The key requirement here is to demonstrate the equivalence in matrix effect and recovery between surrogate and authentic matrices. Parallelism should also be evaluated.
4. Surrogate analyte approach: stable isotope-labeled analogs are used as calibration standards, since the surrogate analyte behaves identically to the endogenous compound. As with the previous approach, parallelism needed to be evaluated.

The following sections will present studies that use various surrogate matrices to validate CSF analysis methods in solving specific research problems, including artificial CSF of different composition, saline solution, Ringer-lactate solution, various buffer solutions, and distilled water. Moreover, there are some studies, which specifically emphasizes the applicability of parallelism analysis and the addition method to assessing the acceptability of surrogate matrices for CSF analysis [137,138]. However, for practical use, the authors are free to validate both simple matrices and more complex matrices, primarily guided by ICH M10 criteria.

### 3.2. Neurotransmitters and Related Metabolic Pathways

Neurotransmitters are synthesized in neurons and released at synapses upon depolarization, where they act on specific receptors and are subsequently inactivated by reuptake or enzymatic degradation. CSF contains not only neurotransmitters themselves but also their metabolites, precursors, and related signaling molecules. Major low-molecular-weight neurotransmitters detected in CSF include amino acids (glutamic acid, γ-aminobutyric acid (GABA), glycine, and aspartic acid), monoamines (dopamine, serotonin, norepinephrine, and histamine), and acetylcholine together with its more stable metabolite, choline. In practice, metabolites such as HVA, 5-HIAA, and 3-methoxy-4-hydroxyphenylglycol (MHPG) are more frequently quantified due to their higher stability.

The metabolic pathways of these compounds are well described in the KEGG database [139], including phenylalanine, tyrosine, and tryptophan metabolism, as well as CNS-specific pathways such as serotonergic and dopaminergic synapses. Some insights into the synthesis and function of tyrosine, phenylalanine, and related monoamines in the brain, particularly in relation to diet and protein intake, are summarized in a review [140].

Most neurotransmitters and their metabolites contain amino groups (except HVA and MHPG), which generally favor LC-based methods. Nevertheless, both validated and partially validated GC–MS approaches have also been reported. Among GC–MS-based methods, an almost validated protocol for γ-hydroxybutyric acid analysis using LLE and silylation demonstrated the influence of storage conditions on in vivo formation [141]. More complex GC–MS procedures were also proposed for the determination of free and total GABA in CSF from patients with metabolic disorders [142,143].

Simplified analytical protocols are attractive for their speed and minimal sample preparation; however, they often lack sufficient sensitivity. A not fully validated (according to FDA guidelines) UPLC–QQQ method (with nonafluoropentanoic acid as an ion pairing modifier) for the analysis of 1 μL of mouse CSF almost without sample preparation allowed for the quantitative measurements of aspartic acid, serine, glycine, glutamic acid, GABA, norepinephrine, serotonin, histamine, and methylhistamine, while concentrations of epinephrine, dopamine, and acetylcholine were below the LLOQs [144]. A similar validated approach, without sufficient sample preparation, for the analysis of glutamine, glutamic acid, pyroglutamic acid, and GABA using HPLC–QQQ and heptafluorobutyric acid as an ion-pairing modifier was proposed. The protocol was applied to the analysis of rat and human CSF with concentrations of GABA (344 ± 183 vs. 89 ± 6 ng/mL) and glutamic acid (3864 ± 1540 vs. 1357 ± 50 ng/mL) higher in rats than in humans while glutamine (16,790 ± 1154 vs. 57,700 ± 3995 ng/mL) and pyroglutamic acid (685 ± 53 vs. 17,167 ± 1180 ng/mL) concentrations were lower in rats [145].

Serotonin, HVA, and 5-HIAA were analyzed using a common (protein precipitation-drying-reconstitution), fast (total run-time 6.5 min), and validated UPLC–Q–Trap protocol in CSF, serum, and urine samples of patients with and without motor impairment, resulting in the content of HVA in CSF being higher in patients with motor impairment (45 vs. 24 ng/mL, *p* = 0.036), comparable CSF levels of 5-HIAA (14.6 vs. 8.6 ng/mL, *p* = 0.198), and CSF serotonin below the LLOQ in most cases [146]. A fully validated (according to FDA and ICH 10 guidelines) protocol with protein precipitation before UPLC–QQQ analysis was proposed for the analysis of HVA, 5-HIAA, 3-orthomethyldopa, and 5-hydroxytryptophan with the subsequent quantification of the targeted analytes within the linear calibration range in CSF samples from pediatric patients suspected of neurometabolic disorders [147]. A protocol (not fully validated) with HILIC column separation almost without prior sample preparation for the UPLC–QQQ analysis of acetylcholine, histamine and its metabolites (tele-methylhistamine and tele-methylimidazolacetic acid) measured these compounds in rat CSF at the base concentration level of 0.12 ± 0.04, 0.77 ± 0.27, 0.69 ± 0.21, and 1.45 ± 0.29 ng/mL, respectively, and to demonstrate the donepezil (a reversible acetylcholinesterase inhibitor) influence on the acetylcholine concentrations [148]. Thirty neuroactive molecules and their metabolites (tyrosine and its metabolites: levodopa, 3-*O*-methyldopa, dopamine, 3-methoxytyramine, 3,4-dihydroxyphenylacetic acid, HVA, vanillylmandelic acid, and MHPG sulphate; tryptophan and its metabolites: l-kynurenine, kynurenic acid, 3-hydroxyanthranilic acid, 3-hydroxykynurenine, xanthurenic acid, anthranilic acid, quinolinic acid, cinnabaric acid, tryptamine, indoleacetic acid, 5-hydroxytryptophan, serotonin, 5-HIAA, and melatonine, together with neopterin, biopterin, dihydrobiopterin, cortisol, and histamine) were analyzed in human CSF and serum using a validated (according to EMA guidelines) and optimized protein precipitation-drying-reconstitution sample preparation and UPLC–QQQ protocol; however, vanillylmandelic acid, MHPG sulphate, cinnabaric acid, 3-hydroxyanthranilic acid, tryptamine, and serotonin were not quantified in CSF samples from patients with various neurological disorders [149].

#### 3.2.1. Derivatization Strategies

Derivatization, although an additional step that requires the purchase of specific reagents and may increase sample preparation time and variability in results, chemically modifies analytes to improve their chromatographic and mass spectrometric properties. Derivatization with benzoyl chloride adds a phenyl group to polar analytes, increasing retention on RP columns, improving sensitivity and peak resolution, and reducing ion suppression. Ten neurotransmitters and their metabolites (serotonin, HVA, 5-HIAA, noradrenaline, adrenaline, dopamine, glutamic acid, GABA, 3,4-dihydroxyphenylacetic acid (DOPAC), and histamine) were quantified after derivatization with benzoyl chloride using UPLC–QQQ within the validated concentration range in CSF samples from the rat model for human tauopathy (Alzheimer’s disease). No statistically significant changes in targeted analytes in CSF of transgenic animals with early and late stages of neurodegeneration compared to controls were observed, except for elevated adrenaline and 5-HIAA in transgenic rats [150]. This type of derivatization was extended to the targeted UPLC–QQQ analysis (a total analysis time of 33 min) of 70 neuroactive molecules, including acetylcholine, adenosine, polyamines, indoleamines, amino acids, catecholamines, amines, energy compounds, antioxidants, and their metabolites; however, it was not properly validated (only LOD, carryover, CV, and *R^2^* for calibration curves were described). Half of all compounds were detected in a pooled CSF from healthy donors in concentrations exceeding LODs [151]. A sensitive approach with propyl chloroformate derivatization and UPLC–QQQ analysis was validated according to EMA guidelines for the successful quantification of 16 monoamine neurotransmitters and their metabolites (dopamine, D–PAC, epinephrine, 5-HIAA, 5-hydroxytryptophan, HVA, MHPG, 3-methoxytyramine, vanillylmandelic acid, vanillactic acid, serotonin, metanephrine, normetanephrine, norepinephrine, 3-*O*-methyl-dopa, and levodopa) in 200 CSF samples of pediatric patients, revealing age-dependent reference intervals [152]. Solid-phase extraction at the sample preparation step was proposed for the UPLC–QQQ validated analysis of dopamine, epinephrine, norepinephrine, metanephrine, normetanephrine, and 3-methoxytyramine in human CSF and plasma. Despite all compounds being measured in real patient samples from patients with Alzheimer’s disease and controls, the large sample volume of 500 μL required for labor-intensive solid-phase extraction makes the proposed method impractical for routine use [153].

#### 3.2.2. Preanalytical and Physiological Factors

Since 5-HIAA, a serotonin metabolite, and HVA, a dopamine metabolite, are frequently detected in CSF, two articles by the same research group that noted changes in their concentrations depending on the time of CSF collection after lumbar puncture are of interest. In the first study, CSF was collected from healthy donors 3 h after spinal canal cannulation, over 6 h at 10 min intervals, and again 6 weeks later at 30 min intervals. The results demonstrated that these metabolites closely covaried over time (correlation coefficient = 0.9) and showed a relatively constant HVA/5-HIAA ratio of 2.2 ± 0.7. Despite metabolite determination being performed using non-validated HPLC with electrochemical detection, two different methods and HPLC equipment were used in two groups, yielding similar results [154]. In the second study, HVA and 5-HIAA concentrations immediately after lumbar puncture were only half of the average levels detected several hours later. At the same time, the HVA/5-HIAA ratio of approximately 2.5 remained stable throughout the lumbar puncture [155]. Interestingly, a study devoted to the assessment of HVA, 5-HIAA, HVA/5-HIAA, MHPG, and HVA/MHPG in the CSF samples from a large groups of suicide attempters (*n* = 120) and controls (*n* = 47), obtained by both GC–MS and HPLC non-validated methods, demonstrated very similar HVA/5-HIAA ratio of 2.1 ± 0.6 in controls and different absolute HVA and 5-HIAA concentrations [156], compared to [154], and lower HVA/5-HIAA ratio of 1.6 ± 0.5 in suicide attempters (*p* = 0.0001) [156]. These results are very valuable, demonstrating that absolute concentrations of individual metabolites may be clinically uninformative or subject to external influences (such as performing a lumbar puncture). Particularly, a large review article concluded that absolute low 5-HIAA concentrations are not characteristic of suicidal behavior, citing conflicting results from various studies and methodological shortcomings [157].

#### 3.2.3. Tryptophan Metabolism

Given the extensive number of validated methods targeting tryptophan metabolism, these approaches are considered separately. A validated UPLC–QQQ protocol for the quantification of 13 metabolites from tryptophan–kynurenine and nitric oxide pathways in CSF, which was mentioned in Section 2.8, included two separate sample preparations with (for tryptophan, kynurenine, kynurenic acid, xanthurenic acid, 3-hydroxyanthranilic acid, and quinolinic acid) and without protein precipitation (for neopterin, 3-hydroxykynurenine, anthranilic acid, picolinic acid, arginine, citrulline, and methylhistamine); however, not all analytes were subsequently quantitatively measured in all CSF samples of patients with acute encephalitis and controls, such as 3-hydroxykynurenine, xanthurenic acid, anthranilic acid, 3-hydroxyanthranilic acid, picolinic acid, and methylhistamine. Nevertheless, elevated kynurenine/tryptophan ratio, quinolinic acid/kynurenic ratio, and anthranilic acid/3-hydroxyanthranilic acid ratio (all *p* < 0.01) were observed during acute neuroinflammation [115]. This protocol, targeted for nine tryptophan–kynurenine metabolites, was applied for the analysis of CSF samples from patients with epileptic spasms compared with other epilepsy syndromes, other non-inflammatory neurological diseases, and inflammatory neurological controls. It resulted in the lower kynurenic acid and kynurenic acid/kynurenine ratio levels in children with epileptic spasms compared with other inflammatory neurological conditions (both *p* < 0.0001), together with kynurenic acid, kynurenine, and quinolinic acid being higher in the neuroinflammation group compared with the epileptic spasms (all *p* < 0.0001) [116]. Further, metabolites from the tryptophan–kynurenine and nitric oxide pathways were quantified in a large cohort of pediatric patients (almost 350 children), including inflammatory and epilepsy groups, compared with neurogenetic and structural disorders, neurodevelopmental disorders, psychiatric and functional neurological disorders, and headache. Higher CSF neopterin, kynurenine, quinolinic acid, and kynurenine/tryptophan ratio were observed in the inflammation group compared to all control groups (all *p* < 0.0003); kynurenic acid/kynurenine ratio was lower in the epilepsy group compared to all control groups (all *p* ≤ 0.0003) [117]. Tryptophan and its 8 kynurenine-type metabolites, including kynurenic acid, kynurenine, xanthurenic acid, anthranilic acid, quinolinic acid, 3-hydroxykynurenine, picolinic acid, 3-hydroxyanthranilic acid, were analyzed in CSF without any sample preparation before HPLC–QQQ using an optimized and validated protocol according to FDA guidelines, with all analytes, except for xanthurenic acid, being quantified in real samples [158]. Tryptophan and its 16 metabolites (kynurenic acid, kynurenine, 3-hydroxykynurenine, indole-3-acetic acid, 5-HIAA, tryptamine, serotonin, melatonin, *N*^1^-acetyl-*N*^2^-formyl-5-methoxykynuramine, anthranilic acid, 3-hydroxyanthranilic acid, *N*-methylserotonin, 5-hydroxytryptophan, *N*-methyltryptamine, 5-methoxytryptamine, and *N*-acetylserotonin) in human serum and CSF were analyzed by an optimized UPLC–QQQ protocol with common sample preparation (protein precipitation-drying-reconstitution). Despite the authors stating that they follow the FDA and EMA guidelines, matrix effects and accuracy for several analytes were inappropriate; nevertheless, the authors analyzed CSF samples from healthy donors and did not measure 9 from 16 metabolites, including serotonin, *N*-methylserotonin, *N*-acetylserotonin, 3-hydroxyanthranilic acid, tryptamine, *N*-methyltryptamine, 5-methoxytryptamine, melatonin, and *N*^1^-acetyl-*N*^2^-formyl-5-methoxykynuramine [159]. Thus, the developed protocol, particularly the selected calibration ranges, is unsatisfactory, casting doubt on the results of the subsequent clinical study [160].

Several GC–MS-based protocols included a validated method using solid-phase extraction and large volume of CSF (3 mL) for the analysis of 5-hydroxyindole-3-ethanol, 5-HIAA, 5-hydroxytryptophan, and serotonin in children with acute lymphoblastic leukemia [161] and almost validated method for the analysis of quinolinic, picolinic, and nicotinic acids [162] with its subsequent clinical application on patients with multiple sclerosis [163], patients with HIV-associated neurocognitive disorders [164], and two studies on malaria [165,166], demonstrating that higher quinolinic and picolinic acids in malaria group compared to controls are associated with a fatal outcomes.

Summarizing the section on validated methods for determining neurotransmitters and their metabolites, a wide range of analytical approaches has been described, with the majority based on UPLC–QQQ platforms. These protocols range from simple procedures to more elaborate workflows involving derivatization or solid-phase extraction, enabling the simultaneous quantification of numerous analytes within clinically relevant concentration ranges. In comparison, GC–MS-based methods are generally less suitable for high-throughput multi-analyte analysis due to longer run times and more demanding sample preparation. Particular attention should be paid to the quality of method validation, including the selection of validation criteria and their compliance with the relevant guidelines. Importantly, multiple studies show that metabolite ratios may provide more robust and clinically informative biomarkers than absolute concentrations, which are influenced by preanalytical and physiological factors. Overall, while the analytical toolbox for neurotransmitter analysis in CSF is well established, further standardization and rigorous validation remain essential for reliable clinical translation.

### 3.3. Amino Acids, Their Derivatives, and Other Organic Acids

Although several amino acids and organic acids are discussed in the previous section in the context of neurotransmission, this section focuses on broader analytical strategies targeting amino acids, their derivatives, and structurally diverse organic acids, including both endogenous and microbiota-derived metabolites.

#### 3.3.1. Amino Acid Profiling and General Metabolic Features

A GC–TOF method with atmospheric pressure chemical ionization enabled the targeted quantification of multiple amino acids and related metabolites (valine, alanine, methionine, sarcosine, leucine, phenylalanine, tyrosine, proline, isoleucine, aspartic acid, benzoic acid, glycine, serine, threonine, glutamic acid, Phenyl-Gly, hippuric acid, caffeine, theophylline, lysine, 4-methyldopamine, dopamine, uric acid, 5-HIAA, and nortriptyline); however, despite the detection of over 300 features, only a limited number of compounds were confidently identified [167]. A validated GC–MS protocol based on microwave-assisted derivatization was successfully applied to the determination of a broad panel of amino acids (glycine, sarcosine, alanine, proline, valine, leucine, phenylalanine, lysine, isoleucine, serine, threonine, methionine, aspartic acid, cysteine, glutamic acid, and asparagine) in CSF samples from patients with acquired immunodeficiency syndrome [168].

Dynamic aspects of amino acid metabolism have also been explored. In a longitudinal study using an unvalidated GC–MS method, circadian variations were observed, with increased nocturnal levels of leucine and isoleucine, and significant correlations between CSF and plasma concentrations of multiple amino acids. These findings highlight the importance of physiological variability in interpreting analytical results [169].

#### 3.3.2. Nitrogen Metabolism and Related Biomarkers

Targeted LC–MS/MS approaches have been applied to nitrogen metabolism, particularly the arginine–nitric oxide pathway. A HILIC–HPLC–QQQ method enabled the quantification of arginine and its methylated derivatives, asymmetric and symmetric dimethylarginine, in CSF samples from patients with subarachnoid hemorrhage. The observed increase in arginine and asymmetric dimethylarginine concentrations (19.4 ± 5.5 vs. 50.2 ± 28.2 µM and 0.068 ± 0.021 vs. 0.196 ± 0.138 µM, respectively, *p* < 0.001), together with a decrease in their ratio (335 vs. 275, *p* = 0.023), was associated with impaired nitric oxide production and disease progression [170]. A validated UPLC–QQQ method based on in-tube solid-phase microextraction allowed for the determination of homocysteine and homocysteic acid. Elevated homocysteine levels were reported in patients with Alzheimer’s disease (mean 31.98 ng/mL) and mild cognitive impairment (36.38 ng/mL) compared to controls (24.11 ng/mL), supporting its role as a potential biomarker of neurodegeneration; however, homocysteine acid was not detected in any of the samples [171].

#### 3.3.3. Aromatic and Microbiota-Derived Organic Acids

Several studies have focused on aromatic acids derived from the metabolism of phenylalanine, tyrosine, and tryptophan, many of which are partially or entirely of microbial origin. Almost validated (according to FDA guidelines) conditions based on traditional LLE and semi-automatic microextraction by packed sorbent were proposed for the GC-MS analysis of silyl derivatives of phenylalanine (benzoic, 3-phenylpropionic, and 3-phenyllactic acids) and tyrosine (4-hydroxybenzoic, 4-hydroxyphenylacetic, HVA, 4-hydroxyphenylpropionic, and 4-hydroxyphenyllactic acids) metabolites. Both sample preparation conditions provided equivalent results, while the microextraction approach required less CSF than LLE (40 vs. 200 µL, respectively). 4-Hydroxyphenyllactic, benzoic, 3-phenyllactic, HVA, 4-hydroxybenzoic, and 4-hydroxyphenylacetic acids were quantified in more than half of CSF samples from neurosurgical patients with suspected secondary meningitis [172]. A more sensitive protocol on a wider range of aromatic acids, mostly of microbial origin, including tryptophan (5-HIAA, indole-3-carboxylic, indole-3-acetic, indole-3-propionic, and indole-3-lactic acids), phenylalanine (3-phenylpropionic and 3-phenyllactic acids), and tyrosine (4-hydroxybenzoic, 4-hydroxyphenylpropionic, 4-hydroxyphenylacetic, and 4-hydroxyphenyllactic acids) metabolites, was developed using a common protein precipitation-drying-reconstitution sample preparation of CSF and validated (according to FDA and ICH guidelines) UPLC–QQQ analysis. All analytes, except for 3-phenylpropionic and 4-hydroxyphenylpropionic acids, were detected in CSF samples of post-neurosurgical patients with 3-phenyllactic, 4-hydroxyphenyllactic, indole-3-lactic, and indole-3-carboxylic acids higher in patients with signs of secondary bacterial meningitis compared to those without signs of secondary bacterial meningitis (*p* ≤ 0.027) [173]. Subsequently, this protocol, together with a validated serum analysis protocol, was applied in two clinical studies. The first study compared profiles of aromatic acids in simultaneously collected CSF and serum samples from patients with secondary meningitis and those without CNS infection and found that, in patients with CNS infection, 4-hydroxyphenyllactic acid in CSF was higher than in serum, suggesting a local (CNS) or microbial origin [174]. In the second study with larger patient cohorts, 4-hydroxyphenyllactic, 3-phenyllactic, and indole-3-lactic acids in CSF were statistically higher in patients with secondary meningitis (*p* < 0.001), and several univariate and multivariate prognostic models were constructed with high meningitis-predictive characteristics (AUC–ROC > 0.91) [175]. Another quantitative but not validated UPLC–QQQ study on over 50 host and microbial phenolic metabolites, including 4-hydroxybenzoic acid, m-tyramine, 4-, and 3-hydroxybenzoic acids, 3-phenylpropionic acid, 2-, 3-, and 4-hydroxyphenylacetic acids, dopamine, 4-hydroxycinnamic acid, L-phenylalanine, 2-, 3-, and 4-hydroxyphenylpropionic acids, norepinephrine, 4-hydroxyphenylpyruvic acid, 3,4-dihydroxyhydrocinnamic acid, 4-hydroxyphenyllactic acid, HVA, 2,4- and 2,3-dihydroxyhydrocinnamic acids, 4-hydroxy-3-methoxymandelic acid, 4- and 3-hydroxyhippuric acids in CSF, serum, and urine was proposed and used additional analytical methods, such as NMR and HPLC–UV, for the structural identification and subsequent quantitative determination [176]. In addition to aromatic metabolites, other bacterial-derived compounds such as muramic acid, a component of peptidoglycan, have also been detected in CSF samples from patients with pneumococcal meningitis using GC–MS/MS, further supporting the presence of microbial signatures in CNS infections [177].

Indole-containing tryptophan metabolites, which are alternative to kynurenine metabolites and are microbial in origin, may play a role in the gut microbiota-brain axis concept [178]. These metabolites are indoleacetic, indolepropionic, indoleacrylic, indolelactic, and indolepyruvic acids; indolealdehyde and indole acetaldehyde; tryptamine; indole; and skatole. They are normally detectable in the CSF of both humans and rodents, and a review article summarizes their concentrations in CSF, blood, brain, saliva, and feces [179].

#### 3.3.4. Individual Biomarkers

Several studies have examined individual metabolites of clinical relevance using both GC–MS and LC–MS/MS. A fully validated HPLC–MS/MS protocol for free and total sialic (N-acetyl neuraminic) acids (its conjugated form was calculated as the difference between total and free forms) using protein precipitation was developed and applied for the analysis of more than 200 CSF samples to reveal reference values of the target analytes (mean ± standard deviation, μM): free 11.8 ± 4.2; total 28.4 ± 9.2; and conjugated 16.6 ± 6.0 sialic acids [180]. Other GC–MS-detected compounds include creatine and its precursor, guanidinoacetate, in inherited metabolic disorders, where characteristic alterations depending on the enzymatic deficiency were revealed [181]; and N-acetylaspartic acid, a neuro-specific marker, which was found to differ among clinical forms of multiple sclerosis [182]. Additional studies have explored nervonic acid as a potential biomarker of psychiatric disorders (GC–TOF analysis) [183] and 5-methyltetrahydrofolate as a key component of one-carbon metabolism (HPLC–QQQ analysis) [184]. However, many of these approaches rely on partially validated methods or are limited to single-analyte determination, which may restrict their broader applicability.

To summarize Section 3.3, a wide range of analytical strategies has been developed, encompassing both single and multi-analyte targeted approaches based on GC–MS and LC–MS/MS platforms. While GC–MS methods remain valuable for the analysis of specific compound classes, LC–MS/MS approaches—particularly UPLC–QQQ—are increasingly preferred due to their higher throughput, simplified workflows, and broader analytical scope. Particular attention should be paid to the rigor of method validation, as incomplete or inconsistent validation remains a common limitation that may compromise data reliability. Importantly, multiple studies highlight the clinical relevance of metabolic alterations, including changes in amino acid metabolism, nitrogen-related pathways, and microbiota-derived aromatic acids, which demonstrate significant diagnostic and prognostic potential.

### 3.4. Energy and Lipid Metabolism and Cofactors

In order not to repeat the information that was described in the review on GC–MS analysis of CSF [22], studies devoted to the unvalidated analysis of glucose metabolites [185,186]; prostanoids [187,188]; F_2_-isoprostanes and other metabolites of arachidonic acid [189,190,191,192,193,194] together with F_4_-neuroprotanes, which are metabolites of docosahexaenoic acid [195,196], will not be described in this section. Only one LC–MS-based study on arachidonic acid-related endocannabinoids will be discussed. Sixteen endocannabinoids and their analogues (α-linolenoyl, palmitoyl, palmito-leoyl, pentadecanoyl, dihomo-γ-linolenoyl, eicosa-pentaenoyl, docosatetraenoyl, docosahexaenoyl, linoleoyl, stearoyl, and *N*-oleoyl ethanolamides, anandamide, 1- and 2-arachidonoyl glycerols, 1- and 2-linoleoyl glycerols, 1- and 2-oleoyl glycerols) were analyzed using sensitive and validated micro-LC–Q–Trap conditions; subsequently, reference concentrations of two of these naturally occurred endocannabinoids (anandamide 1.0–7.1 pM and 2-arachidonoyl glycerol 87.9–658.5 pM) were measured in almost one hundred of healthy donors [197].

#### 3.4.1. Cholesterol Metabolites

The human brain contains 2% of cholesterol that is synthesized de novo in the CNS; its metabolites include oxysterols, bile acids, and steroids. Neurosteroids are steroids that are synthesized or biologically active in the CNS; similarly, cholesterol’s main metabolite, 24S-hydroxycholesterol, is a neurosterol [198,199]. This section is devoted to the analysis of oxysterols and bile acids, while the next section, Section 3.4.2, describes neurosteroids.

In a wide-range cholesterol metabolite analysis, which included cholesterol itself, 17 oxysterols, and 17 free and conjugated bile acids in CSF and plasma, a fast sample preparation including protein precipitation and on-line solid-phase extraction before UPLC–Q–Trap resulted in quantification of one free oxysterol, five free and five conjugated bile acids in CSF with no significant differences between patients with and without blood–brain barrier disturbance. Despite the protocol being validated in accordance with EMA guidelines, the accuracy and precision of some analytes exceeded acceptable limits [200]. A two-dimensional HPLC–Q–Trap analysis of a single 24S-hydroxycholesterol was proposed after LLE and derivatization, with the addition of 2-hydroxypropyl-β-cyclodextrin to the initial CSF samples to eliminate nonspecific binding. However, no absolute concentrations were described during real CSF sample analysis [201]. Two unvalidated quantitative studies include the analysis of twelve oxysterols and cholestenoic acids, which were measured in the concentration range of 5 pg/mL–2.6 ng/mL using unvalidated enzyme assisted derivatization and LC–MS/MS analysis in mouse CSF [202], and two dihydroxyoxocholestenoic acids (7a,24- and 7a,25-dihydroxy-3-oxocholest-4-en-26-oic acids) using HPLC–Orbitrap analysis after derivatization [203].

#### 3.4.2. Neuroactive Steroids

Several GC–MS protocols were almost validated for the analysis of a wide range of neuroactive steroids. Androsterone, testosterone, allopregnanolone, and pregnenolone, but not isopregnanolone and dihydrotestosterone, were quantified in human CSF (1–2 mL) in higher concentrations than in monkey CSF after solid-phase extraction and derivatization [192,204]. A wider range of analytes, including dehydroepiandrosterone, progesterone, androstenedione, epiandrosterone, 7α-hydroxy-dehydroepiandrosterone, 7β-hydroxy-dehydroepiandrosterone, 5-androstene-3β,7α,17β-triol, 5-androstene-3β,7β,17β-triol, 16α-hydroxy-pregnenolone, 16α-hydroxy-dehydroepiandrosterone, and 16α-hydroxy-progesterone, was analyzed in 1 mL of CSF after LLE and a common two-step derivatization. All neurosteroids were quantified in real samples, with significant correlations observed between the 7α/β-hydroxymetabolites of dehydroepiandrosterone and androstenediol in serum and CSF [205]. More advanced GC–MS/MS quantification of dehydroepiandrosterone, testosterone, dihydrotestosterone, androstenedione, estrone, estradiol, and progesterone was applied for analysis of corresponding CSF and serum samples from almost 50 healthy volunteers. These hormones were quantified in all samples, except progesterone, which was not measured in CSF; moreover, the authors proposed several interesting hypotheses regarding the origin of these sex steroids in CSF [206]. Dehydroepiandrosterone and its three 7-oxo- and 7-hydroxymetabolites, 16a-hydroxydehydroepiandrosterone, cortisol, and cortisone, were analyzed in CSF (3 mL) and plasma samples using UPLC–QQQ after extraction and derivatization, with all analytes except 7-oxo-dehydroepiandrosterone successfully analyzed in real samples [207]. To determine four estrogens, including estrone, estriol, 17a- and 17b-estradiol, conditions including two-dimensional LC–MS/MS after LLE and derivatization were proposed; however, only 17b-estradiol was quantified in two out of three CSF samples from patients after ischemic stroke [208].

Some unvalidated studies describe an approach based on HPLC purification before GC–MS analysis, particularly for the analysis of a wide range of neurotransmitters and neurosteroids in patients with post-traumatic stress disorder [209]; dehydroepiandrosterone and pregnenolone in patients with Alzheimer’s disease [210]; and allopregnanolone, dehydroepiandrosterone, and pregnenolone in patients with relapsing-remitting multiple sclerosis [211].

Overall, both GC–MS- and LC–MS/MS-based approaches have been applied to the analysis of lipid-related metabolites in CSF, with comparable numbers of methods developed for each platform. In contrast to other metabolite classes, neither approach demonstrates a clear analytical advantage, as both require extensive, labor-intensive sample preparation procedures, often including extraction and derivatization steps, and require relatively large CSF volumes. Despite these efforts, quantitative determination of all targeted analytes is often not achieved, even in studies declared to be validated. This limitation, combined with the complexity of lipid metabolism and low endogenous concentrations, highlights the need for further methodological improvements and stricter validation to ensure reliable and comprehensive profiling of lipid-related metabolites in CSF.

#### 3.4.3. Vitamin B6

Vitamin B6 itself is not a metabolite; however, it is an important cofactor for the metabolic reactions of amino acids, neurotransmitters, and other biomolecules in the CNS, and its analysis is also mentioned in this review. Furthermore, it exists in various forms (vitamers), such as pyridoxal, pyridoxine, pyridoxamine, pyridoxic acid, and their phosphate ester forms; thus, their simultaneous analysis is an interesting issue addressed in two LC–MS-based studies. All seven forms were analyzed in the first fully validated study after simple protein precipitation and UPLC–QQQ analysis with pyridoxal (14.8–42.5 nM), pyridoxamine (0.1–0.5 nM), pyridoxic acid (0.09–4.1 nM), and pyridoxal 5′-phosphate (8.8–42.0 nM) being quantified in real CSF samples [212]. The second validated (according to ICH M10) study with similar conditions is devoted to the analysis of pyridoxal 5′-phosphate, pyridoxal, pyridoxine, pyridoxamine, and pyridoxic acid, with only pyridoxal 5′-phosphate and pyridoxal being quantitatively measured [213]. This study may be of interest, as the analytical conditions and results were compared with several previously described studies, including [212]; thus, they will be discussed here.

### 3.5. Conclusions to Targeted CSF Metabolomics

The number of validated chromatographic–mass spectrometric methods for the determination of metabolites in CSF appears to be substantially lower than the number of studies devoted to untargeted metabolomic profiling (36 vs. >60), a brief summary description of which is provided in Table 3 with more details in Supplementary Table S1. This imbalance reflects both the analytical complexity of CSF as a matrix and the significant methodological efforts and human labor costs required to achieve full validation in accordance with regulatory guidelines.

Among the validated approaches, LC-based techniques, predominantly UPLC–QQQ, clearly prevail. At the same time, methods involving minimal sample preparation are relatively rare. This is primarily due to extremely low concentrations of many analytes in CSF, which require additional sample preparation steps, such as preconcentration, extraction, or derivatization, to achieve sufficient sensitivity and selectivity. As a result, most validated protocols rely on multi-step workflows that increase analysis time and potential variability, but remain essential for reliable quantification.

A critical issue identified across the reviewed studies is the highly variable quality of method validation. In many cases, references to established validation guidelines (e.g., FDA, EMA, ICH M10) are absent altogether. Even when such guidelines are cited, this does not necessarily imply full compliance. Common shortcomings include inconsistencies between the lower limit of quantification and the lowest calibration point, leading to the reporting of two different values that contradict regulatory requirements. In addition, matrix effects-typically assessed via the matrix factor-are either insufficiently evaluated or exceed acceptable limits in some studies.

Another important limitation concerns the selection of calibration ranges. In several cases, validated linear ranges do not cover clinically relevant concentration intervals. While this may be justified in exploratory or pilot studies for poorly characterized analytes, such situations are relatively rare. In most cases, this mismatch significantly reduces the clinical applicability of the developed methods, even when formal validation criteria are met.

Finally, the level of clinical demonstration of the proposed methods remains generally low. Only a limited number of studies apply validated methods to establish reference ranges or to address clearly defined clinical purposes. Such studies are particularly valuable, as they bridge the gap between analytical development and clinical application. However, the majority of publications rely on relatively small sample sets and focus primarily on methodological feasibility rather than clinical relevance.

Overall, despite considerable progress in developing validated chromatographic–mass spectrometric methods for CSF analysis, further efforts are required to improve validation rigor, ensure the clinical relevance of analytical ranges, reduce sample preparation complexity where possible, and expand well-designed clinical applications. These steps are essential for reliably translating analytical methodologies into routine clinical and research practice.

## 4. Conclusions and Future Perspectives

Chromatography–MS-based analysis of CSF has become a cornerstone of modern neurometabolomics, enabling comprehensive characterization of metabolic alterations associated with CNS disorders. As summarized in this review, both untargeted and targeted approaches provide complementary insights: untargeted metabolomics facilitates the discovery of novel metabolic signatures, whereas targeted methods enable their quantitative validation and potential clinical translation.

Despite substantial analytical advances, several critical challenges remain. One of the most significant limitations is the lack of standardization across the entire metabolomics workflow, including sample collection, storage, preparation, data acquisition, and processing. Variability in these steps continues to hinder reproducibility and cross-study comparability. In addition, the inherent complexity of CSF as a biological matrix, combined with limited sample availability, complicates both method development and validation. The predominance of semi-quantitative data in untargeted studies and the limited number of fully validated targeted assays further contribute to the gap between biomarker discovery and clinical implementation.

Another important limitation is the biological and analytical variability observed in CSF metabolomics studies. Many reported biomarkers exhibit high sensitivity but insufficient specificity, often reflecting general pathophysiological processes rather than disease-specific mechanisms. This underscores the need to move beyond single-metabolite biomarkers toward multidimensional metabolic signatures and pathway-based interpretations.

Future progress in the field will depend on several key directions. First, the development and widespread adoption of standardized protocols and reporting guidelines are essential to improve reproducibility and facilitate data integration across studies. Second, advances in analytical technologies, including higher-resolution and higher-sensitivity MS, improved chromatographic separation, and miniaturized workflows for low-volume samples, will further enhance metabolome coverage and sensitivity. Third, integrating metabolomics with other omics approaches, such as proteomics, transcriptomics, and lipidomics, is expected to provide a more comprehensive understanding of CNS pathophysiology and to improve biomarker robustness.

In addition, large-scale, well-designed clinical studies with adequately powered cohorts and appropriate control groups are critically needed to validate candidate biomarkers and assess their clinical utility. Such studies must take into account numerous factors, including environmental influences, diet, sex, age, medications, and comorbidities, in comparison with controls. These factors contribute to the complexity and heterogeneity of metabolomics studies and biomarker validation. The increasing use of advanced statistical modeling, machine learning, and metabolome-wide association studies also holds promise for identifying clinically relevant metabolic patterns and improving diagnostic accuracy.

The translation of CSF metabolomics into clinical practice will require not only analytical validation but also demonstration of clinical relevance, cost-effectiveness, and feasibility within routine diagnostic workflows. Close collaboration between analytical chemists, clinicians, and bioinformaticians will be essential to achieve these goals.

In conclusion, CSF neurometabolomics represents a rapidly evolving and highly promising field. While significant challenges remain, continued methodological improvements and interdisciplinary efforts are likely to transform metabolomic findings into clinically actionable tools for the diagnosis, prognosis, and monitoring of neurological diseases.

## Figures and Tables

**Table 1:** The brief description of the studies devoted to the untargeted CSF metabolomics.

Ref.	Analytical Methods	CSF Volume; Sample Preparation	Real Samples	Detected and Dysregulated Compounds (Compared to Controls)
Aging				
[60]	HPLC–Q–Orbitrap (Thermo Accucore aQ RP C18 column)	100 µL CSF; extraction (410 µL MeOH), drying, and resuspension (100 µL 5% MeOH-95% H_2_O and 0.1% formic acid)	CSF samples from healthy volunteers (*n* = 23)	Total 70. Increased with age: isoleucine, acetylcarnitine, pipecolate, methionine, glutarylcarnitine, 5-hydroxytryptophan, ketoleucine, and hippurate. Decreased: methylthioadenosine and 3-methyladenine.
[61]	HPLC–Q–TOF (Waters XBridge BEH Amide column), ESI(+) and ESI(−) HILIC–UPLC–QQQ	HILIC–UPLC–QQQ: 2 μL CSF for ESI(+) and 10 μL CSF for ESI (−). HPLC–Q–TOF: 200 μL CSF, protein precipitation (1 mL MeOH), drying, and resuspension (40% H_2_O:60% ACN)	CSF samples from healthy volunteers (*n* = 85), patients with Alzheimer’s disease (*n* = 57), and patients with Parkinson’s disease (*n* = 56)	HILIC–UPLC–QQQ: targeted metabolomics analysis of 203 metabolites. HPLC–Q–TOF: total 6735 features. Globally optimized targeted MS: total 854 features. Lipidomics: total 1070 features. Targeted metabolomics. Increased with age: xanthine, kynurenine, carnitine, 5HIAA, and cystine. Decreased: 4-aminobutyric acid, serine, and uridine. Lipidomics. Increased: sphingomyelin (18:1) [SM(18:1)], SM(16:0), triacylglycerol 52:2-FA 18:1, dihydroceramide (24:1), free fatty acids (24:0), and SM(14:0). Decreased: hexosylceramide (24:1) and phosphatidylethanolamine (O18:0/22:4)
[62]	ESI(+) and ESI(−) UPLC–Q–TOF (ACQUITY UPLC BEH Amide column)	50 μL CSF; protein precipitation (200 μL MeOH), drying, and resuspension (200 μL 50% ACN)	CSF samples from healthy volunteers (*n* = 92): young (*n* = 34, aged 20–39), middle-aged (*n* = 31, aged 40–59), and elderly (*n* = 27, aged 60 and above)	Total 162. Positive associations with age: pantothenic and aspartic acids, cysteine, 5HIAA, and glutamate. Negative correlations: asparagine and glycerophosphocholine. Increased taurine and 5HIAA in females.
Autism Spectrum Disorders and Depression Disorders				
[69]	UPLC–Q–Orbitrap (Acquity UPLC HSS T3 Column)	100 μL CSF; protein precipitation (MeOH), drying, and resuspension	CSF samples from patients with autistic regression (*n* = 22), controls with neurodevelopmental disorders (but not autistic regression) (*n* = 16) and controls with other neurological diseases (headache, encephalitis, epilepsy) (*n* = 34)	In the untargeted case–control 1 study, metabolic differences were identified in 127 CSF metabolite levels (113 upregulated and 14 downregulated) between autistic regression and controls. In the untargeted case–control 2 study, differences were identified in 114 CSF metabolites (78 upregulated and 26 downregulated) between autistic regression and controls. Increased: sphingosine-1-phosphate. Decreased: β-hydroxybutyrate
[43]	GC–MS (HP–5MS column)	15 μL CSF; protein precipitation (90 μL MeOH), drying, two-step derivatization (30 μL methoxamine hydrochloride and 30 μL *N*,*O*-bis(trimethylsilyl)trifluoroacetamide (BSTFA))	CSF samples from female macaques (*n* = 10) and age- and gender-matched healthy controls (*n* = 12)	Total 663. Increased: propanoic acid, acetic acid, hydroxylamine, propanedioic acid, butanoic acid, proline, methanamine, glycine, isothiourea, nonanoic acid, carbamic acid, threonine, beta-alanine, threitol, erythronic acid, l-aspartic acid, xylitol, ribitol, 2-keto-d-gluconic acid, 1,4-butanediamine, d-fructose, myoinositol, glucaric acid, hexadecanoic acid, scyllitol, gulose, heptadecanoic acid, linolelaidic acid, trans-9-octadecenoic acid, oleic acid, octadecanoic acid, *N*-acetyl-d-glucosamine, d-glycero-d-galacto-heptitol, galactitol, 5-phenylvaleric acid. Decreased benzeneacetic acid and 1*H*-indole-2-carboxylic acid.
[71]	GC–MS	No data	CSF samples from parturients undergoing caesarean delivery (*n* = 75)	Total 51. Increased: 33 metabolites. Decreased: 18 metabolites. Capric, dodecanoic, arachidic, and behenic acids were negatively correlated, and l-tryptophan positively correlated with postpartum depression symptoms
[72]	LC–MS	No data	CSF samples from transcranial magnetic stimulation at baseline and at the end of the 6-week treatment (*n* = 5)	Total 72. Increased: niacinamide, kynurenine, and creatinine. Decreased: cystine
Multiple Sclerosis				
[76]	UPLC–Q–TOF (targeted and nontargeted metabolomics and lipidomics), GC-MS	Nontargeted UPLC–Q–TOF: 10 μL CSF; protein precipitation (30 μL MeOH), drying, and resuspension (H_2_O). GC–MS (for lipids): 25 μL CSF; extraction with chloroform:MeOH (2:1, *v*/*v*)	CSF samples from multiple sclerosis patients (*n* = 11) and from individuals with non-inflammatory CNS disorders (*n* = 12)	Increased: 8-iso-prostaglandin F_2_α, iodotyrosine, p-hydroxyphenylacetic acid, *N*^1^-methyl-2-pyridone-5-carboxamide, 3,4-dihydroxyphenylacetaldehyde, niacinamide, anserine, *N*^1^-methyl-4-pyridone-3-carboxamide, and dihydrouracil. Decreased: metanephrine, tyrosine, hippuric acid, 2-hexaprenyl-6-methoxyphenol, retinaldehyde, *N*-(2-hydroxyethyl)-docosanamide, and β-hydroxyisobutyrate
[77]	MALDI–TOF, HPLC–QQQ	HPLC–QQQ: 9.6 μL CSF; extraction solution from the NeoBase Non-derivatized MS/MS Kit	CSF samples from patients with relapsing-remitting multiple sclerosis (*n* = 13) and patients with other neurological disorders (*n* = 12)	MALDI–TOF nontargeted lipidomics and targeted HPLC–QQQ analysis of free carnitine, amino acids, and acylcarnitines. Increased: glutamate.
[78]	ESI(+) and ESI(−) HPLC–Q–Orbitrap (Thermo Accucore aQ RP C18 column)	100 μL CSF; protein precipitation (MeOH), drying, and resuspension (100 μL 5% MeOH, 0.1% formic acid, and 94.9% H_2_O)	CSF samples from a secondary progressive multiple sclerosis group (*n* = 30), a relapsing-remitting multiple sclerosis group (*n* = 16), and a control group (*n* = 10)	Total 117. 21 metabolites were statistically different between the two types of multiple sclerosis. Altered levels of tryptophan metabolites (kynurenate, 5-hydroxytryptophan, 5HIAA, and *N*-acetylserotonin) were observed in patients with secondary progressive multiple sclerosis compared with relapsing-remitting multiple sclerosis and controls. The secondary progressive multiple sclerosis group had altered kynurenine in comparison with the relapsing-remitting multiple sclerosis group and altered indole-3-acetate in comparison with controls. The secondary progressive multiple sclerosis group differed in pyrimidine metabolite levels (uridine and deoxyuridine) compared with the relapsing-remitting multiple sclerosis group and controls, and in thymine and glutamine compared with the relapsing-remitting multiple sclerosis group
[79]	ESI(+) and ESI(−) HPLC–QQQ (column provided with the kit)	30 μL CSF; extraction, drying, derivatization (50 µL 5% solution of phenylisothiocyanate (in H_2_O:MeOH:pyridine, 1:1:1), drying, extraction (300 µL 5 mM ammonium acetate in MeOH)	CSF samples from patients with secondary progressive multiple sclerosis (*n* = 12) and healthy individuals (*n* = 12)	Commercial kit for 408 metabolites: 196 glycerophospholipids, 60 glycerides, 55 acylcarnitines, 40 sphingolipids, 21 amino acids, 21 biogenic amines, 14 cholesteryl esters and 1 monosaccharide, including the sum of hexoses. Total 35. Increased: glycine, asymmetric dimethylarginine, glycerophospholipid PC–O (34:0), and total hexoses
[80]	NMR, GC–MS (TG–5MS column), LC–QQQ, FIA–QQQ	LC–QQQ, FIA–QQQ: 30 µL CSF, BiocratesAbsoluteIDQ p180 Kit. GC–MS: 200 µL CSF; lyophilization; drying, two-step derivatization (50 μL methoxyamine and 100 μL MSTFA)	CSF and blood samples from patients with relapsing-remitting multiple sclerosis (*n* = 22) and patients with primary progressive multiple sclerosis (*n* = 12)	103 metabolites in the CSF, and 155 in the serum were identified using NMR; 35 metabolites in the CSF, and 40 in the serum were identified using GC–MS; 66 metabolites (acylcarnitines, glycerophospholipids, sphingolipids, amino acids, and biogenic amines) identified using commercial kits for LC–MS/MS. 49 metabolites (acylcarnitines, glycerophospholipids, sphingolipids) and 12 amino acid and biogenic amines were altered between two groups
[81]	ESI(+) and ESI(−) HPLC–Q–TOF (Kinetex C18 column)	100 μL CSF; protein precipitation (400 μL ACN:MeOH (1:1, *v*/*v*)), drying, and resuspension (100 μL, H_2_O:MeOH (1:1, *v*/*v*))	CSF samples from patients after the initial manifestation of clinical symptoms of multiple sclerosis (*n* = 40) and controls with non-neurological diseases (*n* = 33)	Total 60. Significant differences between groups were observed in arginine, histidine, spermidine, glutamate, choline, tyrosine, serine, methionine, homovanillic, linoleic, oleic, and stearic acids.
[9]	GC–TOF (RTX–5Sil MS column)	100 μL CSF; extraction (650 μL MeOH:isopropanol:H_2_O (3:3:2, *v*/*v*/*v*)), drying, two-step derivatization (5 μL methoxyamine hydrochloride and 45 μL MSTFA)	CSF samples from a multiple sclerosis group (*n* = 54), a neuromyelitis optica spectrum disorder group (*n* = 49), an idiopathic transverse myelitis group (*n* = 30), and control group without CNS pathologies (*n* = 12)	Total 85 from 962 metabolic signatures. Increased: 1-Monopalmitin, 1-monostearin, and glycolic acid. Decreased: glycine, inosine, threose, and butane-2,3-diol in patients with autoimmune diseases in comparison with controls.
Alzheimer’s Disease				
[84]	GC–MS and two variants of LC–MS/MS	Extraction+protein precipitation (MeOH and dichloromethane mixture). For nontargeted LC–MS, both fractions were reconstituted in appropriate suitable solvent mixtures. The non-polar fraction was subjected to acidified MeOH treatment to produce fatty acid methyl esters from both free fatty acids and hydrolyzed complex lipids. Both the non-polar and polar fractions underwent a two-step derivatization (MOX and a silylating agent) before GC–MS analysis. Steroids, catecholamines, and their metabolites were quantified using an online solid-phase extraction–LC–MS/MS.	CSF samples from patients with Alzheimer’s disease (*n* = 79) and controls (*n* = 51)	Total 343. Increased: cortisol and cysteine. Decreased: uridine and 3-methoxy-4-hydroxy phenylglycol.
[85]	ESI(+) and ESI(−) HILIC–HPLC–QQQ (Waters BEH Amide column)	50 μL CSF; protein precipitation (300 μL 80% MeOH in water), drying	CSF samples from patients with mild cognitive impairment (*n* = 9) and controls (*n* = 12)	Total 215. 81 were detected in all CSF samples, including amino acids, nucleotides, lipids, tricarboxylic acid cycle, and vitamins. Increased: 3-hydroxybutyrate after triglyceride infusion
[87]	GC–MS/MS (Phenomenex Zebron ZB-1MS column)	1 mL CSF; aB&D method for lipid extraction and derivatization (50 μL pentafluorobenzyl bromide and 50 μL *NN*-diisopropylethylamine)	CSF samples from patients with Alzheimer’s disease (*n* = 29), patients with mild cognitive impairment (*n* = 30), and controls (*n* = 70)	Total 20. 8 polyunsaturated, 6 monounsaturated, and 6 saturated fatty acids. Decreased: docosahexaenoic acid in the Alzheimer’s disease group compared to controls. Decreased: alpha-linolenic acid in the mild cognitive impairment group compared to controls
[88]	ESI(+) and ESI(-) UPLC–Q–Orbitrap (Accucore C18 column)	400 μL CSF; aB&D method for lipid extraction	CSF samples from patients with Alzheimer’s disease (*n* = 17), patients with mild cognitive impairment (*n* = 15), and controls (*n* = 18)	Total 245. 52 triacylglycerols, 50 phosphatidylcholines, 44 phosphatidylethanolamines, 16 sphingomyelins, 15 phosphatidylinositols, 13 monohexosylceramides, 11 cholesteryl esters, 11 lysophosphatidylcholines, 11 lysophosphatatidylethanolamines, 10 diacylglycerols, 8 ceramides, 2 cyclic phosphatidic acids, and 2 phosphatidylglycerols. 39 lipids were altered in patients with Alzheimer’s disease compared to controls, and 3 lipids were altered in patients with mild cognitive impairment compared to controls.
[89]	ESI(+) and ESI(-) UPLC–Q–TOF	90 µL CSF; MTBE extraction	CSF samples from patients with Alzheimer’s disease (*n* = 91), including those with severe obstructive sleep apnea (*n* = 38)	Total 201. An oxidized ceramide (OxCer(40:6)) and an oxidized triglyceride (OxTG(57:2)) were identified from 11 dysregulated lipid compounds between the observed groups
Parkinson’s Disease				
[91]	GC–MS, two variants of ESI(+) and ESI(−) UPLC–MS/MS	100 µL CSF; organic solvent extraction; for GC–MS: derivatization with BSTFA	CSF samples collected less than 4 h postmortem from pathologically verified Parkinson’s disease patients (*n* = 48) and controls without neurological diseases (*n* = 57)	Total 461. 243 were structurally identified. Increased: 3-hydroxykynurenine, 1,5-anhydroglucitol, N-acetylglycine, sebacate (decanedioate), and also 3-hydroxykynurenine to kynurenic acid ratio. Decreased: *N*-acetyl-β-alanine, *N*-acetylhistidine, succinylcarnitine, trimethylglycine (betaine), 4-acetamidobutanoate, pipecolate, glucose, oxidized glutathione, inosine, *N*-acetylserine, *N*-formylmethionine, cyclo-(leucine-proline), corticosterone, uridine, and 5,6-dihydrothymine
[92]	GC–MS, two variants of ESI(+) and ESI(−) UPLC–MS/MS	The same as above	Plasma and CSF samples collected twice at intervals up to 24 months (baseline and final) from unmedicated and mildly affected Parkinson’s disease patients (*n* = 49)	Total 575 in plasma and 383 in CSF biochemicals were detected, including iminodiacetate, taurine, mannitol, serine, inosine, picolinate, 1-arachidoylglycerophosphocholine (20:0), 1-methylxanthine, 3-hydroxydecanoate, 5-dodecenoate (12:1n7), 5α-androstan-3β-17α-diol-disulfate, docosadienoate (22:2n6), docosatrienoate (22:3n3), ethyl glucuronide, and theobromine in plasma, and benzoate and homovanillate in CSF. CSF concentrations of homovanillate showed little change between baseline and final collections and minimal correlation with Parkinson’s progression
Amyotrophic Lateral Sclerosis				
[97]	GC–TOF (DB 5–MS column)	Extraction (MeOH:H_2_O mixture (8:1, *v*/*v*)), drying, and two-step derivatization (30 μL MOX and 30 μL MSTSA)	CSF samples from sporadic and familial cases of patients with amyotrophic lateral sclerosis (*n* = 39)	Total 120, including amino, fatty, and organic acids; 40 were identified. Decreased: glutamate and glutamine in the familial amyotrophic lateral sclerosis group.
[98]	ESI(+) and ESI(−) UPLC–Q–TOF (Phenomenex Kinetex 1.7 μm XB—C18 and 100 Å HPLC columns)	20 μL CSF; protein precipitation (180 μL MeOH), drying, and resuspension (200 μL ACN/water (1:1, *v*/*v*))	CSF samples from patients with amyotrophic lateral sclerosis (*n* = 66) and patients with other neurological conditions (*n* = 128)	No list of exact compounds
Epilepsy				
[99]	GC–MS/MS	No data	CSF samples from 0 to 5 years group (22 patients with and 13 without epilepsy) and 6–17 years group (12 patients with and 17 without epilepsy)	Total 180. Increased: pyridoxamine and tyrosine, decreased: 2-ketoglutaric acid in the 0–5 years group. Decreased: 1,5-anhydroglucitol in the 6–17 years group.
[100]	GC–MS	No data	CSF samples from epileptic patients (*n* = 23) and non- epileptic patients (*n* = 13)	Total 56. Increased: 36 metabolites, including glycine. Decreased: 20 metabolites, including α-ketoisocaproic acid and xylose
[101]	UPLC–Q–TOF (Acquity UPLC BEH Amide and Acquity UPLC HSS T3 columns)	20 μL CSF; protein precipitation (100 μL MeOH), 90 μL of the supernatant was collected, combined with 250 μL of pre-cooled MTBE, and then centrifuged. The lower layer was analyzed.	CSF samples from pediatric patients with unfavorable outcome status epilepticus (*n* = 13), favorable outcome status epilepticus (*n* = 15), and non-status epilepticus controls (*n* = 9)	Total 221. Increased: (2′,6′)-7-methyl-3-methylene-1,2,6,7-octanetetrol, hydroxyprolyl-proline, hydantoin-5-propionic acid, and glycerophosphocholine, decreased: 12 metabolites, including triacetin, betaine aldehyde, and gamma- aminobutyryl- lysine in pediatric patients with favorable outcomes in status epilepticus compared to controls. Increased: 23 metabolites, including hydantoin- 5- propionic acid, citrate, and carbamoyl phosphate, decreased: l-fucose, diethylphosphate, cytidine, betaine aldehyde, and triacetin in pediatric patients with unfavorable outcomes in status epilepticus compared to controls. Increased: 44 metabolites, including glutamyl- glutamine, lysyl- glutamine, 3- iodothyronamine, and uridine, decreased: diethylphosphate, l-fucose, varanic acid, taurine, and succinate in the unfavorable outcome group compared to favorable ones
Oncological Diseases				
[102]	GC–MS (DB–5 column)	50 μL CSF; extraction (250 μL MeOH:H_2_O:CHCl, 2.5:1:1), drying, and two-step derivatization (40 μL MOX and 20 μL MSTSA)	CSF samples from patients with on histologically confirmed glioma (10 patients with I-II grade, 8 patients with III grade, and 12 patients with IV grade)	Total 61. 45 were identified, including malic, aconitic, succinic, fumaric, isocitric, and citric acids, leucine, isoleucine, alanine, valine, proline, serine, threonine, methionine, phenylalanine, and tyrosine. Increased: citric and isocitric acids in patients with glioblastoma (grade IV gliomas) compared to other groups.
[103]	ESI(+) and ESI(−) HILIC–UPLC–Q–TOF	250 μL CSF; precipitation (80% MeOH), drying, and resuspension (50 μL H_2_O/ACN)	CSF samples from malignant glioma patients (4 had newly diagnosed and 6 had recurrent disease) and controls (*n* = 7)	Total 124, including biotin, dihydroorotate, glucono-D-lactone, acetylcarnitine, aminoadipic acid, proline, phenyllactic acid, phenylpropiolic acid, *N*^6^-acetyl-l-lysine, oxaloacetate, acetyllysine, indole-3-carboxylic acid, shikimate, atrolactic acid, 2,3-dihydroxybenzoic acid, methionine, taurine, orotate, purine, *N*-acetyl-glutamine, 2-ketohaxanoic acid, lysine, glucosamine, adenine, nicotinamide, thiamine, phenylalanine, S-methyl-5-thioadenosine, serine, 7-methylguanosine, glutamine, hypoxanthine, 2-hydroxy-2-methylbutanedioic acid, ribose-phosphate, myo-inositol, isocitrate, and glucose-1-phosphate. Newly diagnosed patients clustered into a different subtype and showed low levels of tryptophan metabolites
[104]	UPLC–Q–Orbitrap (C18–pfp column)	50 µL CSF; metabolomics: protein precipitation (80% MeOH), drying, and resuspension (0.1% formic acid in H_2_O); lipidomics: aFolch extraction	CSF samples from pediatric patients with medulloblastoma (*n* = 40) and pediatric controls without any cancer (*n* = 11)	Total 352. Increased: tricarboxylic acid cycle and other metabolites (succinate, malate, hydroxypyruvate, fumarate, α-ketoglutarate, *N*-acetyl-aspartate) and total triacylglycerols. Decreased: citrate, isocitrate, trans-aconitate, gamma-aminobutyric acid, diacylglycerols (*n* = 17), monogalactosyldiacylglycerols (*n* = 19), cholesterol esters (*n* = 14), phosphatidylcholines (*n* = 85), *N*-hexadecanoyl hexosylceramides (*n* = 6), sphingomyelins (*n* = 51), and oxidized lipids
[105]	GC–MS, ESI(+) and ESI(−) HILIC–UPLC–MS/MS and RP–UPLC–MS/MS	150 µL CSF; protein precipitation (MeOH), and the extract fractions were subjected to HILIC–UPLC–MS/MS and RP–UPLC–MS/MS	CSF samples from pediatric patients with acute lymphoblastic leukemia divided into a discovery (*n* = 86) and replication (*n* = 85) groups	Total 313 in the discovery cohort. Increased: dimethylglycine, allantoin, ribitol, and dimethylmalonic acid. Decreased: of gamma-glutamylglutamine, 3-methoxytyrosine, asparagine, and myoinositol. Total 409 in the replication group, with 274 overlapping between both cohorts.
[106]	UPLC–Q–TOF	The same as above	Plasma, CSF, and marrow samples from pediatric patients with acute lymphoblastic leukemia (*n* = 10)	Total 816 in plasma, 774 in marrow, and 366 in CSF. Pyruvate and asparagine showed positive correlations between plasma and bone marrow. Positive plasma-CSF correlation for dimethylglycine and negative plasma-CSF correlation for gamma-glutamylglutamine were observed.
[107]	ESI(+) LC–Q–TOF (Zorbax Explise Plus C18 column)	Extraction (MeOH), drying, and resuspension (50% ACN)	CSF samples from patients with primary (*n* = 59) and secondary CNS lymphomas (*n* = 11), lung carcinoma patients with (*n* = 34) and without brain metastases (*n* = 23), nontumorous brain disease controls (*n* = 36)	Total 508. 27 metabolites can distinguish among various brain tumor types. Phytosphingosine, cystine, 5-aminoimidazole, glutamine, and butyrylcarnitine differed between groups of primary CNS lymphoma and nontumorous brain diseases. Phytosphingosine, dehydroascorbic acid, 5-aminoimidazole, 1-methyladenosine, prolyl-threonine, and cystine differed between lung adenocarcinoma patients with brain metastases and those with nontumorous brain diseases. Inositol phosphate, 5-aminoimidazole, homocysteine, and valyl-methionine were different in groups of primary and secondary CNS lymphomas. In addition, differences in metabolite content were observed between patients with primary or secondary CNS lymphoma and those with lung adenocarcinoma with or without brain metastases.
[108]	ESI(+) and ESI(−) LC–MS/MS	10 µL CSF, extraction (240 µL ACN:MeOH:H_2_O (3:5:2 *v*/*v*))	CSF samples from patients with non-malignant disease (*n* = 31), including normal (*n*= 12), benign tumor (*n* = 3), inflammation disease (*n* = 6), demyelinating disease (*n* = 10); metastatic cancer (*n* = 30), including lung cancer (*n* = 12), breast cancer (*n* =7), glioma (*n* = 7), other cancer (*n* = 4)	No list of exact compounds
Neuroinflammatory Diseases				
[109]	ESI(+) and ESI(−) UPLC–Q–TOF (ACQUITY HSS T3)	100 µL CSF, extraction+protein precipitation (400 µL MeOH/ACN (1:1, *v*/*v*)), drying, and resuspension (100 µL H_2_O/ACN (5:5, *v*/*v*)	CSF samples from patients with neurosyphilis (*n* = 18), non-neurosyphilis (*n* = 18), and syphilis-free (*n* = 18)	Total 1808. l-gulono-gamma-lactone, d-mannose, *N*-acetyl-l-tyrosine, and hypoxanthine separated all groups
[110]	ESI(+) and ESI(−) LC–Orbitrap (Hypergod C18 column)	100 μL CSF; extraction (300 μL MeOH)	CSF samples from patients with neurosyphilis (*n* = 16) and non-neurosyphilis (*n* = 14)	1179 features in the ESI(+) and 1363 in ESI(−). 40 metabolites were capable of distinguishing non-neurosyphilis from controls. 6 metabolites (alpha-kamlolenic acid, l-histidine, bilirubin, prostaglandin E2, palmitoyl-l-carnitine, and butyryl-l-carnitine) were proposed as biomarkers of neurosyphilis
[111]	HPLC–QQQ	30 μL CSF; AbsoluteIDQ^®^-p180 kit (Biocrates Life Science AG, Innsbruck, Austria)	CSF samples from patients with three manifestations of varicella zoster virus reactivation (segmental zoster (*n* = 14), facial nerve zoster (*n* = 16), and zoster meningitis (*n* = 15) and/or encephalitis), enteroviral meningitis (*n* = 10), idiopathic Bell’s palsy (*n* = 11), and normal pressure hydrocephalus (*n* = 15)	21 amino acids and 21 biogenic amines detected by liquid chromatography separation followed by targeted tandem mass spectrometry (MS/MS) and 91 glycerophospholipids (phosphatidyl- and lysophosphatidylcholines) and isomers, 40 acylcarnitines, 15 sphingolipids (sphingo- and hydroxysphingomyelins) and isomers, and the sum of hexoses were detected by direct infusion MS/MS (flow injection analysis). 88 metabolites comprised 28% of acylcarnitines, 86% of amino acids, 24% of biogenic amines, 46% of glycerophospholipids, 80% of sphingolipids, and the sum of hexoses distinguished all three forms of varicella zoster virus reactivation from other samples, with four metabolites, including glycine, associated with meningoencephalitis
[112]	HPLC–QQQ	The same as above	CSF samples from patients with enteroviral meningitis (*n* = 10), non-inflamed patients with idiopathic Bell’s palsy and normal pressure hydrocephalus (*n* = 19)	Total 91. 18 amino acids, 7 biogenic amines, 11 acylcarnitines, 43 glycerophospholipids, 11 sphingolipids, and the sum of hexoses. Asparagine, glycine, and especially kynurenine were the best biomarkers for enteroviral meningitis
[114]	ESI(+) UPLC–Q–Orbitrap (Agilent Infinity Lab HILIC column)	100 μL CSF; extraction+protein precipitation (300 µL MeOH), drying, and resuspension (50 µL H_2_O/ACN (4:6, *v*/*v*)	CSF samples from patients with acute encephalitis (acute disseminated encephalomyelitis *n* = 6, unknown suspected viral encephalitis *n* = 3, enteroviral encephalitis *n* = 2, seronegative autoimmune encephalitis *n* = 2, herpes simplex encephalitis *n* = 1) and age-matched non-inflammatory neurological disease controls (*n* = 14)	Total 35. Increased: kynurenine, quinolinic acid, and anthranilic acid in patients with encephalitis, decreased: tryptophan, 3-hydroxyanthrnailic acid, and kynurenic acid. Increased: asymmetric dimethylarginine and argininosuccinic acid, decreased: arginine and citrulline in patients with encephalitis. An increase in the CSF kynurenine/tryptophan ratio (*p* < 0.001), anthranilic acid/3-hydroxyanthranilic acid ratio (*p* < 0.001), asymmetric dimethylarginine/arginine ratio (*p* < 0.001), and neopterin (*p* < 0.001) strongly predicted neuroinflammation.
[119]	ESI(+) and ESI(−) UPLC–Q–TOF	150 μL CSF; extraction (300 μL MeOH)	CSF samples from patients with tuberculous meningitis (*n* = 50), caused by *Mycobacterium tuberculosis*, compared to viral (*n* = 17), bacterial (*n* = 17), and cryptococcal meningitis (*n* = 16)	Total 1161 in the ESI(+) and 512 in the ESI(−). 13 metabolites separated between tuberculous and viral meningitis, 16 between tuberculous and bacterial meningitis, and 9 between tuberculous and cryptococcal meningitis with most of the metabolites increased in tuberculous meningitis. Most of the metabolites exhibited increased contents in the CSF of TBM patients compared to other types of meningitis, except for lower 3,4-dihydroxybenzoate concentration in TBM than viral meningitis, decreased level of chenodeoxycholate and phosphatidic acid (PA 18:1/0:0) in TBM than bacterial meningitis
[121]	HPLC–Q–Orbitrap (Atlantis HILIC column 130)	10 μL CSF; extraction (90 μL ACN/MeOH/formic acid (74.9:24.9:0.2 *v*/*v*/*v*)	CSF samples from patients with tuberculous meningitis (*n* = 1067)	Total 619. Increased: 433 metabolites, decreased: 36 metabolites in TBM patients than non-infectious controls. 10 associated with 60-day mortality, including tryptophan, 4-hydroxyphenylacetic acid, phenyllactic acid, and some hydroxylated fatty acids
[123]	LC–MS/MS, GC–MS	50–100 μL CSF. No exact data	CSF samples from infants with (*n* = 19) and without bacterial meningitis (*n* = 19)	Total 422. Increased: 196 metabolites. Decreased: 14 metabolites. Cytidine, ornithine, proline, glutamate, taurine, 2-hydroxyglutarate, thymine, N6-acetyllysine, and α-ketoglutarate were the most important to separate patients with AUC = 0.97. Increased: α-Hydroxyisocaproic and 2-hydroxy-3-methylvaleric acids, decreased: sucrose in bacterial meningitis caused by Streptococcus agalactiae compared to all other cases with bacterial meningitis
[124]	ESI(+) and ESI(−) HPLC–Q–Orbitrap (Acquity UPLC HSS T3 column)	No data	CSF samples from infants with neonatal sepsis (*n* = 70), including with meningoencephalitis (*n* = 42)	Total 91. Increased: pyridoxal, kynurenic acid, homovanillic acid, pyrrolidine, pyruvic acid, L-proline, dopamine, phenolglyoxylic acid, and glycocholic acid. Decreased: homo-L-arginine, urea, phosphoric acid, and creatinine, compared to group without meningoencephalitis.
“Secondary” and Undiagnosed Conditions Affecting the Nervous System				
[129]	LC–MS/MS	No access to Supplementary Materials	CSF samples from patients with symptoms of hepatic encephalopathy (*n* = 14) and controls (*n* = 27)	Total 122. 73 metabolites altered, including amino acids, bile acids, acylcarnitines, and nucleosides
[130]	ESI(+) and ESI(−) UPLC–Q–TOF (Acquity BEH C18 column)	50 μL CSF; protein precipitation (400 μL ACN + 0.1% formic acid), resuspension (400 μL 50% MeOH + 0.1% formic acid)	CSF samples from patients with type2 diabetes mellitus (*n* = 28) and controls (*n* = 25)	Increased tryptophan in new-onset disease compared to controls with altered uric acid and paraxanthine in the current disease group
[126]	ESI(+) and ESI(−) UPLC–Orbitrap (Acquity UPLC HSS T3 column)	100 μL CSF; protein precipitation (300 μL MeOH)	CSF samples from neuro-PASC patients (*n* = 21), healthy volunteers (*n* = 45), and inflammatory neurological diseases patients (*n* = 26)	Total 93. Increased: 60 metabolites. Decreased: 10 compared to controls
[131]	ESI UPLC–MS/MS, GC–MS	Extraction, drying. For UPLC–MS/MS: resuspension in acidic (eluted using water and MeOH both containing 0.1% formic acid) or basic conditions (eluted using water and MeOH containing 6.5mM ammonium bicarbonate). For GC–MS: derivatization (BSTFA)	CSF samples from HIV-positive patients (*n* = 46) and HIV-negative controls (*n* = 54)	Total 200. 104 identified, including amino acids (49%), lipids (16%), nucleotides (8%), and others (27%): glutamate, *N*-acetylaspartate, *N*-acetylaspartylglutamic acid, glycine, dopamine, serotonin, GABA, choline, myoinositol, arachidonate, 5-oxoproline and homocarnosine, lactate, creatinine, phenylacetylglutamine, p-cresol sulfate; 4-hydroxyphenyllactic acid
[132]	GC–MS and UPLC–Q–Orbitrap	200 μL CSF; aFolsh method	Patients with undiagnosed diseases	Total 82 CSF metabolites, 81 plasma polar metabolites, and 116 urine metabolites

**Table 2:** Comparative overview of bioanalytical method validation parameters according to FDA [27], EMA [28], and ICH M10 [29].

Parameter	General Requirements According to ICH M10 (2023) [29]	FDA (2018) [27]	EMA (2012) [28]
Linearity assessed by analyzing calibration standards covering the expected concentration range	≥6 non-zero levels; ≥75% within ±15% (±20% at LLOQ); weighted regression; inter-run reproducibility	Flexible weighting; focus on reproducibility	Emphasis on back-calculated concentrations
LLOQ/ULOQ lower and upper limits of quantification	LLOQ: ±20%, CV ≤ 20%; ULOQ: ±15%, CV ≤ 15%	Performance-based approach	Includes S/N considerations (≥5)
Selectivity evaluates interference from endogenous matrix components	≥6 matrix sources; interference ≤ 20% (analyte), ≤5% (IS) compared to LLOQ	Standard requirement	Same as the FDA
Specificity ability to distinguish analyte from structurally related compounds	Differentiation from metabolites/interferences; interference ≤ 20% (analyte), ≤5% (IS) compared to LLOQ	Often included in selectivity	Explicit focus on metabolites
Sensitivity primarily defined by the LLOQ	Defined by LLOQ performance	Accuracy/precision-based	Includes signal/noise expectations
Matrix effect evaluated to assess ion suppression or enhancement	≥6 matrix sources; QC at 2 levels in 3 replicates; accuracy ±15%; CV ≤ 15%	Less prescriptive	Detailed experimental approach
Carryover	≤20% (analyte), ≤5% (IS) compared to LLOQ	Harmonized	Harmonized
Accuracy reflects closeness to nominal concentration	QC at ≥4 levels; ±15% (±20% LLOQ)	Harmonized	Harmonized
Precision expressed as the CV	Intra-/inter-run; CV ≤ 15% (≤20% LLOQ)	Harmonized	Harmonized
Recovery evaluates extraction efficiency	Pre-spiked samples compared to post-spiked samples; consistent and reproducible	Detailed experimental approach	Less prescriptive
Stability ensure analyte integrity under various conditions *	±15% deviation; multiple conditions required	Flexible	More explicit conditions

* Bench-top (short-term) stability; long-term storage stability; freeze–thaw stability; processed sample stability (autosampler stability; under the storage conditions); stock and working solution stability.

**Table 3:** The brief description of the studies devoted to the targeted CSF metabolomics.

Metabolite Class	Platform	CSF Volume (µL)	Derivatization	Guidelines Followed	Representative Analytes (Concentration Range in CSF)	Example References
Neurotransmitters & metabolites (HVA, 5-HIAA, GABA, etc.)	UPLC–QQQ, HPLC–QQQ	1–100	Benzoyl chloride, propyl chloroformate, or none	FDA, CLSI, EMA	HVA 248–584 nM; 5-HIAA 112–215 nM; GABA 7–344 nM	[144,145,146,147,150,152]
Tryptophan–kynurenine pathway	UPLC–QQQ, HPLC–QQQ	10–100	None (most methods)	FDA, EMA	Kynurenine 55 nM; quinolinic acid 48 nM; tryptophan 2.2–3.1 µM	[115,149,158,159]
Catecholamines & metabolites	UPLC–QQQ, GC–MS	50–500	Propyl chloroformate, dansyl chloride, or none	FDA, CLSI, EMA	Dopamine 0.1–0.4 nM; norepinephrine 0.3–0.7 nM; epinephrine < 0.1 nM	[147,149,152,153]
Aromatic microbial metabolites	UPLC–QQQ, GC–MS	40–200	Silylation (BSTFA)	FDA, ICH M10	4-Hydroxyphenyllactic acid 0.8–17 µM; 3-phenyllactic acid < 0.4–2.9 µM	[172,173]
Amino acids (broad panel)	GC–MS, GC–TOF	100–250	Methoxyamine + BSTFA/MSTFA	None specified	Glycine 5–15 µM; alanine 6–20 µM; proline 4–10 µM	[167,168]
Endocannabinoids	micro–LC–Q–Trap	250	None	EMA	Anandamide 1–7 pM; 2-arachidonoyl glycerol88–659 pM	[197]
Oxysterols & bile acids	UPLC–Q–Trap, 2D–HPLC–Q–Trap	55–200	Pentafluorobenzyl bromide, nicotinic acid/EDC	FDA, EMA	24S-Hydroxycholesterol (0.025–5 ng/mL)	[200,201]
Neurosteroids	GC–MS, GC–MS/MS, UPLC–QQQ	1000–2000	Methoxyamine, dansyl chloride	None specified	Pregnenolone 19–53 pg/mL; dehydroepiandrosterone 60–300 pM; allopregnanolone 6–44 pg/mL	[204,205,208]
Vitamin B6 vitamers	UPLC–QQQ	50–60	None	ICH M10, FDA	Pyridoxal 15–42 nM; pyridoxal 5′-phosphate 8–42 nM	[212,213]

## Data Availability

All data is in Supplementary Materials.

## Supplementary Materials

The following supporting information can be downloaded at: https://www.mdpi.com/article/10.3390/molecules31111822/s1, Table S1. A brief description of the studies devoted to the targeted CSF metabolomics.

## Abbreviations

The following abbreviations are used in this manuscript:

aB&D	Acidified Bligh and Dyer
B&D	Bligh and Dyer
CNS	Central nervous system
CSF	Cerebrospinal fluid
CV	Coefficient of variation
DOPAC	3,4-Dihydroxyphenylacetic acid
EMA	the European Medicines Agency
ESI	Electrospray ionization
FDA	the U.S. Food and Drug Administration
GABA	γ-Aminobutyric acid
GC-MS	Gas chromatography–mass spectrometry
GWAS	Genome-wide association studies
5-HIAA	5-Hydroxyindole-3-acetic acid
HILIC	Hydrophilic interaction liquid chromatography
HPLC/UPLC-MS	High-performance/ultra-high-performance liquid chromatography–mass spectrometry
HVA	Homovanillic acid
ICH M10	the International Council for Harmonization
LLOQ	Lower limit of quantification
LLE	Liquid–liquid extraction
LOD	Limit of detection
MHPG	3-Methoxy-4-hydroxyphenylglycol
MS	Mass spectrometry
MSI	the Metabolomics Standards Initiative
MS/MS	Tandem mass spectrometry
MWAS	Metabolome-wide association study
NMR	Nuclear magnetic resonance
PCR	Polymerase chain reaction
RP	Reversed-phase
TOF	Time-of-flight
Q	Quadrupole
QC	Quality control
QQQ	Triple-quadrupole
ULOQ	Upper limit of quantification
