# Supplementary material for: Untargeted and Targeted Cerebrospinal Fluid Neurometabolomics via Chromatography–Mass Spectrometry-Based Methods
Source: Molecules. 2026 May 25;31(11):1822. doi: 10.3390/molecules31111822 (PMC13257592; doi:10.3390/molecules31111822)
Supplement: Supplementary file 1 [file molecules-31-01822-s001.zip › molecules-4285018-supplementary.pdf]

Supplementary Table S1. The brief description of the studies devoted to the targeted CSF metabolomics.

Ref.	Compounds	Analytical Method	Sample preparation	Sample	Method validation	Concentrations	Commentary
Neurotransmitters							
141	Gamma-hydroxybutiric acid (GHB)	GC-MS: 30 m × 0.25 mm × 0.25 µm, VF-5 ms	50 µL CSF; extraction (200 µL 0.1M HCl + 1 mL ethyl acetate), drying, derivatization (50 µL MeCN + 25 µL BSTFA+1% TMCS)	From autopsy cases (n = 21) in which death had been concluded to be caused by circumstances other than drug-related	Inter- and intra-day accuracy: ≥91%<br>Imprecision: ≤9%<br>LOD: 0.5 mg/L<br>LOQ: 0.6 mg/L<br>Cal. curve: 1.0 mg/L, 10 mg/L, 40 mg/L, 80 mg/L and 100 mg/L	Range(mean|median) concentrations in CSF after immediate analysis and analysis after storage for 14 days at 4°C and 20°C, mg/L: 1.1–10.4 (4.2|3.8)/0.6–13.2 (4.3|3.3)/<0.5–21.6 (4.8|4.3), ~after storage for 30 days at 4°C and 20°C, mg/L: 1.1–10.4 (4.5|4.0)/<0.5–6.5 (3.3|3.6)/<0.5–21.2 (4.5|2.8)	Post-mortem GHB changes seem to be affected both during post-mortem interval in the dead body and during in vitro storage. There seem to be inter-individual differences: While some samples showed high increases in GHB concentrations, some samples showed no or very small changes in GHB concentrations

142	Gamma-aminobutiric acid (GABA)	Isotope-dilution GC-MS: 25 m × 0.32 mm, CPSil 88 column	Free GABA: 500 µL CSF + 100 µL aqueous 2 µM IS + 800 µL 1M phosphate buffer, pH 11.5 + 50 µL methylchloroformate + 150 µL 6M HCl + 4 mL ethyl acetate + drying + 100 µL 7% pentafluorobenzylbromide in MeCN + 10 µL triethylamine +150 µL 0.5M HCl + 1 mL hexane + drying + 50 µL hexane. Final volume 2 µL. Total GABA: 50 µL CSF + 450 µL water + 100 µL 2 µM aqueous IS + 250 µL 20% sulphosalicylic acid + hydrolysis 24 h at 110°C. After hydrolysis the subsequent sample preparation procedure was the same as for free GABA determination	CSF samples of a patient before and during Vigabatrin treatment, control samples	LOD: < 0.005 µM<br>Free GABA<br>Intra-assay: 0.188 ± 0.004 µM (1.9% SD)<br>Inter-assay: 0.177 ± 0.013 µM (7.3% SD)<br>Total GABA<br>Intra-assay: 3.00 ± 0.05 µM (1.8% SD)<br>Inter-assay: 3.57 ± 0.33 µM (9.2% SD)	Free/total GABA, µM<br>Control: 0.029-0.127/4.72-11.8<br>Before therapy: 0.153/13.2<br>During therapy: 0.274/24.1	

144	Aspartic acid (Asp), serine (Ser), glycine (Gly), glutamic acid (Glu), γ-aminobutyric acid (GABA), norepinephrine (NE), epinephrine (EP), dopamine (DA), acetylcholine (ACh), serotonin (5HT), histamine (His), and 1-methylhistamine (MHis)	ESI (+) UPLC-QQQ, ACE Excel 2 C18-AR (150 × 2.1 mm) column. Eluent system: A = 0.1% HCOOH, 2.5 mM nonafluoropentanoic acid in water and B = ACN. A linear gradient (1–50% B in 6 min), a flow rate of 0.5 mL/min. Total run-time 6 min	No sample preparation of 1 µL CSF, just dilution	For validation: artificial CSF (150 mM Na+, 3 mM K+, 1.4 mM Ca++, 0.8 mM Mg++, 1 mM H2PO4−, 155 mM Cl−, 10 mM glucose, 0.5 mg mL−1 bovine serum albumin). For real sample analysis: mice CSF (n=37)	FDA guidelines. Lin. Asp, Ser, Gly, 1-100 µM; other 0.1-100 µM (R2 >0.97); Intra-day and inter-day accuracy (RE) <14%; Intra-day and inter-day precision (CV) <15%. Internal Standards: glutamate-2,3,3,4,4-d5, γ-aminobutiric acid-2,2,3,3,4,4-d6, histamine-α,α,β,β-d4	Mean concentrations (µL): Aspartic acid (Asp) - 10, serine (Ser) - 40, glycine (Gly) - 12, glutamic acid (Glu) - 36, γ-aminobutyric acid (GABA) - 7, norepinephrine (NE) - 0,3, epinephrine (EP) < LLOQ, dopamine (DA) < LLOQ, acetylcholine (ACh) < LLOQ, serotonin (5HT) - 0.4, histamine (His) - 0.2, and 1-methylhistamine (MHis) - 0.16	No results of matrix effect and recovery, short-term and long-tern stabilities, and freeze–thaw cycles assessment
145	Glutamine, glutamate, pyroglutamate, and GABA	ESI (+) HPLC-QQQ, Phenomenex Synergi 4 µm Polar-RP 80A, 150 × 4.6 mm column. Eluent system: A = 1% formic acid and 0.5% HFBA in water. B = 1% formic acid and 0.5% HFBA in ACN. The initial mobile phase composition was 100%mobile phase A and was ramped in a linear fashion to 5% mobile phase B in 4.0 min. The total run time 5.0 min	50 µL CSF; dilution in 10 mM ammonium acetate	For validation: saline solution (0.9% sodium chloride). For real sample analysis: CSF from rat (n=10) and humans (n=6)	Lin. 7.8-2000 ng/mL. Intra-day and inter-day accuracy -22<(RE) <10%; Intra-day and inter-day precision (CV) <23%. The stock solution proved to be stable for a period of 12 days refrigerated at 5 ◦C. The analytes were shown to be stable at room temperature in proxy CSF at concentrations of 31 and 1000 ng/ml for 24 h. 2 freeze–thaw cycles were assessed. Internal standards: l-glutamic 2,3,3,4,4-d5 acid, aminobutyric-2,2,3,3,4,4-d6 acid, l-Glutamine-2,3,3,4,4-d5, and 2,3,3,4,4-d5 pyroglutamic acid	Human CFS: glutamine (57,700 ± 3995 ng/ml), while pyroglutamate (17,167 ± 1180 ng/ml), glutamate (1357 ± 50 ng/ml), and GABA (89 ± 6 ng/ml). In rat CSF, glutamine (16,790 ± 1154 ng/ml), glutamate (3864 ± 1540 ng/ml), pyroglutamate (685 ± 53 ng/ml), and GABA (344 ± 183 ng/ml)	Since the article does not specify the requirements according to which the protocol was validated, the authors considered the following validation parameters to be satisfactory: for pyroglutamate accuracy (RE, %) at 2000 ng/mL -22.3 and precision (CV, %) at 7.8 ng/mL 23.1.
146	Serotonin, 5HIAA, HVA	ESI (+) for serotonin, serotonin-d4, 5-HIAA, and 5-HIAA-d2; ESI (-) for HVA and HVA-d3	100 µL CSF; protein precipitation (100 µL MeOH containing 0.2 % FA); drying, and	For validation: Phosphate-buffered saline solution. For real sample analysis:	CLSI Document C62-A guidelines. Lin. Serotonin 0.5-500 ng/mL, 5HIAA 0.2-100 ng/mL, HVA 2-1000	Serotonin < LLOQ; 5HIAA 14.6 vs. 8.6 ng/mL; HVA 45 vs. 24 ng/mL	In the CSF, no significant ion suppression was observed except 5-

		UPLC-Q-Trap, ACQUITY UPLC HSS T3 column (2.1 × 100 mm, 1.8 µm). Eluent system: A = H_2O/0.01 % FA/10 mM ammonium formate and B = 90 % acetonitrile/10 % H_2O/0.01 % FA/10 mM ammonium formate at a flow rate of 0.4 mL/min. Gradient: 0–0.50 min: 3 % B; 0.50–4.00 min: 3–38 % B; 4.00–4.01 min: 38–98 % B; 4.01–5.00 min: 98 % B; 5.00–5.10 min: 98–3 % B; 5.10–6.50 min: 3 % B. Total run-time 6.5 min	resuspension (50 µL water)	CSF from patients with (n=11) and without (n=15) motor impairment	ng/mL (R^2 >0.998). Recoveries serotonin 81.5-112.4 %, 5-HIAA 80.3- 113.8 %, and HVA 84.8-105.0 %. Intra-day and inter-day precision (CV) ≤10%. No carry-over effect. For stability assessment 5-HIAA and HVA remained relatively stable in serum, CSF, and urine within 7 days at 4 ◦C and room temperature, while serum serotonin slightly decreased at room temperature, but remained stable at 4 ◦C. The neurotransmitters remained stable in 4–5 freeze–thaw cycles. Internal Standards: serotonin-d4 hydrochloride, 5HIAA-d2, HVA-d3		HIAA. The IS-corrected ratios ranged from 85.71 to 101.27 % for serotonin, 85.06 to 114.79 % for 5-HIAA, and 93.75 to 114.61 % for HVA. In addition, the 100-fold dilution of urine with water makes the matrix effect of serotonin and HVA insignificant. Both no-corrected and IS-corrected ratios were within the range of 85–115 %, although mild ion suppression of 5HIAA could be observed in some samples and could then be corrected with IS

147	3-orthomethyldopa, 5-hydroxytryptophan, 5HIAA, and HVA	ESI (+) UPLC-QQQ, Acquity UPLC BEH Shield RP18 column (2.1 × 100 mm 1.7 µm). Eluent system: A = 1 mmol/L ammonium formate in water, 0.05 % formic acid and B = 1 mmol/L ammonium formate in methanol, 0.05 % formic acid. A linear gradient elution was at 0.25 mL/min: from 0.0 min to 0.5 min: 95 % A –5 % B; from 0.5 min to 2.5 min: 0 % A − 100 % B; from 2.5 min to 6.0 min: 95 % A – 5 % B. Total run-time 6 min.	50 µL CSF; protein precipitation (140 µL 0.01 % perchloric acid)	For validation: aCSF; saline, and Ringer-lactate solution. For real sample analysis: CSF from pediatric patients (n=10) suspected for neurometabolic disorders	FDA and ICH 10 guidelines. Lin. (nM): 3-orthomethyldopa 2.5-500, 5-hydroxytryptophan 2.5-500, 5HIAA 25--2500, and HVA 25-2500 (R2 >0.999). Intra-day and inter-day accuracy and precision ≤13%. Recovery 101-108% with CV < 13%. Matrix factor Assessed stability at 48 h at autosampler, 12 months at -70C, bench top 4h at 24 C, and 3 freeze–thaw cycles. Internal standard: vanillic acid	Range (nM): 3-orthomethyldopa 8.3-65.1, 5-hydroxytryptophan 5.0-14.5, 5HIAA 104-397, and HVA 237-856	All analytes showed ion enhancement (MF > 100 %) in low, medium and high QC levels in aCSF and saline solution. 3-OMD also showed this phenomenon for the three QC levels in Ringer-lactate. In this matrix, ion suppression (MF < 100 %) was found for 5-HTRP, 5-HIAA and HVA. Matrix effect was also evaluated for the IS. In aCSF and Ringer-lactate solution, MF for VA was > 100 %, which is indicative of ion enhancement. IS-norm MF was calculated for each analyte, on each QC level and matrix. In aCSF IS-norm MF was near 1.000 for 3-OMD, 5-HTRP, 5-HIAA and HVA. For this reason, it was selected as the appropriate surrogate matrix.

148	Acetylcholine (ACh), histamine (HA), tele-methylhistamine (t-mHA), and tele-methylimidazolacetic acid (t-MIAA)	ESI (+) UPLC-QQQ, KinetexTM HILIC Core–Shell HPLC column (2.1 × 100 mm). Eluent system: A = 0.2% formic acid and 20 mM ammonium formate in water. B = 100% ACN. The initial conditions 90% B, and a linear gradient: A increasing from 10 to 70% within 1.25 min. Total run-time 4 min	50 µL CSF; protein precipitation (80 µL ACN)	For validation: aCSF, rat, monkey, and human CSF	Lin.(ng/mL): ACh 0.025–5; HA, t-mHA, and t-MIAA 0.05–10 (R2 >0.99). The intra-run precision and accuracy for all analytes were 1.9–12.3% CV and −10.2 to 9.4% RE, respectively, inter-run precision and accuracy were 4.0–16.0% CV and −5.3 to 13.4% RE, respectively. The stabilities of ACh, HA, t-mHA, and t-MIAA in stock solution and in the final extract were also evaluated. When prepared in the stock solution at a concentration of 1 mg/mL, the neat standards of all analytes were determined to be stable for up to 6 h at room temperature and for at least 1 month at −20 ◦C. Internal standards ACh-1,1,2,2-d4, d3-t-mHA, and d3-t-MIAA	In rat CSF (ng/mL): ACh 0.12 ± 0.04, HA 0.77 ± 0.27, t-mHA, 0.69 ± 0.21, and t-MIAA 1.45 ± 0.29	Validation guidelines were not clarified. No recovery and matrix effect assessment. Without an acetylcholinesterase (AChE) inhibitor present, ACh was found to have 1.9 ± 0.4 min in vitro half-life in rat CSF. Stability studies and processing modification, including the use of AChE inhibitor eserine, extended this half life to more than 60 min.

149	Tyrosine, levodopa, 3-O-methyldopa, dopamine, 3-methoxytyramine, 3,4-dihydroxyphenylacetic acid, HVA, vanillylmandelic acid, and 3-methoxy-4-hydroxyphenylglycol sulphate; tryptophan, L-kynurenine, kynurenic acid, 3-hydroxyanthranilic acid, 3-hydroxykynurenine, xanthurenic acid, anthranilic acid, quinolinic acid, cinnabaric acid, tryptamine, indoleacetic acid, 5-hydroxytryptophan, serotonin, 5HIAA and melatonine, neopterin, biopterin, dihydrobiopterin, cortisol, and histamine	ESI (+) and ESI (-) UPLC-QQQ; Waters Atlantis dC18 (2.1 mm x 150 mm, 5 µm) column. Eluent system: A = water + 0.2% FA. B = ACN + 0.2% formic acid. Gradient: 0 – 1 min, 3% B; 1 – 3.3 min, 3% B – 10% B; 3.3 – 8.3 min, 10%B – 35% B; 8.3 – 9.3 min, 35% B –95% B; 9.3 – 13.3 min, 95% B; 13.3 – 15.3 min, 95% B – 3% B; 15.3 min – 20 min 3% B. Flow rate 0.6 mL/min. Total run-time 20 min	30 µL CSF; protein precipitation (320 µL MeOH/FA (99.8/0.2), drying, resuspension (60 µL DS).	For real sample analysis: CSF from patients (n=10) with neurological disorders (patients with cephalalgia, astrocytoma, lumbago, dementia or infarctus cerebri, 30–48 years old)	EMA guidelines. Lin. see article ($R2$ >0.998). Intra-day and inter-day accuracy were between 80-119% and 81-117%, precision 1-19% respectively. Stock solutions, serum and CSF samples were stable for at least 24h at 4°C and 3 months at -20°C and -80C. Prepared serum and CSF samples were stable in the autosampler (10°C) at least for 24h. Stability tests after 3 freeze–thaw cycles showed no decomposition. Internal standards: DA-d4, TYR-d4, DOPA-d3, KA-d5, CRNd3, CORT-d4, QA-d3, and 5-HT-d4	TYR 9948 ± 3537<br>DOPA 9.2 ± 5.5<br>3-O-MD 22.5 ± 8.9<br>DA 1.2 ± 0.4<br>3-MT 1.5 ± 1<br>DOPAC 3.5 ± 2.3<br>HVA 248.9 ± 124.0<br>VMA <2<br>MHPGS <1<br>TRP 2205 ± 510<br>KYN 59.1 ± 27.4<br>3-OHK 4.7 ± 2.9<br>XA 1.3 ± 1.0<br>KA 2.4 ± 1.6<br>AA 4.7 ± 2.1<br>3-OHAA <1<br>CIA n.d.<br>QA 48.0 ± 29.9<br>TPA <2<br>IAA 130.5 ± 77.3<br>MEL 0.10 ± 0.02<br>5-HTP 8.4 ± 1.5<br>5-HT <0.5<br>5-HIAA 112.8 ± 73.6<br>NEO 9.2 ± 4.3<br>BIO 5.3 ± 4.3<br>BH2 17.7 ± 6.8<br>CORT 42.1 ± 18.4<br>MNA 39.5 ± 25.5<br>HA 76.3 ± 15.7	

150	Serotonin, 5HIAA, HVA, noradrenaline (NADR), adrenaline (ADR), dopamine (DA), glutamic acid (Glu), γ-aminobutyric acid (GABA), 3,4-dihydroxyphenylacetic acid (DOPAC), and histamine (His)	ESI (+) UPLC-QQQ, BEH C18 column (2.1 x 150 mm, 1.7 µm). Eluent system: A = 10 mM ammonium formate/0.15% formic acid in water, B = ACN. Gradient: 10% B for 2 min; increased to 90% B from 2 min to 10 min; 90% B from 10 min to 11 min, and then 10% B from 11 min to 13.3 min; the flow rate of 0.3 mL/min. Total run-time 13.3 min	50 µL CSF; protein precipitation (50 µl ACN), derivatization (25 µl borate buffer (sodium tetraborate, 100 mM) and 25 µl benzoyl chloride (2% in ACN, v/v))	For validation: artificial CSF (145 mM NaCl, 2.68 mM KCl, 1.1 mM MgSO4, 1.22 mM CaCl2, 0.5 mM NaH2PO4, 1.55 mM Na2HPO4 and 0.5 mg/ml BSA; pH was adjusted to 7.4). For real sample analysis: CSF from rats with model for human tauopathy (n=19) and controls (n=9)	Lin. (ng/mL): Serotonin 0.15-50, 5HIAA 5-200, HVA 5-200, noradrenaline (NADR) 0.2-50, adrenaline (ADR) 0.07-50, dopamine (DA) 0.15-100, glutamic acid (Glu) 100-4000, γ-aminobutyric acid (GABA) 4-800, 3,4-dihydroxyphenylacetic acid (DOPAC) 1-200, and histamine (His) 0.12-50 (R2 >0.98). Intra-day and inter-day accuracy (CV) ≤12%. There were no significant differences in response for Glu, GABA, HVA, DOPAC, SER, NADR, ADR and DA stored for 24 h at RT (peak area 94.6–118.7%). Derivates of HIS and 5-HIAA were stable for up to 4 h at RT (peak area 112.5–116.1%). All derivates were stable for at least 24 h when stored at 4 C (autosampler condition) or at 80 C (peak area 96.3–111.5%). Internal standards: 13C6-benzoyl chloride, D6-4-aminobutyric acid	Mean concentrations (ng/mL): Serotonin 0.45, 5HIAA 101, HVA 16, noradrenaline (NADR) 0.8, adrenaline (ADR) no exact data (on fig.), dopamine (DA) no exact data (on fig.), glutamic acid (Glu) 614, γ-aminobutyric acid (GABA) 35, 3,4-dihydroxyphenylacetic acid (DOPAC) 10, and histamine (His) 1.7.	Based on the extraction recoveries for DOPA, NADR, ADR, HVA, 5-HIAA, DOPAC, HIS and SER any matrix effect was excluded for these compounds. Glu and GABA showed ion enhancement effect. To overcome this problem, deuterated IS for Glu and GABA were used for analysis. The application of deuterated IS significantly reduced matrix effect for GABA (extraction recovery 90.275%) and was used for further validations and analysis of samples from animals. The use of deuterated IS for Glu did not cause reduction in matrix effects and 13C6 benzoylated IS was used for further Glu analysis. No substantial changes in neurotransmitters and metabolites in CSF of transgenic animals (early and late stage of neurodegeneration). Only ADR (mean 0.35 ng/ml in CTRL vs 0.98 ng/ml in SHR72 TS) and 5-HIAA (mean 101 ng/ml in CTRL vs 139 ng/ml in SHR72 TS) were statistically significantly elevated in transgenic animals.

151	DOMA 3,4-Dihydroxymandelic acid, DOPAC 3,4-Dihydroxyphenylacetic acid, LDOPA 3,4-Dihydro-xyphenylalanine, DOPEG 3,4-Dihydroxyphenylglycol, 3HAA 3-Hydroxyanthranilic acid, 3HK 3-Hydroxykynurenine, MOPEG 3-Methoxy-4-hydroxyphenylglycol, 3MT 3-Methoxytyramine, GABA 4-Aminobutyric acid, 5HIAA 5-Hydroxyindoleacetic acid, 5HTP 5-Hydroxytryptophan, 5HTOL 5-Hydroxytryptophol, ACh Acetylcholine, Ado Adenosine, Agm Agmatine Ala Alanine Ans Anserine Arg Arginine Asn Asparagine Asp Aspartate BAla b-Alanine Carn Carnosine Ch Choline Cit Citrulline CA Cysteic acid Cys Cysteine DA Dopamine E Epinephrine ETA Ethanolamine Glc Glucose	ESI (+) UPLC–QQQ, Acquity HSS T3C18 column (1 mm × 100 mm, 1.8 µm). Eluent system: A = 10 mM ammonium formate with 0.15% formic acid, B = ACN. Gradient: initial, 0% B; 0.01 min, 15% B; 0.5 min, 17% B; 14 min, 55% B; 14.5 min, 70% B; 18 min, 100% B; 19 min, 100% B; 19.1 min, 0% B; and 20 min, 0% B. The flow rate 100 mL/min. Total run time 33 min	Samples were diluted 100-fold in water, and a 10 µL aliquot was derivatized (5 µL 100 mM sodium carbonate, 5 µL benzoyl chloride (2% (v/v) in ACN)	For validation: artificial CSF (145 mM NaCl, 2.68 mM KCl, 1.0 mM $MgSO_4$, 1.4 mM $CaCl_2$, 0.45 mM NaH_2PO_4, 1.55 mM Na_2HPO_4 and 0.5 mg/ml BSA; pH was adjusted to 7.4). For real sample analysis: pooled human CSF from healthy patients	LODs: <10 nM except for glutathione, alanine, citrulline, glycine, serine, and glucose; carryover <4%; RSD < 7%, R^2>0.98. Internal Standards: d4-ACh and d4-Ch	35 compounds > LOD in human CSF	Not fully validated

	Glu Glutamate<br>Gln Glutamine<br>GSH Glutathione<br>Gly Glycine<br>Hist Histamine<br>His Histidine<br>HCA Homocysteic acid, HCY Homocysteine,<br>HSer Homoserine<br>HVA Homovanillic acid, HTau Hypotaurine<br>KA Kynurenic acid<br>Kyn Kynurenine<br>Kyo Kyotorphin<br>Leu Leucine<br>Lys Lysine<br>Met Methionine<br>NAP N-Acetylputrescine<br>NAS N-Acetylserotonin<br>NE Norepinephrine<br>NM Normetanephrine<br>OA Octopamine<br>Orn Ornithine<br>Phe Phenylalanine<br>PhEt Phenylethylamine<br>Pro Proline<br>Put Putrescine<br>Ser Serine<br>5HT Serotonin<br>Spd Spermidine<br>Spm Spermine Syn Synephrine<br>Tau Taurine<br>Thr Threonine<br>TrpA Tryptamine<br>Trp Tryptophan<br>TyrA Tyramine<br>Tyr Tyrosine						

	Val Valine<br>VMA<br>Vanillylmandelic acid							
152	DA, dopamine; DOPAC, dihydroxyphenylacetic acid; E, epinephrine; 5-HIAA; 5-HTP, 5-hydroxytryptophan; HVA; MHPG, 3-methoxy-4-hydroxyphenylglycol; 3-MT, 3-methoxytyramine; VMA, vanillylmandelic acid; VLA, vanillactic acid; 5-HT, 5-hydroxytryptamine; MN, metanephrine; NMN, normetanephrine; NE, norepinephrine; 3-OMD, 3-O-methyl-dopa; L-DOPA	ESI (+) UPLC–QQQ, Acquity UPLC BEH Phenyl column (100 × 2.1 mm, 1.7 µm). Eluent system: A = ACN–water (1:4, v:v) containing 1.0 mmol/L ammonium acetate and 0.1% acetic acid. B = ACN. Flow rate 0.2 mL/min. Gradient: 20% B maintained for 1.0 min, increased to 60% at 3.0 min, and increased to 70% at 6.0 min, then decreased to 20% at 6.1 min followed by 3.0 min for equilibration.	50 µL CSF; dilution (50 µL of 20 mmol/L sodium tetraborate), derivatization (40 µL of freshly prepared 3-picoline/n-propanol solution (23:77, v/v) and 25 µL of propyl chloroformate /isooctane /dichloromethane mixture (17:11:72, v/v/v)), extraction (200 µL of ethyl acetate), drying, resuspension (100 µL MeOH/water (1:1, v/v))	For validation: KPO4 buffer. For real sample analysis: CSF samples from patients (n=16) and controls (n=200)	EMA guidelines. All analytes were detectable in concentrations between 0.01 and 0.10 nmol/L and quantifiable in concentrations between 0.02 and 0.50 nmol/L, except MHPG, which was quantifiable at 1.60 nmol/L. The absolute recoveries ranged from 57.5 % to 125.9 % for low levels of analytes, from 61.8 % to 107.4 % for medium levels of analytes, and from 66.5 % to 103.6 % for high levels of analytes The absolute MF values for analytes and ISs ranged from 29.5 % to 92.9 %. The IS-normalized MF values ranged from 87.4 % to 118.7 %. The intra- and inter-day imprecision were < 15% for most analytes, and accuracy ranged from 90.3% to 111.6%. The stability study showed that standard stock solutions were stable at − 80 ◦C for six years when prepared in the protection solutions; Analytes in CSF samples were stable for 24 h on wet ice and at least two years at − 80 ◦C; repeated freeze–thaw should be avoided. Internal Standards: deuterated analogs of all analytes	The sensitive method enabled accurate quantification of biomarkers in the entire reference intervals, except 3-MT, DA, MN, and 5-HT, which had levels lower than LOQs in some CSF samples. DA 0.10 ± 0.07 (<0.27), L-DOPA 4.99 ± 2.60 (1.47–12.03), DOPAC 6.95 ± 3.09 (2.26–15.69), E 0.03 ± 0.03 (<0.11), 5HIAA 215.28 ± 58.48 (132.62–369.71), 5-HT 0.21 ± 0.20 (<0.78), 5-HTP 8.25 ± 1.55 (5.85–11.44), HVA 584.4 ± 182.25 (292.25–1051.00), MHPG 55.91 ± 15.68 (34.41–105.70), MN 0.09 ± 0.07 (<0.26), 3-MT 0.21 ± 0.12 (<0.55) NMN 0.72 ± 0.33 (0.29–1.71), NE 0.70 ± 0.36 (0.22–1.60),3- OMD 29.96 ± 14.06 (8.36–65.45), VLA 1.08 ± 0.61 (0.22–2.80), VMA 0.25 ± 0.12 (0.10–0.57)	16 CSF samples from patients with THD, AADCD, autosomal dominant GTPCHD, or GRIN2B gene mutations, were recruited in this study.

153	Dopamine (DA), epinephrine (E), norepinephrine (NE), metanephrine (MN), normetanephrine (NMN), and 3-methoxytyramine (3-MT)	ESI (+) UPLC–QQQ, Kinetex F5 column (100 × 3 mm, 2.6 µm). Eluent system: A = 0.1 % formic acid in water. B= MeOH. Total run time 6.5 min.	500 µL CSF; dilution (500 µL of buffer solution (0.2 mmol/L ammonium acetate)); 1000 µL of the mixture was transferred to the activated SPE plate. Formic acid (5 % in methanol, 200 µL) was added for elution; drying; resuspension (60 µL of 0.1 % formic acid)	For validation: aCSF. For real sample analysis: CSF samples from patients with Alzheimer's disease (n=8) and normal cognition controls (n=8)	CLSI Document C62-A guidelines. Lin. DA and NE 4.5–3600 pg/mL, E and MN at concentrations of 2.5–2000 pg/mL, NMN at concentrations of 2–1600 pg/mL, and 3-MT at concentrations of 0.3–240 pg/mL in plasma and CSF (R2 > 0.99). intra- and inter-assay precision (CV) <12%. No carry-over effect. For matrix effect after IS correction the minimum was 85.71 % and maximum was 110.98 %. Assessment of storage at room temperature for 2 h, − 20 ◦C for 3 days, and − 80 ◦C for 7 days. Internal Standards: DA-d4, E-d6, NE-d3, MN-d3, NMN-d3, 3-MT-d4	Concentration (pg/mL) AD vs controls: DA 17.16 ±7.39 vs 35.13 ±26.55; E 6.53 ± 7.8 vs. 2.24 ±1.05; NE 279.21 ± 76.8 vs. 159.62 ± 69.73; MN 2.56 ± 1.2 vs. 3.04 ±1.89; NMN 112.34 ± 63.74 vs. 81.91 ± 33.8, 3-MT 7.27 ±4.68 vs. 5.48 ± 2.3	The concentrations of DA, E, NE, MN, NMN, and 3-MT in CSF samples in the present study were similar to those in previous studies

115	Neopterin, tryptophan, kynurenine, kynurenic acid, 3-hydroxykynurenine, xanthurenic acid, anthranilic acid, 3-hydroxyanthranilic acid, quinolinic acid, picolinic acid, arginine, citrulline and methylhistamine	ESI (+) UPLC–QQQ, Acquity UPLC BEH C18 column (2.1 x 150 mm, 1.7 µm). Eluent system: A = 10 mM ammonium formate and 0.1 % formic acid in water B = 0.1 % formic acid in ACN. Gradient: 0 to 2.5 min (0 % B), 2.5 to 4.5 min (0-10 % B), 4.5 to 6.5 min (10-30 % B), 6.5 to 8 min (30-35% B), 8 to 8.5 min (35- 100 % B), 8.5 to 8.6 min(100-0 % B) and 8.6 to 12 min (0 % B). Flow rate 0.25 mL/min. Total run time 12 min	The analysis of tryptophan, kynurenine, kynurenic acid, xanthurenic acid, 3-hydroxyanthranilic acid and quinolinic acid: 80 µL CSF; protein precipitation (20 µL MPA/EDTA solution). The analysis of neopterin, 3-hydroxykynurenine, anthranilic acid, picolinic acid, arginine, citrulline and methylhistamine: 80 µL CSF; dilution	For validation: charcoal stripped pooled human CSF. For real sample analysis: CSF samples from patients with acute encephalitis (n=10; myelin oligodendrocyte glycoprotein encephalitis n=3, anti-N-methyl-D-aspartate encephalitis n=2, acute disseminated encephalomyelitis n=2, herpes simplex encephalitis n=1, enteroviral encephalitis n=1) and frequency-matched non-inflammatory neurological disease controls (n=10)	Lin.: from LOQs 0.75-3.00 ng/mL to 1000 ng/mL, good linearity ($R2 > 0.98$), matrix effects (from - 19.4 to 14.9%); recoveries (89.8-109.1 %). There were no interferences observed from common endogenous CSF metabolites, no carryover. The accuracy (RE) from -13,5 to 15% and precision (CV) < 15 %. All analytes in matrix-matched pooled human CSF calibrators and human CSF extracts were stable for 24 h after extraction and two freeze–thaw cycles. Internal standards: D-3-kynurenic acid, 15N-neopterin, D3-tryptophan, D4-kynurenine, 13C2-3-hydroxykynurenine, D3-3-hydroxyanthranilic acid, 13C6-arginine and D7-citrulline	Concentration (nmol/l) only on Fig. 4.	Two different sample preparation protocols were employed.Validation guidelines : ISO 15189 Medical laboratories - Requirements for quality and competence. 3 ed Geneva, Switzerland: International Organization for Standardization; 2012. ISO/TS 20914 Medical laboratories Practical guidance for the estimation of measurement uncertainty. Geneva, Switzerland: International Organization for Standardization; 2019. NATA. General Accreditation Guidance Validation and verification of quantitative and qualitative test methods. Rhodes, Australia: National Association of Testing Authorities; 2018. elevated kynurenine/tryptophan ratio ($p<0.001$), quinolinic acid/kynurenic ratio ($p<0.01$), and anthranilic acid/3-hydroxyanthranilic acid ratio ($p<0.01$) were observed during acute neuroinflammation.

159	L-tryptophan (L-TRP), kynurenic acid (KA), L-kynurenine (KYN), 3-hydroxy-L-kynurenine (3-OH-KYN), serotonin (5-HT), melatonin (M), N1-acetyl-N2-formyl-5-methoxykynuramine (AFMK), tryptamine (TA), indole-3-acetic acid (IAA), anthranilic acid (AA), 3-hydroxy-anthranilic acid (3-OH-AA), 5HIAA, N-methylserotonin (N-Me-5-HT), 5-hydroxy-L-tryptophan (5-OH-L-TRP), N-methyltryptamine (N-Me-TA), 5-methoxytryptamine (5-MeOTA), N-acetylserotonin (N-Ac-5-HT)	ESI (+) and ESI (-) UPLC–QQQ, Acquity UPLC HSS T3Column (100 Å, 1.8 μm, 2.1 × 100 mm). Gradient of 0 – 2 min 98:2 A:B (v/v) (isocratic elution) followed by 2–10 min 40:60 A:B (v/v) (gradient elution) where A was 0.1% formic acid and B was methanol. Flow rate 0.3 mL/min. Total run time 10 min	100 µL CSF; protein precipitation (390 µL MeOH), drying, resuspension (30 µL 2% MeOH)	For real sample analysis: CSF samples from healthy donors (n=18)	FDA and EMA guidelines. Lin: from 0.3-45 to 1500 nmol/L; Recovery 22.1 to 370.0% for CSF; accuracy 90.7 to 127.7% for CSF matrix; All intra- and inter-day coefficients of variation were below 15%. Accuracy 90.7- 127.7%. Various stability assessments. Internal Standards: [13C215N1]-3-OH-KYN and deuterated analogs of rest of analytes	Concentrations (median): L-tryptophan (L-TRP) 3.10 µM , kynurenic acid (KA) 1.70 nM, L-kynurenine (KYN) 55.70 µM, 3-hydroxy-L-kynurenine (3-OH-KYN) 1.10 nM, serotonin (5-HT) <0.30 nM, melatonin (M) <0.30 nM, N1-acetyl-N2-formyl-5-methoxykynuramine (AFMK) <0.30 nM, tryptamine (TA) <2.30 nM, indole-3-acetic acid (IAA) 27.60 µM, anthranilic acid (AA) 4.70 nM, 3-hydroxyanthranilic acid (3-OH-AA) <2.30 nM, 5HIAA 38.30 nM, N-methylserotonin (N-Me-5-HT) <0.30 nM, 5-hydroxy-L-tryptophan (5-OH-L-TRP) 10.20 nM, N-methyltryptamine (N-Me-TA) <0.30 nM, 5-methoxytryptamine (5-MeOTA) <0.75 nM, N-acetylserotonin (N-Ac-5-HT) <6.00 nM	Not satisfactory validation and calibration ranges

158	Tryptophan, kynurenic acid, kynurenine, xanthurenic acid, anthranilic acid, quinolinic acid, 3-hydroxykynurenine, picolinic acid, 3-hydroxyanthranilic acid	ESI (+) HPLC-QQQ ZORBAX Eclipse-XDB C-8 column (3.0 mm × 150 mm, 5 µm) Eluent system: A = 0.2% (v/v) formic acid in ACN/H2O (1: 99 v/v) and B = 0.2% formic acid in MeOH. Linear gradient elution with 5% B to 46.25% B from 0 to 11 min (3.75 % B/min). Flow rate 0.6 mL/min	60 µL CSF; no sample preparation	For real sample analysis: CSF samples from donors (n=17)	FDA guidelines. Lin. (nM): Tryptophan 1-9919, kynurenic acid 0.8-8596, kynurenine 1-9985, xanthurenic acid 1-9919, anthranilic acid 1-2680, quinolinic acid 3.6-1011, 3-hydroxykynurenine 1-9919, picolinic acid 3.5-1000, 3-hydroxyanthranilic acid 1-9919 (R2 > 0.99). Matrix effect 1-27%, accuracy 84-118%, precision <4%. No carryover, various stabilities assessed, stable after 5 freeze/thraw cycles. Internal Standards: stable isotopic-labeled standards for all analytes	Concentration (nM) only on Fig. 6.	
161	5-hydroxyindole ethanol (5-HTOL), 5-hydroxyindole acetic acid (5-HIAA), 5-hydroxytryptophan (5-HTP) and 5-hydroxytryptamine (5-HT)	GC-MS: 30 m × 0.25 mm × 0.25 µm, DB-5 column	3 mL CSF; SPE: washing 2 mL distilled water; elution 1 mL MeOH + 0.5% formic acid; drying; derivatization (70 µL BSTFA+1% TMCS + 30 µL pyridine + 2.5 µL methanol agent)	For real sample analysis: Newly diagnosed acute lymphoblastic leukemia children without chemotherapy (n = 36), children without tumors of the nervous system and infection for control group (n = 24)	Matrix effect: 92.3–106.2% (no significant ME) Lin. 0.5–200.0 µg/L (5-HTOL, 5-HIAA, R2≥0.9924) and 2.0–800.0 µg/L (5-HTP, 5-HT, R2≥0.9918) LOD: 0.1–0.4 µg/L LOQ: 0.5 (5-HTOL, 5-HIAA) and 2.0 (5-HTP, 5-HT) µg/L Intra-day recovery: 94.6–105.6% (CV 1.4–4.5%) Inter-day recovery: 93.0–106.9% (CV 1.8–4.5%)	Acute lymphoblastic leukemia: 4.3/61.0/5.3/3.8 Normal: 4.5/88.9/5.8/6.5	5-HT and 5-HIAA contents in the acute lymphoblastic leukemia group were significantly lower than those in the control group (p < 0.01), while there was no significant difference in 5-HTP and 5-HTOL contents between the two groups (p > 0.05).

162	Quinolinic acid (QUIN), picolinic acid (PIC), nicotinic acid (NIC)	GC-MS: 30 m × 0.25 mm, HP–5MS capillary columns with either (i) 0.25 µm or (ii) 1.0 µm stationary-phase film thickness; electron-capture negative ionization mode with methane as reagent gas	CSF + 50 µL IS mix + evaporation to dryness + 100 µL trifluoroacetic anhydride + 100 µL hexafluoroisopropanol + heating at 60°C for 30 min/leaving at room temperature overnight + dissolving in 1 mL toluene + washing with 1 mL 5% $NaHCO_3$ + 1 mL water + ~500 mg anhydrous Na_2SO_4. For the hydrolysis of NAM samples + IS + drying under reduced pressure + 200 µL HCl + 5% phenol + evacuation and flushing with N_2 × 3 + incubation 18 h at 110°C	For validation: aCSF. For real sample analysis: Human CSF samples.	On-column LOQ: < 1 fmol (S/N 10:1)<br>Lin.: 0–5 pmol on column<br>Slope: for NIC 5.8; for PIC 25.8; for NAM 3.9 ($R2>0.996$)<br>Precision (RSD): 0.5–4.3%<br>Accuracy: 94.0–105.5%<br>Inter-day precision: 1.0–8.9%<br>Inter-day accuracy: 96.7–104.0%	NIC: 2.0 (prehidrolysis) and 56.2 (after hydrolysis) µM nicotinamide: 54.2 µM (quantified from the pre- and posthydrolysis NIC)	In unhydrolyzed sample endogenous PIC and QUIN are present but there is only a trace of NIC. Following hydrolysis of a human CSF sample, there is a 28-fold increase in measured NIC deriving from endogenous NAM in this CSF sample. Paper Lim et. al. Kynurenine pathway metabolomics predicts and provides mechanistic insight into multiple sclerosis progression. 2017 https://doi.org/10.1038/srep41473 has the reference to this method.
Amino acids							
167	Valine, alanine, sarcosine, leucine, isoleucine, benzoic acid, glycine, serine, threonine, methionine, aspartic acid, phenylalanine, phenylglycine, hippuric acid, caffeine, theophylline, lysine, tyrosine, uric acid, 5-hydroxyindole-3-acetic, nortriptyline	GC-MS<br>30 m × 0.25 mm × 0.25 µm, HP-5-MS column<br>APCI: positive mode, temperature and flow rate of the dry gas (nitrogen) 250°C and 5.00 L/min; vaporizer temperature 450°C; the pressure of the nebulizer gas (nitrogen) 2 bar<br>microTOF (Bruker Daltonik, Bremen, Germany)	250 µL CSF; protein precipitation (600 µL MeOH); drying; derivatization (100 µL methoxyamine·HCl in pyridine + 100 µL BSTFA/MSTFA + 1% TMCS)	Stock standard solutions (200 µM) of the 31 compounds in methanol.<br>Human CSF samples	LOD: 11.8–72.5 nM<br>LOQ: 39.3–241.7 nM<br>Lin.: LOQ – 100 µM<br>R2: 0.902–0.987<br>Intra-day (RSD): 0.7–2.1%<br>Inter-day (RSD): 3.8–6.4%<br>Reproducibility (RSD): ≤8.9%	No quantitative data	The ratio of pyridine/methoxyamine was chosen 1:1 because regardless of the derivatization reagent (BSTFA or MSTFA) changing the ratio did not affect peak areas of the test mixture significantly. To reduce the error and shorten time, 30 min was selected as derivatization time. The influence of temperature on peak areas was minimal (in the evaluated interval between room

							temperature and 80°C), however at 40°C more compounds with just one TMS derivative were observed.
168	Glycine, sarcosine, L-forms: alanine, valine, leucine, isoleucine, serine, threonine, methionine, aspartic acid, proline, cysteine, glutamic acid, phenylalanine, asparagine, lysine	GC-MS: Rtx-5MS (30 m × 0.25 mm × 0.25 µm), containing 5% diphenyl + 95% dimethylpolysiloxane	200 µL CSF; protein precipitation (800 µL MeOH); drying; derivatization (15 µL methoxyamine in pyridine; 35 µL BSTFA+TMCS (99:1 v/v))	For validation: aCSF (127 µM NaCl + 2 µM KCl + 1.2 µM KH2PO4 + 26 µM NaHCO3 + 2 µM MgSO4 + 2 µM CaCl2 + 10 µM 4-(2-hydroxyethyl)-1-piperazineethanesulfonic acid (HEPES) + 10 mM glucose + bubbled with O2:CO2, v/v 95:5). For real sample analysis: CSF samples from patients with acquired immunodeficiency syndrome (AIDS), suspected of having bacterial infection (cryptococcal meningitis) (n=16)	Recovery: 88–129%<br>LOD: 0.01–4.24 µM<br>LOQ: 0.02–7.07 µM<br>Intra-day (RSD): 4.1–15.6%<br>Inter-day (RSD): 6.4–18.7%<br>Lin. 0.1–133.0 µM (R2=0.99 for amino acids except cysteine)	Median (n = 16), µM: 6.9/4.9/19.9/9.3/6.7/4.2/7.9/7.6/10.4/6.2/375.0/1046.7/13.0/4.8/1.1/10.4	All 16 amino acids were detected
170	Arginine, asymmetric dimethylarginine (ADMA), and symmetric dimethylarginine (SDMA)	ESI (+) HPLC-QQQ, Macherey-Nagel Nucleosil 100-5 125 mm × 3 mm. Isocratic elution with a mobile phase consisting of 100 ml water, 900 ml acetonitrile, 0.25 ml TFA and 10 ml propionic acid at a flow rate of 0.5 ml/min	100 µL CSF; 900 µL a mixture of ACN/trifluoroacetic acid (TFA)/propionic acid 100/0.025/1 (v/v)	For real sample analysis: CSF samples from patients after subarachnoid hemorrhage (n=15)	Lin. 1.5–60 µM Arg, 0.03–1.0 µM ADMA, 0.04–1.3 µM SDMA. Precision and accuracy < 5%. Internal standards: 13C6-arginine, D6-ADMA	On the day of admission: 19.4 ± 5.5 µM for Arg, 0.068 ± 0.021 µM for ADMA and 0.396 ± 0.196 µM for SDMA	These calibration ranges cover the CSF concentrations observed in healthy volunteers and patients suffering from SAH. The CSF values for Arg and ADMA were not statistically different from the controls (19.7 ± 7.1 and 0.060 ± 0.008 µM, respectively)

171	Homocysteine and homocysteic acid	ESI (+) UPLC–QQQ, Ascentis Express C8 column (100 mm × 4.6 mm, 2.7-µm. A= 10 mM ammonium formate aqueous solution and B= methanol	Before in-tube SPME, CSF samples were diluted and treated with DTT reduction agent to cleave disulfide bonds and to release Hcy. 150 µL of CSF was diluted with 90 µL of 30 mM ammonium acetate buffer (pH 9.0), 20 µL of 1 M DTT in water, 20 µL of ISs, and 20nµL of water (or standard solution for calibrators), which made up 300 µL. 300 µL of treated CSF sample was percolated through the capillary by using 30 mM ammonium acetate buffer (pH 9.0) at 0.1 mL min− 1 for extraction. Elution was carried out by injecting 300 µL of water containing 5 % formic acid. Between each analysis, the capillary was washed with 300 µL of water containing 5 % formic acid. The eluted sample was dried in a concentrator plus for 1.5 h and resuspended in 50 µL of mobile phase.	For validation: 234 mM sucrose, 3.6 mM KCl, 1.2 mM MgCl2, 2.5 mM CaCl2, 1.2 mM NaH2PO4, 12 mM glucose, 25 mM NaHCO3, and 0.15 % bovine serum albumin in water. For real sample analysis: CSF samples from patients diagnosed with AD (n = 12); patients with mild cognitive impairment (n = 11); and age-matched healthy volunteers (n = 15)	FDA guidelines. Linear from 8 to 250 ng mL− 1 Hcy and from 5 to 150 ng mL− 1 HCA (R2>0.99). Precision and accuracy were within the acceptance criteria, with intra and inter-assay CVs ranging from 2.1 % to 14.9 % for Hcy and from 4.1 % to 10.6 % for HCA, and intra- and inter-assay RSD between − 12.7 % and 17. % and between − 2.6 % and 3.7 % for Hcy and HCA, respectively. no significant matrix effect. internal standards DL-Hcy-d4 and DL-HCAd4	The mean Hcy concentration was 31.98 ng mL− 1 in AD patients, 36.38 ng mL− 1 in MCI patients, and 24.11 ng mL− 1 in healthy controls. HCA was not detected in any of the analyzed samples	

172	Benzoic acid (BA), phenylpropionic acid (PhPA), phenyllactic acid (PhLA), 4-hydroxybenzoic acid (p-HBA), 4-hydroxyphenylacetic acid (p-HPhAA), 4-hydroxyphenylpropionic acid (p-HPhPA), homovanillic acid (HVA), 4-hydroxyphenyllactic acid (p-HPhLA)	GC-MS: 30 m × 0.25 mm × 0.25 µm, TR-5ms	MEPS: 40 µL CSF + 10 µL aqueous solution of IS (4 mg/L) + 40 µL distilled water, pH 7 + MEPS + conditioning with MeOH + distilled water + 0.3 mM solution of formic acid + 3 × 50 µL, 900 µL/min + loading + 20 × 50 µL, 300 µL/min + washing with 0.3 mM formic acid + 2 × 20 µL, 500 µL/min + drying air + 12 × 50 µL, 900 µL/min + elution with diethyl ether + 10 × 10 µL, 300 µL/min + complete drying + 80 µL BSTFA + incubation 30 min at 90°C + cooling 30 min at 4°C + 350 µL hexane<br>LLE: 200 µL CSF + 5 µL aqueous solution of IS (7.5 mg/L) + 800 µL distilled water + 0.3–0.5 g solid NaCl + 15 µL concentrated sulfuric acid + diethyl ether + extraction 2 × 1 mL + evaporation at 40°C + derivatization as for MEPS	For validation: pooled CSF. The residues of CSF samples (n = 138) from neurosurgical patients (n = 84) with different pathology (neoplasms of brain and other parts of CNS (n = 62), intracranial injury (n = 5), cerebrovascular diseases of CNS (n = 7), stroke (n = 8), meningitis (n = 2)). Pooled CSF samples were prepared for validation by mixing samples from several patients with negative microbiological culture results for these CSF samples	Recovery: 40-90%<br>LOD: 0.1-0.3 µM<br>LOQ: 0.4-0.7 µM<br>Precision (the reproducibility, RSD): <20%<br>Accuracy (the relative error, RE): <±20%<br>Lin.: over 0.4-10 µM ($R2 \geq 0.99$)	Different concentrations in patients. Median (BA/PhPA/PhLA/p-HBA/p-HPhAA/HVA/p-HPhLA), µM: 0.7/<LOQ/0.1/nd/<LOQ/0.3/0.7/2.5, Range (min-max), µM: <LOQ-11.4/nd - <LOQ/nd -2.9/nd - 0.6/nd -2.3/nd - 6.4/<LOQ-14.9/0.8-17.3	4-Hydroxyphenyllactic, benzoic, 3-phenyllactic, HVA, 4-hydroxybenzoic, and 4-hydroxyphenylacetic acids were quantified in more than a half of CSF samples from neurosurgical patients with suspected secondary meningitis
173	5-HIAA, indole-3-carboxylic, indole-3-acetic, indole-3-propionic, indole-3-lactic, 3-phenylpropionic, 3-	ESI (-) and ESI (+) UPLC-QQQ, Waters Acquity UPLC HSS C18 (50 mm × 2.1 mm, 1.7 µm). A (1 % acetic acid in water) and B (1 % acetic acid	100 µL CSF; protein precipitation (400 µL MeOH), drying, resuspension (100 µL water)	For validation: deionized water. For real sample analysis: CSF samples (n = 29) from post-	FDA and ICH guidelines. Lin. from 2 nM to 3750 nM; $r2 > 0.99$. The accuracy of the method for determination of the analytes in CSF ranged from 88 % to 111 %,	Median (nM): p-HPhLA 949; p-HBA 30; p-HPhAA 103; PhPA <25; p-HPhPA <7.5; PhLA 82; 5HIAA 178; 3ILA 33;	All analytes, except PhPA, p-HPhPA, and 3IPA, were measured in all CSF samples; 3IPA was measured in 20 from 29 samples (69

	phenyllactic; 4-hydroxybenzoic, 4-hydroxyphenylpropionic, 4-hydroxyphenylacetic, and 4-hydroxyphenyllactic acids	in ACN). Gradient: 5 % B from 0.00 to 2.50 min; 5–45 % B from 2.50 to 6.50 min; 45–100 % B from 6.50 to 6.55 min; 100 % B from 6.55 to 7.50 min; 100–5 % B from 7.50 to 7.55 min; 5 % B from 7.55 to 9.00 min. The total run time 9 min. Flow rate 0.4 mL/min.		neurosurgical patients	and CV ranged from 1.1 % to 11 %. Mean recovery values of the analytes at LQC, MQC, and HQC levels in serum were close to 100 %, and CV was 1.8–7.2 %. Normalized matrix factor values were from 0.95 to 0.97 at the LQC level (CV 2.8–8.9 %), 1.0 at the MQC level (CV 3.1–3.3 %) and from 0.90 to 1.0 at HQC level (CV 1.3–5.2 %)	3ICA 7; 3IAA 71; 3IPA 6.	%); PhPA and p-HPhPA were measured only in 2 and 1 samples, respectively
180	Free (FSA) and total (TSA) sialic acid	ESI (-) HPLC-MS/MS	FSA: 75 µL CSF; protein precipitation (300 µL MeOH); drying; resuspension (90 µL water). TSA: 15 µL CSF; protein precipitation and deconjugation (60 µL sulphuric acid (63 mmol/L))	Real sample analysis: CSF from control subjects (n= 217), patients with meningitis (n = 6), brain tumour (n = 2), leukaemia (n = 5), and Salla disease (n = 1)	Limit of detection (LOD) was 0.54 µM for FSA and 0.45 µM for TSA. Intra- and inter-assay variation for FSA (21.8 µM) were 4.8% (n = 10) and 10.4% (n = 40) respectively. Intra- and inter-assay variation for TSA (35.6 µM) were 9.7% (n = 10) and 12.8% (n = 40) respectively. Accuracy of FSA was 98.8% and 97.3%for TSA. LOD was 0.6 µM and LOQ was 2.0 µM for FSA as well as for TSA. The assay was found to be linear till 6402 µM with a correlation coefficient (r2) of 0.999. Recovery of FSA was 94–101% and 91–105% for TSA. No effect of freeze–thaw cycles (n = 10) was found. Internal Standard: 1,2,3-13C3 N-acetyld-neuraminic acid	Control subjects (µM): free - 11.8 ± 4.2; total - 28.4 ± 9.2; and conjugated 16.6 ± 6.0	Both patients with a brain tumour showed increased concentrations of TSA and CSA and only one patient had normal FSA content in CSF. The patient suffering from Salla disease showed increased levels of FSA, TSA and CSA levels in CSF compared with reference values

181	Guanidinoacetate (GAA), creatine (Cr)	Stable isotope dilution GC-MS: SGE BPX-70	100 µL CSF + 50 µL NaHCO3 + 50 µL hexafluoroacetylacetone + 500 µL toluene + 50 µL IS + heating 2 h to 80°C + cooling + 300 µL toluene phase + blowing to dryness under N2 + 10 µL triethylamine + 100 µL 7% pentafluorobenzylbromide in MeCN (v/v) + incubation 15 min at room temperature + 200 µL 0.5N HCl +1 mL hexane + extraction	8 GAMT-deficient and 8 SLC6A8-deficient patients	Linearity: 0.5-10 nmol and 0.05-0.5 nmol LOD (S/N = 5): 0.01 and 0.0012 µM LOQ (S/N = 10): 0.02 and 0.0024 µM Intra-assay (n = 10): 0.25±0.02 (CV 6.0%) and 57±3 (CV 6.0%) µM Inter-assay (n =5): 0.25±0.01(CV 4.0%) and 62±3.7(CV 6.0%) µM	Control (n = 25): 0.036—0.22 µM and 24–66 µM GAMT deficient: 14-15 µM and ND SLC6A8 deficient Cr levels: 56–62 µM	
Lipid Metabolism							

197	α-linolenoyl ethanolamide (α-LEA), palmitoleoyl ethanolamide (POEA), pentadecanoyl ethanolamide (PDEA), linoleoyl ethanolamide (LEA), anandamide (AEA), docosahexaenoyl ethanolamide (DHEA), 1-arachidonoylglycerol (1-AG), 2-arachidonoyl glycerol (2-AG), 1-linoleoyl glycerol (1-LG), 2-linoleoyl glycerol (2-LG), palmitoyl ethanolamide (PEA), dihomo-γ-linolenoyl ethanolamide (DGLEA), docosatetraenoyl ethanolamide (DEA), 1-oleoyl glycerol (1-OG), 2-oleoyl glycerol (2-OG), stearoyl ethanolamide (SEA), eicosa-pentaenoyl ethanolamide (EPEA), mead acid ethanolamide (ETAEA), noleoylethanolamine (OEA)	UPLC-QTrap, micro-LC-Qtrap, Phenomenex C18 column (2.6 µm, 0.3 × 150 mm). Eluents: A = 2 mM ammonium formate with 10 mM formic acid in water. B = ACN.Flow rate of 4 µL/min. Gradient: 55% eluent B and maintained for 0.5 min, eluent B was increased to 60% from 0.5 to 1.5 min, increased to 70% from 1.5 to 2.0 min, to 85% from 2.0 to 5.5 min, and increased to 95% at 5.6 min, where the gradient was kept until 8.0 min, then decreased to 55% eluent B at 8.1 min. A total analysis time of 16 min	250 µL CSF; ethanol (500 µL); 1 mL of MTBE, 50 µL 0.1 ammonium acetate solution at pH 4 buffer solution; drying; resuspension (20 µL of a mixture of water/ACN (1:1, v/v))	For validation: pooled CSF or water. For real sample analysis: CSF samples from healthy donors (n=94)	EMA guidelines. limits of quantification in range from 2.0 to 4311.3 pM, R2 >0.99; intra- and interday precisions were below 13.7%. The recoveries ranged from 61.5% to 114.8%. Matrix effects ranged from 24.4% to 105.2% . Internal standards N-(2-hydroxyethyl-1,1,2,2-d4)-9Z,12Z-octadecadienamide (LEA-d4), N-(2-hydroxyethyl-1,1′,2,2′-d4)-4Z,7Z,10Z,13Z,16Z,19Z-docosahexaenamide (DHEA-d4), N-(2-hydroxyethyl)-5Z,8Z,11Z,14Z-eicosatetraenamide-5,6,8,9,11,12,14,15-d8 (AEA-d8), 5Z,8Z,11Z,14Z-eicosatetraenoic-5,6,8,9,11,12,14,15-d8 acid (2-AG-d8), N-(2-hydroxyethyl)-hexadecanamide-7,7,8,8-d4 (PEA-d4), N-(2-hydroxyethyl)-octadecanamide-18,18,18-d3 (SEA-d3) and N-(2-hydroxyethyl-1′,1,2,2′-d4)-9Z-octadecenamide (OEA-d4)	Concentration of AEA in this CSF study for healthy controls ranged from 1.0 to 7.1 pM, and for 2-AG from 87.9 to 658.5 pM	Ethanol was used to stabilize the metabolites during long-term storage. Recovery and matrix effect were determined using deuterated internal standards. Major matrix effects were observed for several analytes, which may be caused by co-eluting phospholipids. As deuterated ISTDs were used, the quantification accuracy was ensured.

200	Free cholesterol, 17 free, non-esterified oxysterols and 17 free and conjugated bile acids	ESI (+) and ESI (-) UPLC-Qtrap. SunShell C18 analytical column. Total run time 23 min	55 µL CSF; 490 mL precipitation solvent adapted to different sample preparation protocols: ACN, MeOH, and iPrOH (containing 50 mg mL 1 BHT) were tested; on-line solid phase extraction	For validation: pure solvent. For real sample analysis: human CSF (n = 6)	EMA guidelines. Lin: 0.8-250 ng/mL for free hydroxycholesterols, 0.2-10 ng/mL for dihydroxycholesterols, 0.2-500 ng/mL for bile acids and 16-2000 mg/mL. Matrix factors 81-120%. No carry-over. Internal standards: 25 commercially available stable deuterium-labeled analogs of the analytes	In cerebrospinal fluid one free oxysterols, five free and five conjugated bile acids could be quantified.	In cerebrospinal fluid one free oxysterols, five free and five conjugated bile acids could be quantified. No significant differences between patients with and without blood–brain barrier disturbance were obtained. between-day precision was acceptable with coefficients of variation (CVs) ranging between 1.0% for the high abundant T-DCA and 22.2% for 7a-/7b-OHC at LLOQ. Furthermore, acceptable accuracy for most of the analytes was demonstrated through the % recovery values that were between 80% and 120% at all levels. Exceptions were mainly analytes for which no corresponding deuterated IS had been available: 20aOHC (153%), 7a,24S-diOHC (146%), 7a-HOCA (74%), or for analytes known to be susceptible to autoxidation: CH (128%), 5,6b-EC (125%), Triol (74%)

201	24(S)-hydroxycholesterol	2D-HPLC-QTrap, C18 guard column (4 × 3.0 mm, Phenomenex) as the first dimension at ambient temperature and Eclipse XDB-C18 (3 × 100 mm, 3.5 µm) as the second dimension at 50°C. For the first dimension LC, mobile phase A (0.1% formic acid in water) and mobile phase B [0.1% formic acid in isopropanolacetonitrile (1:2)] were operated with a gradient elution as follows: 0–0.6 min 60% B, 0.6–0.7 min 60–100% B, 0.7–5.5 min 100% B, 5.5–5.6 min 100–60% B, and 5.6–7.5 min 60% B at a flow rate of 0.6 ml/min. The solvent gradient for second dimension LC using 0.1% formic acid in water (phase C) and 0.1% formic acid in acetonitrile-methanol (1:4) (phase D) at a fl ow rate of 0.60 ml/min was as follows: 0–0.9 min 95% D, 0.9–6.0 min 95–100% D, 6.0–6.9 min 100% D, 6.9–7.0 min 100–95% D, and 7.0–7.5 min 95% D. Valve 1 was kept at the A position during 0–0.6 min and 1.2–7.5 min, and at the B position during 0.6–1.2 min. Valve 2 was kept	200 µL CSF; liquid–liquid extraction (1 mL methyl-tert-butyl ether); drying and derivatization (a solution of 63 mg of N,N'-diisopropylcarbodiimide, 62 mg of nicotinic acid, and 61 mg of 4-(dimethylamino)pyridine in 5 ml of chloroform)	For validation: 5% BSA.For real sample analysis: human CSF (n=3)	FDA guidelines. Lin: 0.025-5 ng/ml. Recovery >91%. No matrix effect. Precision and accuracy <13.3%.Various stabilities were assessed. Internal standard: 25,26,26,26,27,27,27-[2H7]24(R/S)-hydroxycholesterol (D7-24-HC)	No quantitative data	

		at the A position during 0–5.0 min and 7.0–7.5 min, and at the B position during 5.0–6.9 min. Total run time 7.5 min					
204	Androsterone, dihydrotestosterone, testosterone, allopregnanolone (5,3-THP), isopregnanolone, pregnenolone	GC-MS: 15 m × 0.25 mm × 0.05 µm	1–2 mL CSF; C18 SPE (50 µL 0.2% carboxymethoxylamine hemihydrochloride in pyridine); drying derivatization (100 µL 1.25% pentafluorobenzyl bromide + 2.5% diisopropylethylamine in MeCN + + 100 µL 50% BSTFA in MeCN).	For validation: saline solution. For real sample analysis: CSF samples from healthy volunteers and monkey	Lin.: 10–1000 pg/ml (R2 > 0.996). Recovery: 78.2–99.5%. Reproducibility (RSD): 4.6–35.0%. 2-month variation <10%. Internal Standard: progesterone-1,2,6,7-d4,5,3-THP-17,21,21,21-d4, 5,3-THDOC-17,21,21-d3, 5-DHP-1,2,4,5,6,7-d6, and 5-DHT-1,2,4,5-d4	Human CSF, pg/mL: androsterone – 52.8; testosterone – 158.3; allopregnanolone – 44.1; pregnenolone – 52.8. Monkey CSF, pg/mL: androsterone – 24.7; testosterone – 73.7; allopregnanolone – 6.3; pregnenolone – 16.7	
205	Pregnenolone, dehydroepiandrosterone, progesterone, androstenedione, testosterone, allopregnanolone, isopregnanolone, androsterone, epiandrosterone, 7α-hydroxy-dehydroepiandrosterone, 7β-Hydroxy-dehydroepiandrosterone, 5-androstene-3β,7α,17β-triol, 5-androstene-3β,7β,17β-triol, 16α-hydroxy-pregnenolone, 16α-hydroxy-dehydroepiandrosterone, 16α-	GC-MS: 15 m × 0.25 mm × 0.1 µm	1 mL CSF; extraction (3 mL of diethyl ether); drying; derivatization (methoxylamine-hydrochloride solution in pyridine (2%))	For real sample analysis: CSF samples from patients that underwent an endoscopic 3rd ventriculostomy because of obstructive hydrocephalus (n = 15)	Lin.: 10-1000 pg<br>Slope: 0.96–1.33<br>R: > 0.995<br>CV: 1.0–5.1%<br>LOD: 0.04–11.3 pM<br>Recovery: 75–104%	Median, nM: 0.060/0.078/0.235/0.208/0.231/0.008/0.040/0.005/0.004/0.300/0.037/0.007/0.012/0.001/0.006/0.072	Peripheral neuroactive steroids may be as good as the steroids in thecerebrospinal fluid for the diagnostics of CNS disturbances

	hydroxy-progesterone						
206	Dihydrotestosterone (DHT), testosterone, estrone (E1), estradiol (E2), dehydroepiandrosterone (DHEA), androstenedione (4D), progesterone (P)	GC-MS/MS: DB-17HT capillary column (15 m × 0.25 mm, 0.15 m)	450 µL CSF; 500 µL of 0.5 M ammonium acetate. Oximation of the keto groups was performed using triethylamine and pentafluorobenzylhydroxylaminehydrochloride. Esterfication was performed using pentafluorobenzoyl chloride	For validation: pooled CSF. For real sample analysis: CSF samples from healthy individuals (n=47)	Dihydrotestosterone (DHT, CSF lower limit of quantification, 1.5 pg/mL), testosterone (4.9), estrone (E1, 0.88), estradiol (E2, 0.25), dehydroepiandrosterone (DHEA, 38.4), androstenedione (4D, 22.3), and progesterone (P, 4.2). Inter- and intraassay precision: <10.9%. Accuracy: 83-120% Internal standards of DHT (dihydrotestosterone-2,3,4-13C3), testosterone (testosterone-2,3,4-13C3), E1 (estrone-2,3,4-13C3), E2 (estradiol-2,3,4-13C3), 4D (androstenedione-2,3,4-13C3), DHEA (dehydroepiandrosterone-2,2,3,4,4,6-d6), and P (progesterone-2,3,4-13C3)	Concentrations (pg/mL):DHT 2.21 ± 0.96; Testosterone 49.5 ± 18.9; E1 1.02 ± 0.29; E2 0.62 ± 0.34; DHEA 73.5 ± 31.7; 4D 61.4 ± 29.6; P <LLOQ	Testosterone in CSF is derived from circulating testosterone, DHT in CSF is from local conversion from testosterone, while E2 in CSF is from local conversion from 4D in CNS.
207	Dehydroepiandrosterone (DHEA), 7a-hydroxy-DHEA, 7b-hydroxy-DHEA, 7-oxo-DHEA, 16a-hydroxy-DHEA, cortisol and cortisone	ESI (+) UPLC-QQQ; Kinetex C18 2.6 µm (150 x 3.0 mm) column; flow rate 0.75 mL/min. Eluents: A = water; B= MeOH. 0 min, 50:50 (A:B); 2 min, 50:50; 4 min, 25:75; 7 min, 5:95; 8.5 min 5:95; 9 min, 50:50; 10 min, 50:50 and at 10 min stop	3 mL CSF; dilution (1 mL physiological solution); extraction (4 mL diethyl ether); frying; derivatization (2-hydrazinopyridine, MeOH and trifluoroacetic acid (1 mg: 5 mL: 1.63 µL))	For real sample analysis: CSF samples from subjects with excluded normal pressure hydrocephalus (n = 37)	Lin (ng/mL): DHEA 0.008–1; 2–28 pg/mL; Cortisol 0.8–100; Cortisone 0.2–25; 7a-OH-DHEA 0.016–2; 7b-OH-DHEA 0.008–1; 7-oxo-DHEA 0.008–1; 16a-OH-DHEA 0.008–1 (R2 >0.998). Within-day precision; 0.63–5.48%; between-day precision: 0.88–14.59% and recovery: 85.1–109.4%. No matrix effect. Internal Standard: D3-DHEA and D4-cortisol	Concentrations, ng/mL: DHEA 0.004–0.610; Cortisol 0.808–20.20; Cortisone 0.137–5.028; Cortisol/cortisone 1.15–10.31; 7a-OH-DHEA 0.016–0.315; 7b-OH-DHEA 0.003–0.041; 7-oxo-DHEA <LOD; 16a-OH-DHEA 0.005–0.027	The method enables simultaneous quantification of steroids important for the estimation of 11b-hydroxysteroid dehydrogenase activity in human plasma and CSF

208	Estriol (E3), estrone (E1), 17b-estradiol (17b-E2), and 17a-estradiol (17a-E2)	ESI (+) two-dimensional LC-MS/MS; three analytical columns, including a Varian Polaris Amide-C18 (2 × 100 mm, 3 mm, 200 A) and two Shimadzu Shim-pack XR-ODS columns (2 × 100 mm and 2 × 75 mm, 2.2 mm, 120 A). Eluents: A = H2O/ACN/formic acid:95/5/0.1, B = ACN/H2O/formic acid:95/5/0.1.	1 mL; extraction (2 mL ethyl acetate); drying; derivatization (50 µL of dansyl chloride)	For validation: charcoal-stripped CSF. For real sample analysis: CSF of ischemic trauma patients (n=3)	Lin. 50 to 300 pg/mL for E3 and from 20 to 300 pg/mL for E1, 17b- and 17a-E2 (R2> 0.996). LODs E1, 17a-E2, 17b-E2, and E3 were 19, 35, 26, and 61 pg/mL, respectively. The precision and accuracy were more than 86% for the two E2 compounds and 79% for E1 and E3 while the extraction recovery ranged from 91% to 104%. No matrix effects. Internal Standards: estrone-2,4,16,16-d4 (E1-d4); 17b-estradiol-16,16,17-d3 (17bE2-d3); estriol-2,4-d2 (E3-d2), and 17a-estradiol-2,4-d2 (17a-E2-d2)	17b-E2 was quantitatively measured in 2/3 samples.	Dansylated estriol and estrone were separated in the first dimension by an amide-C18 column, while dansylated 17b- and 17a-estradiol were resolved on the second dimension by two C18 columns (175 mm total length) connected in series.
Vitamin B6							
212	Pyridoxal (PL), pyridoxamine (PM), pyridoxine (PN), pyridoxic acid (PA), pyridoxal 5′-phosphate (PLP), pyridoxamine 5′-phosphate (PMP) and pyridoxine 5′-phosphate (PNP)	ESI (+) UPLC-QQQ; Acquity HSS-T3 column (1.8 µm, 2.1 mm × 5 mm). A two-step (linear) gradient of 3.5 minutes at a flow rate of 0.4 mL/min between solvent A (650 mM acetic acid with 0.01% HFBA) and solvent B (100% acetonitrile) was used. The gradient started with 100% solvent A. Between 0.5 min and 2.0 min it changed to 80% solvent A. In 0.1 min the gradient switched to 100% solvent B, which was maintained during the next 0.2 min. A direct switch to	60 µL CSF; protein precipitation (trichloroacetic acid)	For real sample analysis: CSF from patients who were investigated for developmental delay (n = 14), movement disorder (n = 2) or both (n = 4)	Lin. Recoveries between 93% and 96%. Intra- and inter-assay variations below 20%. Accuracy tests showed deviations from 3% (PN) to 39% (PMP). Limits of quantification were in the range of 0.03–5.37 nM. Poor results were obtained for quantification of PNP. Various stabilities were assessed. Internal standards: PL-D3 for PL and PM, PN-13C4 for PN, PA-D2 for PA and PLP-D3	Concentrations (nM): PL (14.8–42.5), PM (0.1–0.5), PA (0.09–4.1) and PLP (8.8–42.0)	

		100% solvent A was made and 1.2 min was used for column equilibration					
213	pyridoxal 5-phosphate (PLP), pyridoxal (PL), pyridoxine (PN), pyridoxamine (PM) and pyridoxic acid (PA)	ESI (+) UPLC-QQQ Waters BEH C18 (100 × 2.1 mm 1.7 µm) column. The mobile phase A consisted of water with 0.1 % formic acid and methanol as the mobile phase B. Mobile phase A was prepared freshly for each analytical run. A flow rate of 0.2 mL/min was used and the following mobile phase gradient was applied (%B): 0–0.1 min, 5 %; 2.7 min, 35 %; 2.9–3.25 min, 95 %; 3.4–4.7 min, 5 %.	50 µL CSF; protein precipitation (200 µL ACN/MeOH (9:1, v/v) containing 0.1 % formic acid)	For validation: Ringer solution. For real sample analysis: CSF from children who underwent lumbar puncture for work up/follow up in neurometabolic disorders (n=156)	ICH guideline M10. Lin. 5 to 200 nmol/L (R2> 0.99). The accuracy 95.2-105.2 %; precision 1.6 -11.2 %. The recovery of the surrogate matrix approach was within 82.4 % (PA) and 97.6 % (PLP) with CV<20 % from 3.9 % (PN) to 10.5 % (PM). Internal standard: pyridoxamine-D3, pyridoxine-D2, pyridoxal-D3 , pyridoxal 5-phosphate-D3, pyridoxic acid-D3	Concentrations, median, nM: pyridoxal 15; pyridoxal 5-phosphate 20.	the analytical conditions and obtained results were compared with several previously described studies
